# Merging Signaling with Structure: Functions and Mechanisms of Plant Glutamate Receptor Ion Channels

**DOI:** 10.1146/annurev-arplant-070522-033255

**Published:** 2023-02-28

**Authors:** Alexander A. Simon, Carlos Navarro-Retamal, José A. Feijó

**Affiliations:** 1 Department of Cell Biology and Molecular Genetics, University of Maryland, College Park, Maryland, USA; 2 Department of Anesthesiology, Weill Cornell Medicine, New York, NY, USA

**Keywords:** plant glutamate receptor, GLR, ion channels, excitability, cell–cell communication, calcium transport, calcium signaling, electric signaling, action potential, glutathione, amino acid signaling

## Abstract

Plant glutamate receptor-like (GLR) genes encode ion channels with demonstrated roles in electrical and calcium (Ca^2+^) signaling. The expansion of the GLR family along the lineage of land plants, culminating in the appearance of a multiclade system among flowering plants, has been a topic of interest since their discovery nearly 25 years ago. GLRs are involved in many physiological processes, from wound signaling to transcriptional regulation to sexual reproduction. Emerging evidence supports the notion that their fundamental functions are conserved among different groups of plants as well. In this review, we update the physiological and genetic evidence for GLRs, establishing their role in signaling and cell–cell communication. Special emphasis is given to the recent discussion of GLRs’ atomic structures. Along with functional assays, a structural view of GLRs’ molecular organization presents a window for novel hypotheses regarding the molecular mechanisms underpinning signaling associated with the ionic fluxes that GLRs regulate. Newly uncovered transcriptional regulations associated with GLRs—which propose the involvement of genes from all clades of *Arabidopsis thaliana* in ways not previously observed—are discussed in the context of the broader impacts of GLR activity. We posit that the functions of GLRs in plant biology are probably much broader than anticipated, but describing their widespread involvement will only be possible with (*a*) a comprehensive understanding of the channel’s properties at the molecular and structural levels, including protein–protein interactions, and (*b*) the design of new genetic approaches to explore stress and pathogen responses where precise transcriptional control may result in more precise testable hypotheses to overcome their apparent functional redundancies.

## 1. INTRODUCTION

Plant glutamate receptor-like (GLR) ion channels are ionotropic receptors, ion channels that are activated by ligand binding. Akin to their mammalian homologs, GLRs are sometimes referred to simply as glutamate receptors. However, in plants, the range of ligands for GLRs is much broader than just glutamate, and GLRs’ physiological repertoire in plant biology has been steadily increasing in recent years. The interest and scrutiny of these amino acid–receptor channels are proportional to the number of reviews covering their characterization, from the initial steps of identification and evolution (27, 40, 44, 118, 158) to more focused aspects such as roles in long-distance communication (47), structural and functional comparison to animal homologs (165), and physiological functions (48).

GLRs are named according to their homology to the mammalian ionotropic glutamate receptors (iGluRs), which are ligand-gated ion channels (85, 147). iGluR specificity for glutamate binding activates ion-selective transport that is key to neurotransmission (50, 56, 147). As plants lack an anatomical nervous system and GLRs have been documented in diverse physiological processes across plant organs, the functional definition of GLRs is not fully understood. The uniting factor of GLRs and iGluRs is their control over electrical signaling and calcium (Ca^2+^) signaling. However, major gaps in GLR knowledge exist in fundamental areas such as ion selectivity and channel gating properties, which are critical parameters underpinning signal transduction in a cell–cell communication context. The localization of GLRs in various subcellular structures other than the plasma membrane [e.g., endoplasmic reticulum (ER), tonoplast, and plastids] also diverges from the canonical iGluR function in plasma membrane–based cell–cell communication, suggesting that new roles may have evolved, such as Ca^2+^ store operation, for which plants lack practically all the canonical small-ligand regulatory systems described in animals [e.g., ryanodine receptors and inositol triphosphate (IP3) receptors]. The recent burgeoning understanding of GLRs’ molecular detail provided by various atomic structures, including the ligand-binding domain (LBD) (2, 42) and the full-length channel of *At*GLR3.4 (45), offers a new lens through which to examine the available genetic and physiological evidence for GLR function and extract hypotheses both to propel the plant membrane transport field and to enlarge the palette of GLR-associated roles in plant physiology.

## 2. GLUTAMATE RECEPTOR HOMOLOGY AS SEEN BY ATOMIC STRUCTURE

**Figure 1:**
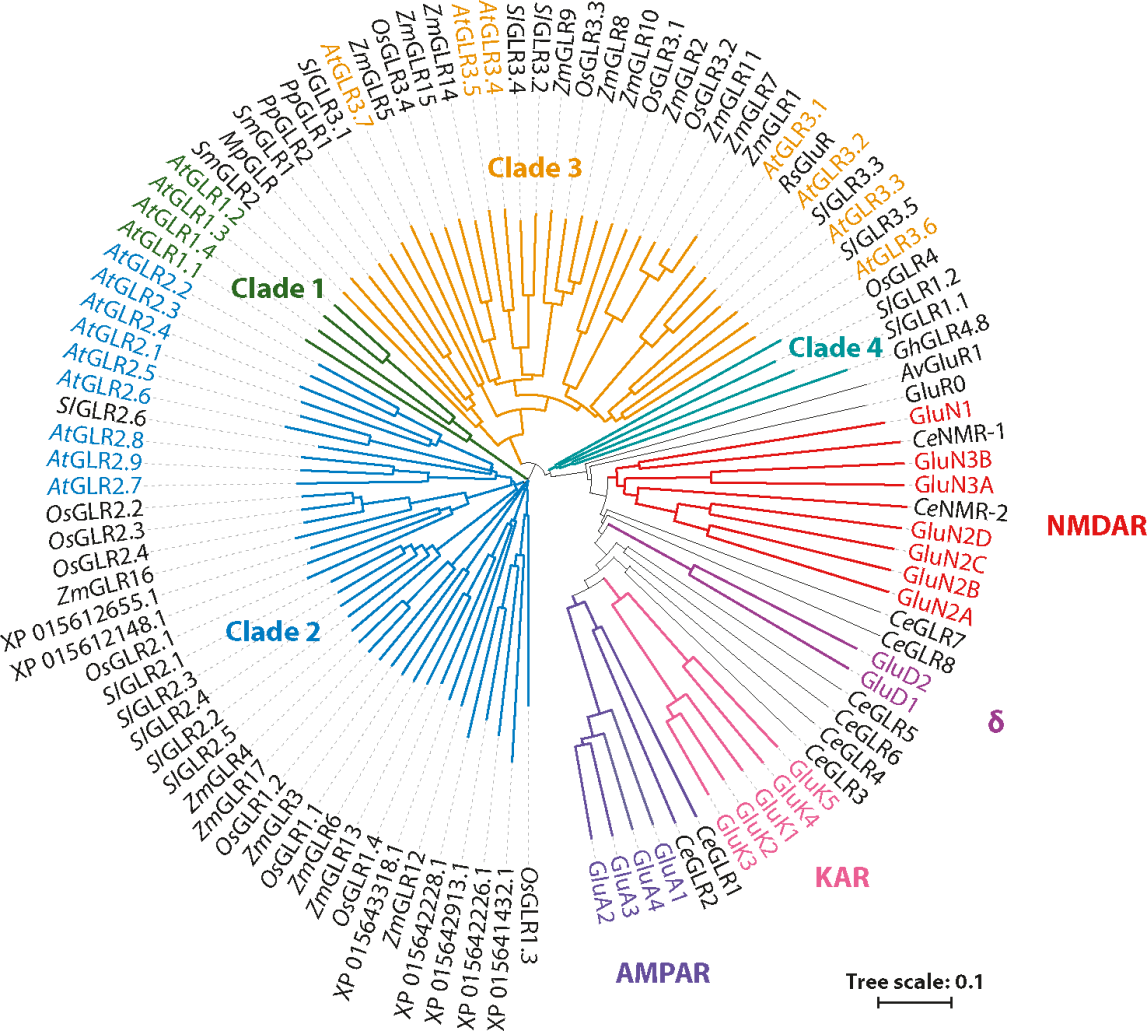
Phylogenetic relations of plant GLRs and iGluRs. The phylogenetic relations of glutamate receptors from the model flowering plant of *Arabidopsis thaliana* (*At*) and other flowering plants that are described to show a conserved phenotype, including *Zea mays* (*Zm*), *Oryza sativa* (*Os*), *Solanum lycopersicum* (*Sl*), *Gossypium hirsutum* (*Gh*), and *Raphanus sativus* (*Rs*), as well as basal land plants such as the moss *Physcomitrium patens* (*Pp*), the liverwort *Marchantia polymorpha* (*Mp*), and the lycophyte *Selaginella moellendorffii* (*Sm*), compared to the invertebrate *Caenorhabditis elegans* (*Ce*) and AMPARs, NMDARs, KARs, and δ-receptors from mammals (without prefix). Also included are the bacterial GluR0 from *Synechocystis PCC 6803* and *Av*GluR1 from the freshwater rotifer *Adineta vaga* (*Av*). GLRs from *A. thaliana* and mammals are shown in colored text for clarity. Proteins identified with the prefix XP are unannotated sequences from *O. sativa* containing signature GLR motifs. Sequences were aligned using MUSCLE software, and the phylogenetic tree was constructed using the neighbor-joining method (35, 126). Sequences are available in the Supplemental Text, and selected accession numbers are included in the Supplemental Text or Table 1. Additional abbreviations: AMPAR, α-amino-3-hydroxy-5-methyl-4-isoxazolepropionic acid receptor; GLR, glutamate receptor-like; iGluR, ionotropic glutamate receptor; KAR, kainate receptor; MUSCLE, multiple sequence comparison by log-expectation; NMDAR, *N*-methyl-d-aspartate receptor; δ, δ-receptor.

The glutamate receptor family members are evolutionarily conserved ion channels that are only absent in archaebacteria and fungi (20, 43, 64, 99, 122, 133). The model flowering plant *Arabidopsis thaliana* encodes 20 GLRs divided into three phylogenetic clades (84) (Figure 1). Clade 3 is the most ancestral, while clade 1 is the most recently diverged group and is specific to Brassicaceae members (28). Clade 2 has been described as a sister to clade 1 (20). Absent from *Arabidopsis* is the novel clade 4, which appears rare and includes many unannotated and largely uncharacterized genes (4, 28, 89). Of relevance, a dramatic multiplication of genes and the multiclade structure made their appearance with flowering plants (28, 84, 165) (Figure 1). For comparison, *Arabidopsis* clades 1 and 2 diverged more from clade 3 than clade 3 diverged from the moss *Physcomitrium patens* and the lycophyte *Selaginella moellendorffii*, despite hundreds of millions of years of divergence. Without apparent preference for clade assignment, GLRs in flowering plants are expressed throughout the whole plant and are proposed to exhibit widespread subcellular localization to the plasma membrane, vacuolar system, ER, plastids, and mitochondria (45, 102, 111, 143, 144, 153, 166) (Table 1). Notwithstanding the existence of possible reported localization artifacts from overexpression when constitutive promoters are used, most of the reported localizations were done with native promoters, and in a few cases with antibodies, suggesting that localization to the plasma membrane and other endomembranes is agreed upon among researchers. For example, *At*GLR3.5 is the only GLR gene with a signal sequence cognate to localization in mitochondria and plastids, which was functionally confirmed (143). Localization of other GLR members seems to need specific membrane-sorting mechanisms from the ER. For example, *At*GLR2.1 sorts to the vacuole in pollen and, in the extreme, *At*GLR3.3 localizes to the external and plasma membranes of sperm but not to the pollen tube membrane a few micrometers away, despite localizing to the plasma membrane in root cells (152, 153, 166). Both of these GLRs were found to depend on the coat protein complex II (COPII) cargo adaptor CORNICHON-HOMOLOG (CNIH) family of proteins (see Section 3.1) and become retained in the ER when two or more CNIH genes are mutated (165, 166). Last but not least, the multiplicity of GLR genes and their discrete groupings thus pose immediate but long-standing questions if new genes arose by duplication events or if divergent clades adopted original attributes by strong evolutionary pressure to co-opt novel functions that are not present in the mosses, ferns, or early land plants (27). Neither outcome is mutually exclusive, and mapping of tandem genes on the same chromosome suggests the existence of duplication events.

### 2.1. The Domain Architecture of Ionotropic Glutamate Receptors

**Figure 2:**
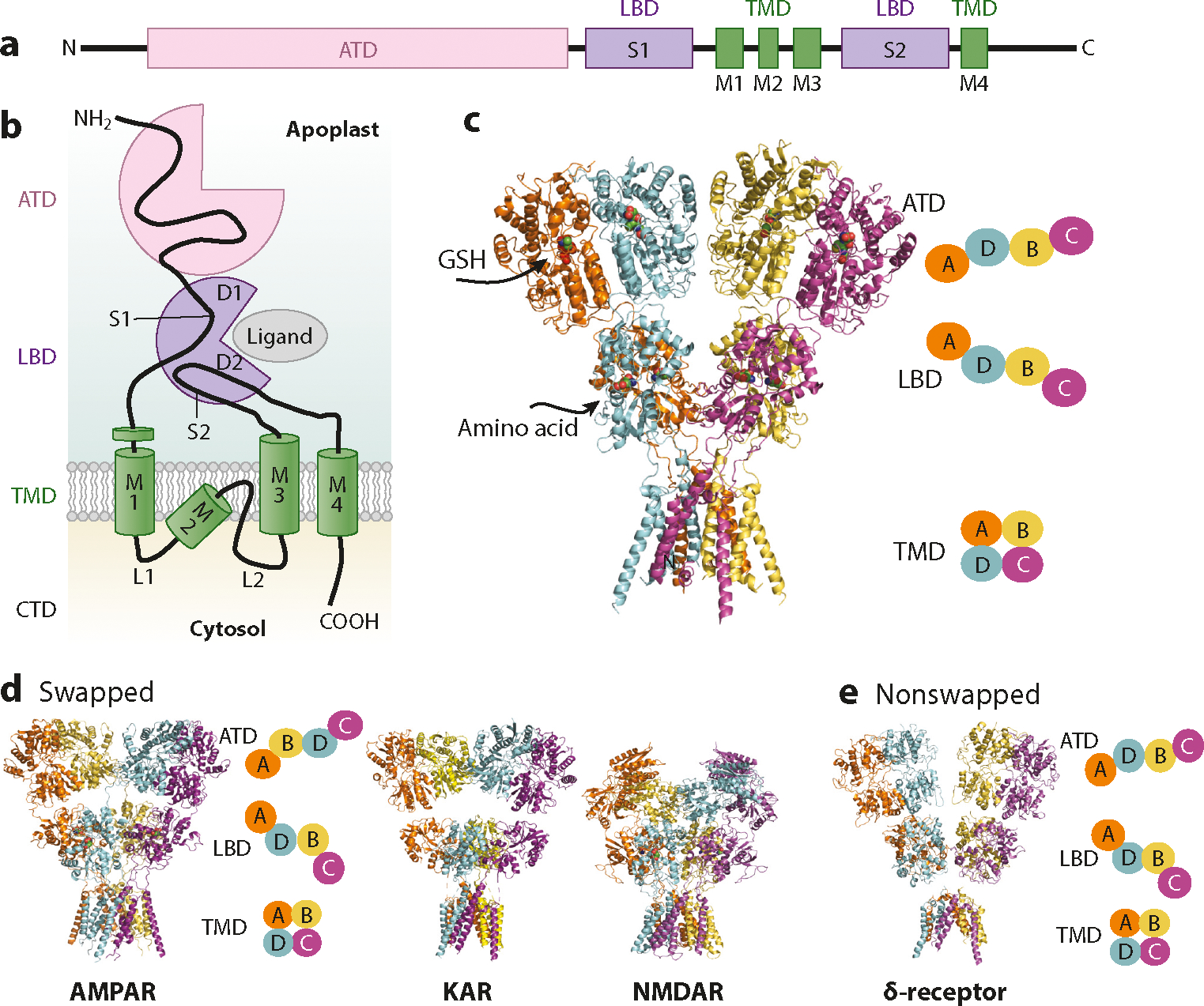
GLR architecture. (*a,b*) Linear representation of common GLR domains and GLR subunit topology showing the three main structural and functional protein domains: ATD, LBD, and TMD with the CTD. (*c*) Structure of the AtGLR3.4 homomer (PDB ID 7LZH) next to a diagram displaying the paired subunits in the TMD, LBD, and ATD. (*d,e*) Common architecture representative of AMPAR, NMDAR, KAR, and δ-receptors (PDB IDs 5WEO, 5IOV, 7KS3, and 6KSS, respectively). Diagram displaying the paired subunits appears as in panel *c*. Abbreviations: AMPAR, α-amino-3-hydroxy-5-methyl-4-isoxazolepropionic acid receptor; ATD, amino-terminal domain; CTD, C-terminal domain; GLR, glutamate receptor-like; GSH, glutathione; KAR, kainate receptor; LBD, ligand-binding domain; NMDAR, N-methyl-d-aspartate receptor; PDB ID, Protein Data Bank identifcation; TMD, transmembrane domain; δ, δ-receptor.

The homology between GLRs and iGluRs was discovered by complementary DNA sequences revealing a predicted topology consisting of a transmembrane domain (TMD)—comprised of three transmembrane pass α-helixes plus one partial reentry pore loop—along with a putative LBD that is conserved with bacterial periplasmic amino acid–binding proteins (PBPs) and animal iGluRs (1, 85). The atomic structures of isolated LBDs of *At*GLR3.2 (42), *At*GLR3.3 (2), and *At*GLR3.4 (45) captured by X-ray crystallography plus the full-length channel of *At*GLR3.4 solved by cryo-electron microscopy (cryo-EM) (45) validate the evolutionary relationship between GLRs and iGluRs. GLRs, like iGluRs, form ion channels by tetramerization, showing a similar modular layout (45) (Figure 2). The TMD layer, which contains the ion channel pore, is made up of three transmembrane-spanning domains (M1, M3, M4) and one reentry pore loop (M2), reminiscent of canonical iGluRs. The M1, M2, and M3 domains are concatenated by a pair of intracellular linkers, L1 and L2, although these segments are not yet characterized. The TMD is attached to the LBD by another set of polypeptide linkers (S1-M1, M3-S2, S2-M4). Found in the apoplastic extracellular space, the LBD is encoded by the S1 and S2 segments of each gene and translated into a clamshell-shaped receptor with an upper (D1) and a lower (D2) globular lobe. An amino-terminal domain (ATD) layer sits on top of the LBD. Lastly, a carboxyl-terminal domain (CTD) is present intracellularly (Figure 2).

M3 is the most highly conserved domain of the protein, harboring the consensus sequence SYTAxLxxx (21). Coined as the ion channel gate, four M3 helixes achieve a fourfold symmetry converging in a conic shape pointing inwards and marking the extracellular border of the ion permeation pathway crossing the lipid membrane (149, 150). The SYTAxLxxx motif is entirely conserved in *Homo sapiens* represented by SYTANLAAF and is essential for iGluR gating (147). Plant GLRs differ in four of the eight amino acids, with consensus motifs also diverging between the three clades (165). While the conservation of the SYTAxLxxx motif would suggest an important functional role, there is no data illustrating if these divergences in GLRs reflect any functional adaptation.

An important uniqueness of GLRs comes from the M2 pore loop, which is critical for GLR function since it comprises the pore and selectivity filter (largely governing the ion selectivity). The amino acid residues within GLRs’ M2 pore loops are dramatically different from any within iGluR families (27, 31). This divergence makes any prediction by sequence comparison with mammalian channels (or for any other known ion channel) futile in terms of predicting what ions pass—inward or outward—through GLRs (27, 31). While the three-clade system of *At*GLRs is well-rooted in their sequence divergence, if only the channel’s transmembrane M1 and M3 domains with the M2 pore loop are analyzed, then the clade separation disappears (20, 44), suggesting a conservation of ion selectivity among plant GLRs of different clades. We presently have no experimental evidence for this, and, in the absence of any evident patterns regarding functional differences between clades, the strong divergence of the pore region of GLRs from other glutamate receptors (mammalian and prokaryotic) remains counterintuitive. As a result, best guesses would logically conclude that the extracellular domains of the LBD and ATD harbor the most divergence separating plant GLR clades and therefore could underlie diverse ligand-gating properties and general channel regulation, while ion selectivity between clades should be similar. Presently available data allow no confirmation or falsification of these general principles.

### 2.2. The Evolution of an Amino Acid–Signaling Ion Channel

**Figure 3:**
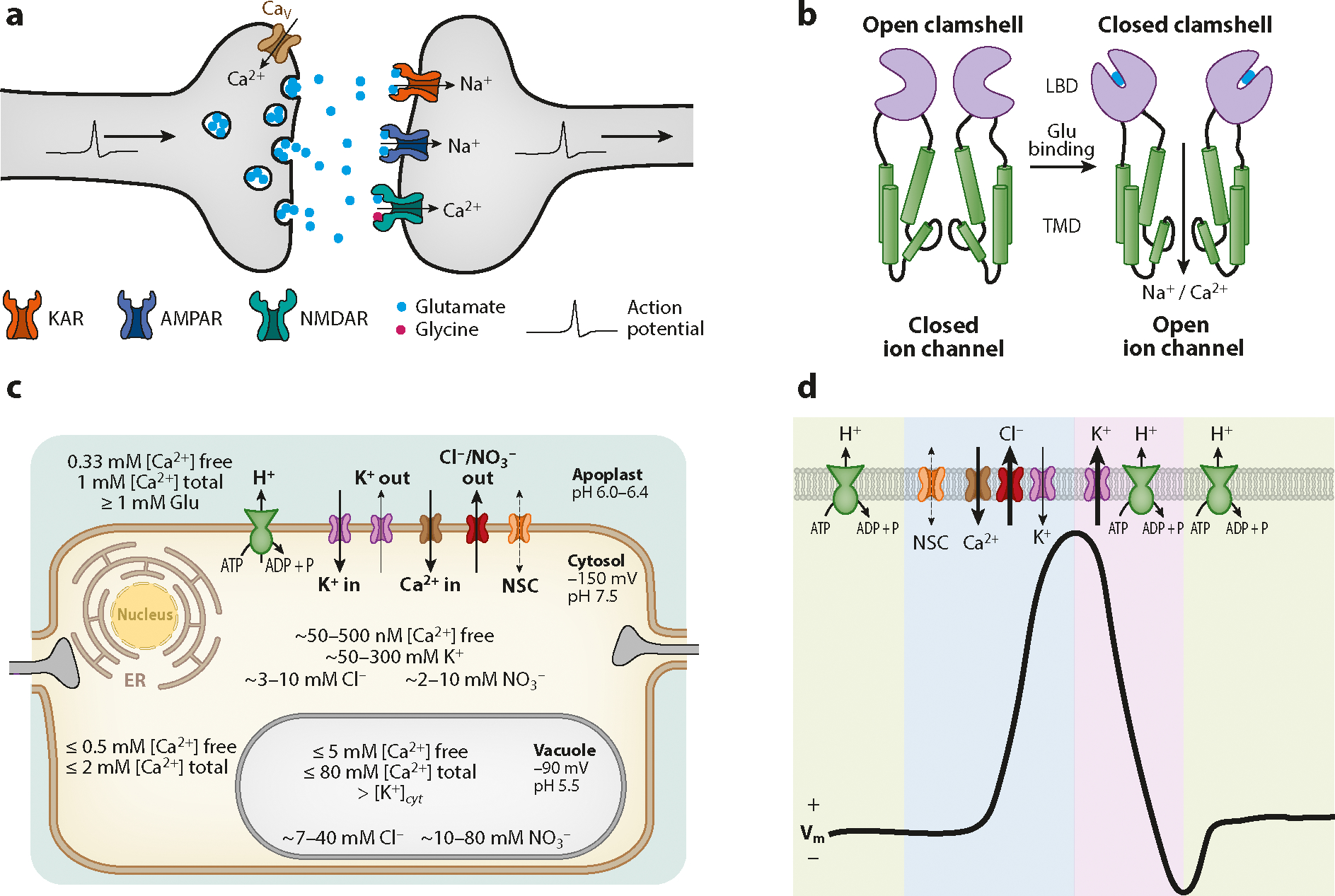
Different environments for iGluRs and GLRs. (*a*) High-fdelity synaptic transmission moderated by Glu and iGluRs at the postsynaptic membrane. (*b*) Minimal iGluR ligand-gating scheme underpinning neuronal activity. (*c*) Ion concentrations of the typical plant cell at rest and putative ion channels/transporters that regulate ion transport. (*d*) Ionic basis of electrical signaling in plants for maintaining the resting membrane potential and evoking depolarization and repolarization. In panels *c* and *d*, arrows represent fuxes of ions either determined (*solid arrows*) or conceptually predicted (*dashed arrows*). Arrow thickness depicts ion fux intensity, where K^+^ and Cl^−^ carry the bulk of the repolarization/depolarization, inward/outward currents, respectively. Abbreviations: ADP, adenosine diphosphate; AMPAR, α-amino-3-hydroxy-5-methyl-4-isoxazolepropionic acid receptor; ATP, adenosine triphosphate; Ca^2+^, calcium; Ca_v_, voltage-gated Ca^2+^ channel; Cl^−^, chloride; ER, endoplasmic reticulum; GLR, glutamate receptor-like; Glu, glutamate; iGluR, ionotropic glutamate receptor; H^+^, protons; K^+^, potassium; KAR, kainate receptor; LBD, ligand-binding domain; Na^+^, sodium; NMDAR, *N*-methyl-d-aspartate receptor; NO_3_^−^, nitrate; NSC, nonselective channel; P, phosphorus; TMD, transmembrane domain; V_m_, membrane potential.

The pivotal step in the evolution of all glutamate receptors appears to have come from the fusion of a prokaryotic potassium (K^+^) ion channel pore loop with a bacterial amino acid–binding protein (162). The acquisition of an amino acid receptor by a transmembrane ion channel indicates that these ionotropic receptors evolved to operate in amino acid signaling. The acquisition of a second amino acid binding that forms the ATD (see Figure 2) would be expected to emphasize the apparent importance of amino acid binding (1, 165). However, there is no evidence of amino acid binding to the GLR ATD. Several allosteric modulators, including zinc (Zn^2+^), protons (H^+^), and ifenprodil, have been discovered to bind the ATD of some human iGluRs, making it a strong regulatory domain and candidate site for drug design (63). From an evolutionary perspective of the TMD, it seems increasingly apparent that the ion selectivity governed by the pore has greatly diversified between kingdoms. Experimental data foremost support the hypothesis that the pore loop is derived from an unknown K^+^ channel, creating a phylogenetic link between glutamate receptors and K^+^ channels (81, 163). The bacterial glutamate receptor GluR0 from *Synechocystis* is only a three-transmembrane pass ion channel, missing the M4 domain in addition to lacking the ATD that was later developed in eukaryotes. GluR0 is also a K^+^-selective ion channel (18). The freshwater rotifer *Adineta vaga* is thought to encode the first eukaryotic glutamate receptor with a modular architecture and four-transmembrane pass channel similar to those of GLRs and iGluRs and is also a K^+^-selective ion channel (64). Among mammals, iGluRs evolved to be nonselective cation channels, many with permeability to Ca^2+^. The nonselectivity of iGluRs—and relative gain of sodium (Na^+^) permeability—was one adaptation to promote membrane depolarization of neurons and comprises their main physiological role (165) (see the sidebar titled Neurotransmission). An exact comparison cannot be drawn to plant GLRs, given that anions are involved in the main depolarization mechanisms of plants. Theoretically, anionic permeability of GLRs would fulfill the same physiological role that Na^+^ permeability of iGluRs does to drive membrane depolarizations for electrical signaling (see the sidebar titled Different Environments: Do Plant Glutamate Receptors Function Like Ionotropic Glutamate Receptors?) (see also Figure 3). Presently, there is, however, no data to support such a hypothesis.

## 3. PLANT GLUTAMATE RECEPTORS ARE INVOLVED IN ELECTRICAL AND CHEMICAL SIGNALING

Given their centrality in neurobiology, iGluRs have been the focus of a large research community over the past few decades, which has expanded the knowledge about iGluR mechanisms in every possible cutting-edge direction, from physiology to atomic structure (50, 170). Unsurprisingly, plant GLR research has been strongly impinged by these paradigms, particularly in areas of ligand gating and glutamate-specific signaling. Consequently, many of the screenings for GLR functions in plants have focused on the established roles of iGluRs in cell–cell communication, such as electrical (through a depolarization of the membrane) or Ca^2+^ signaling. Such studies in plants now comprise pharmacology, reverse genetics, Ca^2+^ imaging, and various electrophysiology techniques, including the use of heterologous expression in animal single-cell systems, which warrant the conclusion that GLRs also evolved electrical and Ca^2+^ signaling functions.

### 3.1. Genetic Evidence for Plant Glutamate Receptors in Electrical and Ca^2+^ Signaling

The major advancement in characterizing GLRs came from reverse genetics. Genetic analysis yielded reproducible evidence that GLR phenotypes are prominently associated with a channel’s role in membrane depolarization and the generation of Ca^2+^ signals (Table 1). Genetic analyses became powerful tools complementing the widespread use of classical iGluR pharmacology to challenge plant GLRs (29, 30, 32, 78, 83, 85).

The *Arabidopsis* GLRs of clade 3 have been particularly amenable to genetic characterization. Intracellular membrane potential measurements revealed that amino acid stimulation of mutants for *Atglr3.3* or *Atglr3.4* had weaker depolarization amplitudes; while the exact cells/tissues impaled were not defined in these experiments, imaging also revealed abolished elevations in the free cytosolic Ca^2+^ concentration ([Ca^2+^]_*cyt*_) in the case of *Atglr3.3* (2, 120, 137). The impalement of roots with electrodes putatively recorded from cortical cells outside the phloem also shows a typical waveform with attenuated depolarizations and incomplete repolarization (120, 137). In response to mechanical wounding or herbivory in leaves, depolarization of the membrane potential detected by surface potential measurements preceded an elevation of [Ca^2+^]_*cyt*_ that is suppressed by mutations of *Atglr3.1*, *Atglr3.2*, *Atglr3.3* or *Atglr3.6* (107, 111). The double-mutant knockout of *Atglr3.3 Atglr3.6* renders the most potent phenotype, showing a nearly complete loss of membrane depolarization and [Ca^2+^]_*cyt*_ elevation at distal leaves. *At*GLR3.3 and *At*GLR3.6 thus appear to be the synergistic master regulators, despite not showing colocalization (37, 105, 107, 111, 127, 128, 135, 146, 151). Suppression of *Atglr3.3 Atglr3.6* also attenuated light-induced or wound-induced reactive oxygen species (ROS) accumulation in response to light (38, 86).

GLR-dependent electrical wound signals are based on cell–cell communication and can travel long distances to a systemic scale, propagating to neighboring leaves and roots (111, 127, 135). The characteristic wound-induced root-to-shoot phenotype was shown to be evolutionarily conserved in rice (*Oryza sativa*, *Os*), and dependent on *OsGLR3.4*. In tomato (*Solanum lycopersicum*, *Sl*), wound-induced root-to-shoot signaling was shown to be dependent on *SlGLR3.5*, a close homolog of *AtGLR3.6*, thus phenocopying the *Atglr3.3 Atglr3.6* double-mutant knockout (156, 169). Remarkably, changes in the phloem membrane potential within the wounded leaf were not affected by *Atglr3.3 Atglr3.6* knockouts (111, 127).

Measurements by whole-plant electrical penetration graph (EPG), a technique that measures gross potentials across leaf phloem by forming an electric circuit through aphid penetration and soil electrodes, were also used to characterize these potentials (128). Mild caterpillar feeding wounds in the phloem induced fast depolarization of +60 mV within 2 s on the eaten leaf. Interestingly, these fast waves induced jasmonate-related genes locally but not in neighbor leaves, which only saw jasmonate-related genes induced after major damage and membrane potential changes with a slower component (indicative of the potential change in the root) and less intense amplitude (approximately +30 mV) (127, 128). In short, all electrical activity was suppressed in the *Atglr3.3 Atglr3.6* double mutant. EPG was further used to show a role for *AtGLR3.5* in regulating excitability in wound-induced potentials but only along leaf phloem networks, showing no differences in the potentials from roots (127). Depending on the leaf development patterning (numbered to correspond to the underlying vascular anatomy), plants exhibiting *Atglr3.5* expression knockdown showed either a reduced wound-induced potential amplitude or new signals not seen in the wild type, suggesting intricate regulatory mechanisms (127). Differences in cellular and subcellular localization, ion channel gating, and ion selectivity have yet to be fully elucidated among these three channels and may offer explanations for this phenotypic difference. In that regard, it should be noted that *At*GLR3.5 is unique among the 20 *Arabidopsis* GLRs as the only one that harbors a cognate signal peptide targeted to plastids and mitochondria (143, 165).

Clades 1 and 2 have been the subject of fewer genetic investigations.*At*GLR1.4 has been shown to affect the plasma membrane potential in cotyledons upon exposure to amino acids (140). Genetic analysis in the pollen tube stands out for having attributed a role for GLRs from every clade in pollen tube growth and morphogenesis associated with Ca^2+^ extracellular fluxes (45, 104, 166). So far, 9 of the 20 genes encoding GLRs in *Arabidopsis* have been documented in the single-celled pollen tube system. GLR documentation in pollen tubes also demonstrated for the first time the impact of subcellular localization to membranes other than the plasma membrane as well as the heavy involvement of CNIHs. *CORNICHONS* (*CNIs*) were originally cloned in *Drosophila*, and their mutant is embryo lethal by affecting neuronal function; later they were found to have a dual role as (*a*) a cargo adaptor in the COPII ER-secretion pathway to export iGluRs to the plasma membrane and (*b*) a modulator of glutamate receptor channel activity (see Section 4.3) (9, 134, 138). Such functions were found to be conserved in plants. With *Arabidopsis* bearing a family of five members, CNIHs were shown to traffic *At*GLRs, establishing localization to the plasma membrane or endomembranes in the pollen tube (165, 166). Importantly, plants lack a small ligand-operated Ca^2+^ store system such as those in all other kingdoms (34), so differential subcellular localization of GLRs bears the potential to play a role in cytosolic Ca^2+^ homeostasis by coordinating fluxes from different reservoirs, including the apoplast, vacuole, or ER. CNIHs also act on channel activation, as CNIH co-expression converted inert *At*GLR3.3 and *At*GLR3.2 into functionally active ion channels during heterologous expression (166). Still to be determined is the effect of specific CNIH isoforms and their assignment to trafficking tasks, roles in channel activation, or roles in preferentially targeting specific GLRs. Intriguingly, pairs of mutations of any two CNIHs are sufficient for disrupting the trafficking of GLRs from the ER, while single mutations produce no effect (166). However, heterologous co-expression of a single CNIH is sufficient to strongly increase currents mediated by *At*GLR3.2 and *At*GLR3.3 (166). These nonobvious effects warrant further dissection of the genetic interactions between GLRs and CNIHs.

Recent breakthroughs from screening differential GLR expression levels within various tissues of *Arabidopsis* unearthed the involvement of clade 1 and clade 2 to a much greater extent. In studies using assay for transposase-accessible chromatin with high-throughput sequencing (ATAC-seq) and RNA sequencing (RNA-seq) after root wounding by root cap excision, the GLR family stood out as one of the fastest and most dynamically affected with genes from all three GLR clades showing dramatic variations (55). These experiments provided evidence that *AtGLR3.3*–*AtGLR3.7* have reduced expression in the few hours postwounding, while *AtGLR1.2*, *AtGLR1.4*, *AtGLR2.2*–*AtGLR2.4*, and *AtGLR2.9*–*AtGLR3.2* expression was elevated 24 h postwounding, outlining distinct short-term and long-term transcriptional regulation (see Table 1). Upon root cap excision, the Ca^2+^ component of the wounding currents measured by a Ca^2+^-specific vibrating probe in the quadruple mutant *Atglr1.2 Atglr1.4 Atglr2.2 Atglr3.3* reached a weaker steady-state flux faster than wild-type (WT) plants (55). Callose sealing of damaged cells likewise was affected in various GLR mutant lines compared to that of the WT, with effects correlated with the number of mutations, up to quadruple mutants, before reaching a comparable level, suggesting that GLR suppression is beneficial for the short-term wounding response (55). This effect was pharmacologically phenocopied in maize, both in severed roots and in the regeneration of recalcitrant calli as both dramatically improved after applying the GLR inhibitor 6-cyano-2,3-dihydroxy-7-nitroquinoxaline (CNQX) (55) (see Section 4.2.2). The fast and dynamic nature of these changes in GLR expression underscores the biological significance represented by the multiplicity of genes and division of clades. In this specific case, waves of GLR repression and induction could further be associated with GLRs playing opposing roles in the balance between regeneration and defense (55).

In a separate study, genes encoding *At*GLR2.7, *At*GLR2.8, and *At*GLR2.9 were shown to be a part of a general stress response upregulated by immune elicitors such as peptides flagellin 22 (flg22), elf18, and Pep1 (7). The triple knockout of these genes led to lower [Ca^2+^]_*cyt*_, phenocopying a *bak1*-*5* mutant (7). BRASSINOSTEROID INSENSITIVE 1-ASSOCIATED KINASE 1 (BAK1) is a key signaling kinase in the early immune response, and, while it has yet to be shown to directly interact with a GLR, its signaling pathways have been found to intersect with signaling that is dependent on GLRs and the *A. thaliana* tandem-pore channel 1 (*At*TPC1) vacuolar Ca^2+^ channel under aphid attack (151). This association may further reveal that GLRs and TPC1 cooperatively regulate [Ca^2+^]_*cyt*_ dynamics. In studies of small radish and cotton plants, *RsGluR* and *GhGLR4.8* have been found to confer resistance to pathogens, suggesting a conserved role of GLRs in plant–pathogen interactions (71, 89). Both wounding and pathogen resistance require fast signaling that could fit the properties of GLRs, and both reveal unexpected and complex GLR gene regulation patterns, implying the existence of feedback mechanisms and the need for specific GLR family members to be present spatially and temporally. Moreover, adult Venus flytrap plants that are excitable by touch show a robust increase in *DmGLR3.6* RNA relative to the nonexcitable juvenile plants as well as an enriched expression of *DmGLR3.6* RNA compared to other membrane transporters (129). Computational approaches have further suggested that expression patterns of specific *At*GLRs are associated with stress responses at various levels, including a predicted link to the stress G-protein-coupled γ-aminobutyric acid (GABA) signaling pathway (124).

### 3.2. Plant Glutamate Receptors’ Impact on Gene Expression, Near and Far

Consequential to the elicited GLR-dependent signals are reported transcriptional regulations and the identification of hormonal signaling networks also associated with GLRs. The most dramatic published example regards the reproduction of the moss *P. patens* when its only two GLR genes are mutated. The *Ppglr1 Ppglr2* knockout abrogates the sperm chemotaxis reaction and further produces profound transcriptomic alteration on the gametophores, including the suppression of the transcription factor BELL1, which is essential for postmeiotic embryo development, rendering the double mutant sterile (115).

Prominent regulations in angiosperms include the association to genes encoding members of the JASMONATE ZIM DOMAIN (JAZ) family, reputable marker proteins for activity in the jasmonic acid signaling pathway that is critical to defense signaling. *JAZ* expression and jasmonate biosynthesis are typically upregulated in WT plants upon tissue wounding but are not activated at distal leaves away from the damage site in plants without *At*GLR3.3 and *At*GLR3.6, as well as homologs *Os*GLR3.4 and *Sl*GLR3.5 from rice and tomato, respectively (107, 135, 156, 169). *JAZ* expression at the wounding site showed no significant differences with or without GLRs (107). The difference in GLR-dependent *JAZ* expression near and far supports its involvement in a long-distance defense-signaling mechanism oriented to cell–cell propagation through the undamaged tissue, and less so at the damage site (36, 107, 127). In apparent contradiction are root-wounding experiments where local transcriptional effects are immediate, as captured in *Arabidopsis* by fast ATAC-seq chromatin-opening techniques (55). Here and in other instances, GLR activation shows clear local responses creating a mechanistic chasm between long-distance signaling and the role of GLRs in local signaling (151), suggesting the existence of precise sensing mechanisms that actuate in GLR transcriptional modulation. Through examination of the hormonal pathways exploited, this apparent ambivalence between signaling near and far can be summarized by a trade-off between defense and postwounding tissue regeneration. Severing roots is expected to initiate long-distance signals through the shoots and upregulate *JAZ* and *GLR* expression (135, 156), and it also appears to favor defense since it counters tissue regeneration by preventing the formation of calli and limiting cell division rates (55). Crucially, GLR activity induced salicylic acid (SA) pathway–related genes at damage sites, supporting previous connections of *At*GLR3.3 to the SA pathway for immunity (40, 55, 87). To our knowledge, there is no evidence of SA pathway upregulation at distal sites directly activated by wound-induced long-distance signaling. However, SA is known to be a key factor in systemic acquired resistance following infection, such that SA levels accumulate at the infection site and distal leaves (23, 68).

In roots of WT *Arabidopsis*, the destruction of single cells by two-photon laser ablation failed to produce evidence of jasmonic acid accumulation in roots or photosynthetic organs (98). Similarly, unlike damage via root cap decapitation, single-cell damage did not increase SA in roots, illustrating a quantifiable difference between single-cell damage and mass destruction of large cell populations when crushed or cut (55, 98). It is intriguing that *JAZ* transcript levels similarly showed a difference between mild caterpillar feeding and leaf cutting (128). Taken together, the available data seem to define a forthcoming challenge in identifying detection mechanisms that sense the magnitude of damage and dose-dependent responses. If such a mechanism exists, there is a good chance that GLRs should be involved as effectors, but perhaps also as sensors. In that regard, it should be noted that various clade 3 members possess a cognate nuclear localization signal (NLS) in the C terminus (165). Reminiscent of ETHYLENE-INSENSITIVE 2 (EIN2), which is involved in ethylene sensing, it is tempting to consider that the proteolytic cleavage of C-terminal fragments adjacent to the NLS of certain GLRs could act as a putative transcriptional signal (159, 164). In addition to jasmonic acid and SA, GLR activity has been connected to the hormone abscisic acid such that its biosynthesis is negatively regulated by *At*GLR1.1 during seed germination (70, 76).

## 4. INSIGHTS INTO SIGNALING FROM PLANT GLUTAMATE RECEPTOR MOLECULAR PROPERTIES

GLR participation in a vast breadth of physiological roles facing different environmental stresses—both biotic and abiotic—could suggest an array of molecular mechanisms governing channel properties. Rapidly improving biophysical and biochemical techniques, which include heterologous expression for electrophysiology and protein purification to detail GLRs’ structure function, have begun to unravel their molecular detail. The combination of experimental methods now affords the opportunity to critically assess the compatibility between the genetic studies, physiological assays, and protein biochemistry needed to hypothesize necessary conditions for the functional definition.

### 4.1. Oligomerization

The predominant architectural difference demarcating *At*GLR3.4 from iGluRs is the non-domain-swapped configuration of the extracellular domains (Figure 2). The pairing of dimers is the same at both the LBD and ATD layers. In AMPARs, NMDARs, and KARs, there is a distinct swapped configuration as the dimer pairings switch between the LBD and ATD (Figure 2). This structural feature is relevant because domain swapping underpins the interactions between subunits of a tetramer, namely by impacting the way force is transmitted from the movement of one subunit of the tetramer to another subunit upon ligand binding and undergoing conformational changes. Of the mammalian iGluRs, only the δ-receptors, the least understood of the four families of iGluRs, have been reported to adopt a nonswapped configuration (11, 12) (Figure 2). Phylogenetic analysis of full-length sequences of glutamate receptors provocatively suggests that GLRs’ most closely related homologs may be the δ-receptors (21). This comparison further insinuates that GLRs and δ-receptors may share other functional properties. However, this hypothesis has yet to be experimentally validated. Recent breakthroughs elevated δ-receptors from the formerly used moniker of orphan receptors because of their insensitivity to glycine and d-serine. Studies of δ-receptors’ structural and electrophysical properties recently showed that these channels are gated when they coassemble with auxiliary subunits (12, 16). Before these discoveries, a disease variant harboring a mutation in the M3 domain produced a constitutively open ion channel, allowing a current without exogenous amino acids (173). It is unclear if the protein was completely free of any activating ligand, but it was shown to be activated by contamination levels of amino acids (73). These results are in some aspects reminiscent of GLR activity (see Section 4.2.4).

Despite being tetrameric receptor proteins, mammalian iGluR subunits are gated independently, lending heteromerization the ability to tune the gating response in a stoichiometrically dependent manner (46, 167). AMPARs and KARs are known to exist as homotetramers but are predominantly expressed as diheterotetramers in the central nervous system (94, 145). The relevance of heteromerization on function is well illustrated in the NMDAR family. NMDARs are obligate heterotetramers and form diheteromers and triheteromers, conferring dramatic differences in open-channel probability, single-channel conductance, EC_50_ coefficients for glutamate, and deactivation kinetics (51, 93, 96). In terms of ion selectivity, heteromers with a GluN3 subunit show strongly attenuated Ca^2+^ permeability and are involved in selective neuronal inhibition—marking a dramatic shift from the typical excitatory action of iGluRs (116).

The resolved structure of *At*GLR3.4 is that of a homotetramer, supporting the notion that all GLRs are likely to make functional channels only as tetramers and indicating that a homomeric existence is thermodynamically possible. However, single-cell sampling from *Arabidopsis* leaf tissue indicates that at least five GLRs may be coexpressed in the same cell (125). In roots, individual cells express between 5 and 12 different GLRs (55). A collection of experimental techniques, including yeast two-hybrid screens and Förster-resonance energy transfer (FRET) analysis in HEK293 cells, already suggests that heteromerization is a major factor for GLR function (119, 153). Still uncertain are the naturally occurring heteromeric combinations, as discernable patterns of expression are not yet possible and will depend on environmental factors. *AtGLR3.7* transcripts seem to be the only ones ubiquitously expressed in all leaf samples, suggesting that *At*GLR3.7 plays a central role in physiology, possibly through heteromerization (125).

### 4.2. Amino-Terminal Domain and Ligand-Binding Domain: Sensing the Outer Space

Chemical cell–cell communication requires the existence of extracellular sensors to bind ligands. The ATD and LBD perform these functions in glutamate receptors. Sequences encoding the ATD and LBD are highly divergent between mammalian iGluRs and plant GLRs but, paradoxically, their mechanisms of action seem remarkably well conserved at the structural level.

#### 4.2.1. Modulation of the amino-terminal domain: sensing redox potential?

The ATD is the largest domain of plant GLRs, consisting of approximately 45% of the whole protein. The identification of a glutathione (GSH)-binding site in the ATD by cryo-EM is arguably the first time a cognate receptor has been described in the ATD of any plant GLR. The binding of GSH to the ATD of *At*GLR3.4 through S-glutathionylation supports the general role of GSH observed from *At*GLR3.3-dependent depolarization of *Arabidopsis* roots and [Ca^2+^]_*cyt*_ elevation in root and leaf tissues (45, 87, 120). Judging from sequence alignments, *At*GLR3.3 lacks the GSH-binding site identified in *At*GLR3.4; therefore, GSH may find multiple mechanistic actions, or heteromerization of *At*GLR3.3 and *At*GLR3.4 could contribute to the GSH response reported in roots and leaves (153). The discovery of the effect of GSH on GLRs illustrates the power of structural descriptions in generating testable physiological hypotheses. This was an unforeseeable observation and has potentially larger ramifications. Environmental conditions that modulate GSH concentrations, or redox potential in general, may in turn regulate GLRs. Implications of GSH as a key allosteric modulator for GLRs would be expected to impact not only Ca^2+^ signaling but also ROS signaling. GSH is a potent antioxidant and is chemically stable under conditions of low oxidative stress. Under high oxidative stress, GSH acts as a ROS scavenger to minimize the toxicity of free radicals and catalyzes glutathione peroxidase detoxification of hydrogen peroxide. In both cases, electron donation buffers ROS concentrations and results in the formation of oxidized GSH (GSSG) as a byproduct that was shown to induce a fraction of the GSH response (120). While plasma membrane–localized GLRs garner the most attention, the apoplastic space is a more oxidized environment with low antioxidant accumulations, and the majority of ROS buffering takes place intracellularly (114). This difference may suggest a functional difference between GLRs based on localization to the plasma membrane or endomembranes. The role of the ATD and GSH binding similarly presents a potential molecular bridge between GLRs and reported immunity phenotypes (87). Provocatively, in the fungal resistant *Ghglr4.8* mutant, only a single-nucleotide polymorphism resulting in an amino acid substitution from leucine to isoleucine at position 150 (I150L) found in the ATD was responsible for the fungal resistance (89). If fungal resistance is also related to GSH signaling or the ATD is subjected to broader allosteric modulation is yet to be understood.

#### 4.2.2. Amino acid stimulation of plant glutamate receptors.

Exogenous amino acid application is a general stimulator to potentiate GLR-mediated currents as well as to increase [Ca^2+^]_*cyt*_ and induce membrane depolarizations in planta (120, 137, 140). By comparison to iGluRs, glutamatergic signaling in plants lacks the unparalleled specificity observed in neurons. Instead, glutamate bolsters plant development through far more general roles, including nitrogen metabolism, chlorophyll biosynthesis, and the potential regulation of over 100 genes (17, 49, 69, 121, 154, 155). Estimations of homeostatic glutamate and other amino acid concentrations fall into the millimolar range, orders of magnitude above the measured EC_50_ of GLRs (39, 59, 91). In some instances, apoplastic glutamate reaching 50 mM and 100 mM at wounding sites was wrongly suggested to be needed for signaling based on the intensity-based glutamate-sensing fluorescent reporter (iGluSnFR), which saturates between 1 and 10 mM of glutamate (101, 135, 146). At least a dozen amino acids are considered GLR agonists by functional assays: glutamate, glycine, d-serine, l-serine, asparagine, threonine, alanine, cysteine, methionine, histidine, tryptophan, phenylalanine, leucine, tyrosine, and the nonproteinogenic amino acid 1-aminocyclopropane-1-carboxylic acid (ACC) (2, 40, 75, 106, 120, 137, 140, 152). While the full list of agonists for the 20 *Arabidopsis* GLRs remains to be determined, the available data already warrant the existence of full to partial agonists, yielding a spectrum of ligand (amino acid and GSH) efficacy. Two-electrode voltage clamp recordings from *Xenopus* oocytes expressing *At*GLR1.4 show an activation for seven large hydrophobic amino acids, with methionine evoking the strongest activation. Tyrosine, asparagine, and threonine potentiated current only to 20% of the maximum activation reported by methionine (140).

Additional considerations should be given to the effect of antagonists. The iGluR antagonists CNQX; D-2-amino-5-phosphonopentanoate (D-AP5); and 6,7-dinitroquinoxaline-2,3-dione (DNQX) have been found to inhibit GLR channel activity (104, 115, 140). Against *Pp*GLR1 activity—which is closely related to clade 3 *At*GLRs—aspartate is inhibitory (106). Challenging *At*GLR1.4, nine other proteinogenic amino acids with no excitatory capability provide a degree of antagonism (140). *At*GLR1.4’s predicted ligand profile provides one of the few tantalizing distinctions explaining clade divergence. Common agonists of clade 3 GLRs include cysteine, alanine, glutamate, and glycine (2), which serve as antagonists or induce little effect (Cys>Ala>>Glu ≥ Gly) on *At*GLR1.4. The overall nondiscriminatory ligand-gating profile of GLRs more closely resembles PBPs, prokaryotic GluR0, and *Av*GluR1 than mammalian iGluRs in the central nervous system (18, 92), further pointing toward a primitive amino acid–binding capacity divorced from glutamate specificity.

ACC differentiates itself from other amino acids targeting the LBD because it is a nonproteinogenic amino acid. Besides being a partial agonist at the human NMDAR glycine/d-serine site (60, 108, 109), ACC in plants is a precursor in the synthesis of the central plant hormone ethylene (66). Genetic dissection of ACC signaling revealed that mutant *Arabidopsis* plants with an octuple knockout of all ACC synthase genes displayed reduced seed set because of impaired pollen tube attraction (106). Challenged against *Pp*GLR1 during heterologous expression, ACC largely outperformed other amino acids in inducing [Ca^2+^]_*cyt*_ elevations (106). ACC in angiosperms evoked currents from root protoplasts and caused transient Ca^2+^ responses in the ovule (106). The effectiveness of ACC inspires the question of whether the most physiologically relevant ligands exist in a new class beyond the standard 20 amino acids.

One possible hypothesis for broad amino acid regulation postulates a role for an amino acid sensor that monitors metabolic status or environmental cues (40). Intriguingly, ACC and GSH are similarly stable molecules under reduced conditions with low oxidative stress, which may hint at roles in sensing the oxidative status of the environment. A molecular mechanism that signals the oxidative environment may point to potential roles in tolerance to environmental stresses—including drought, salinity, and alternating light/dark regimes known to modify ROS—or within tissues such as the style and transmitting tract. The alternative hypothesis is that a GLR–ligand interaction has specificity in physiological function (8). Possibilities include a preference for methionine-induced activation in stomata, for ACC or d-serine in reproduction, or for glutamate in wound signaling (75, 106, 146).

#### 4.2.3. Ligand-binding coordination: designed for amino acid sensing?

**Figure 4:**
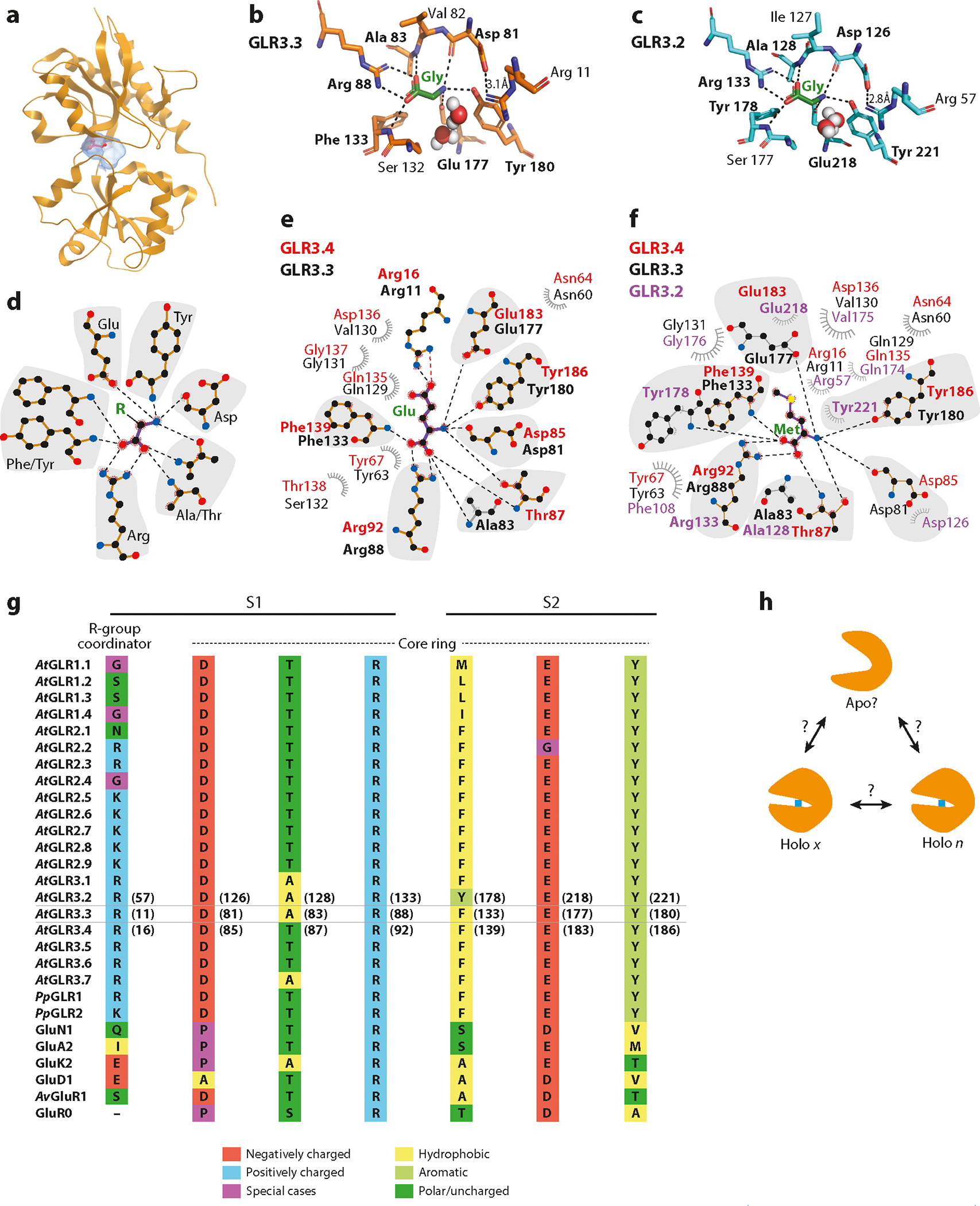
LBD structure and equivalence of ligand coordination by *At*GLR3.2, *At*GLR3.3, and *At*GLR3.4. (*a*) Representation of a closed clamshell-like LBD from GLR3.3 in complex with glycine; the blue mesh represents the volumetric space of the amino acid–binding pocket. (*b*) Close-up ligand-binding interactions between *At*GLR3.3 and glycine (PDB ID 6R88). (*c*) Close-up ligand-binding interactions between *At*GLR3.2 and glycine (PDB ID 6VEA). (*d*) 2D diagram of the consensus motif Asp-Ala/Thr-Arg-Phe/Tyr-Glu-Tyr that coordinates amino acid carboxyl and amino groups. (*e*,*f*) 2D diagram of equivalent ligand–protein interactions for different GLR LBDs coordinating glutamate (*e*) or methionine (*f*). Residue labels are color coordinated with the protein label colors shown at left. Shadows encircle residues of the consensus motif, and nonshaded residues coordinate the amino acid side chain. All annotation numbers follow original publications. For panels *b*–*f*, hydrogen bonding is shown as dashed lines with residues in bold, and nonbonded interactions are depicted as eyelashes. 2D diagrams were drawn with LigPlotPlus with PDB accession codes 7LZ0, 7LZ2, 6R85, 6R8A, and 6VE8. Maps are not drawn to scale. (*g*) Sequence alignment of the core amino acid binding residues. (*h*) Model of unknown mechanisms for ligand binding. Holo *x* represents the conformation of any ligand (*x*) bound, and holo *n* represents the ligand-bound conformation for any number (*n*) of ligands. Abbreviations: Ala, alanine; Arg, arginine; Asn, asparagine; Asp, aspartate; Gln, glutamine; GLR, glutamate receptor-like; Glu, glutamate; Gly, glycine; Ile, isoleucine; LBD, ligand-binding domain; PDB, Protein Data Bank; PDB ID, Protein Data Bank identification; Phe, phenylalanine; Ser, serine; Thr, threonine; Tyr, tyrosine; Val, valine; 2D, two-dimensional.

Although there is a long list of GLR amino acid agonists that have been determined functionally, only a subset has been biochemically determined to bind to the LBD of a specific GLR. The *At*GLR3.3 LBD was crystallized in complex with glycine, glutamate, cysteine, and methionine (2). The *At*GLR3.2 LBD was crystallized in complex with glycine and methionine (42). Isolated *At*GLR3.4 LBDs were also crystallized in complex with glutamate and methionine, as well as l-serine (45). All three LBDs show equivalent ligand coordination for each amino acid such that a consensus motif of Asp-Ala/Thr-Arg-Phe/Tyr-Glu-Tyr coordinates the carboxyl and amino group of amino acids (Figure 4). An arginine found among all clade 3 channels—yet absent in iGluRs—provides a key interaction, stabilizing the amino acid side chain (Figure 4). The strong carboxyl and amino coordination has been proposed to be a factor underpinning the apparent nondiscriminatory amino acid binding that allows the computational prediction of amino acid docking, such as ACC, or in vitro binding of d-serine (2, 47). The observation that the binding pocket size can be adjusted by packing water molecules suggests that additional interactions accommodate different R-group lengths (2, 42).

#### 4.2.4. A noncanonical ligand-gating model for plant glutamate receptors?

Structure determination of isolated GLR LBDs indicates a closed clamshell-like LBD structure when bound to an amino acid resembling that of the iGluR that is known to conduct ions. However, there are various challenges to ligand gating in the plant cell, and the available electrophysiological data need to be fitted with this interpretation. In electrophysiological recordings, amino acid concentrations in the micromolar to millimolar range are necessary to potentiate currents, but a constitutively active background current is present without exogenous ligands in many cases. These results are evident in whole-cell patch clamp during heterologous expression, but other more physiological systems, such as protoplasts from *Arabidopsis* roots or *P. patens* protonemata, behave similarly (106, 115). The binding affinity (*K*_d_) for several amino acids to *At*GLR3.3 was determined to be in the low to submicromolar range (ranging from 0.33 μM to 5.5 μM for cysteine, methionine, glutamate, alanine, asparagine, l-serine, and glycine, as determined by thermophoresis), which is significantly lower than the physiological amino acid concentrations in the millimolar range found in the plant cell apoplast (2), and the same sort of values were found for *At*GLR3.4 (48). This discrepancy alone should warrant the careful scrutiny of data where GLR-mediated phenomena are described for elicitation with 100-mM glutamate in roots or wounded leaves (135, 146), and this explanation is more than reasonable if applied to isolated mammalian cells, such as HEK (135).

In light of the high-binding affinities for amino acids and the possible millimolar range of concentrations for many amino acids in the apoplast (and mammalian culture media), it is a valid hypothesis that the LBD is constitutively occupied by contamination. The constitutive occupancy can be predicted to force GLR LBD closure and favor a stochastic ion channel opening, jeopardizing the physiological relevance of the dynamic or fast amino acid gating that is quintessential of iGluRs. If one can always expect an amino acid to be bound to the receptor, one can speculate that amino acid binding serves as a structurally integral component of the receptor. Analysis of the ligand-binding pocket also reveals that two residues coordinating the ligand—including the plant-specific arginine (Arg11 in *At*GLR3.3; Arg57 in *At*GLR3.2)—may also be able to form salt bridges favorable to the closed clamshell configuration, possibly locking the holo (or ligand-bound) configuration. The inability to obtain an apo (or ligand-free) state suggests conformational instability when an amino acid is absent.

iGluRs and GLRs may be conjectured to share unknown mechanisms for conducting a steady-state current, leading to the reimagining of the conventional relationships between ligand gating of glutamate receptors and ion signaling for a more nuanced scheme. Channel activation by contaminating amino acids was previously proposed from electrophysiology experiments on AMPARs carrying a mutation copying the neurodegenerative lurcher mutant that confers constitutively active currents (73). As informative as *K*_d_ measures are, computational methods calculating the off-rates of ligand unbinding may also be useful to address questions on ligand binding. Simulations of iGluRs suggest ligand binding/unbinding pathways and kinetics are key in the evolutionary adaption to fast signaling (168). However, under what conditions an apo configuration may be adopted or if there is a role for ligand substitutions in the binding pocket, given the spectrum of agonists and antagonists (Figure 4h), is not known. Noteworthy experiments using whole-cell patch clamp by Vincill et al. (152) and two-electrode voltage clamp by Tapken et al. (140) observed no clear and obvious desensitization after amino acid potentiation (140,152). Only sequential exposures of amino acids, separated by 2 min, to roots resulted in diminishing membrane depolarizations upon the subsequent stimulation (137), an observation that may find an explanation in completely different mechanisms, namely ion store adaptation. The concept of desensitization is well defined by proposed molecular mechanics (103) and a defined temporal scale on the order of milliseconds, not minutes, none of which are applicable to what has been observed in roots or GLRs under heterologous expression. A still-undetermined ligand-gating scheme needs to resolve the triple discrepancy between biochemically calculated amino acid binding affinities (submicromolar), the dose response of electrophysiological patch clamp recordings (micromolar to millimolar), and the nature of the physiological amino acids both in specificity and free concentration (millimolar to tens of millimolar).

An additional consideration for reimagining ligand-gating relationships comes from protein–protein interactions. AMPARs are typically characterized by a fast desensitization process leading to the occlusion of the pore (147). Yet, recent years have witnessed reports illustrating a greater degree of superactivation: a repotentiation of ion flux with prolonged ligand application requiring transmembrane auxiliary proteins (TARPs), such as STARGAZINS (15, 123). Further demonstrations show that AMPARs without auxiliary proteins may conduct ions when desensitized, and the conductance is greatly enhanced by TARP co-expression (15, 24, 123). CNIs are similarly positive regulators of AMPARs, working to maintain an open conductive channel (9, 134, 138). CNIs enhance glutamate sensitivity, slow desensitization rates, enhance a steady-state current following an attenuated desensitization process, and relieve polyamine block by promoting polyamine permeation (10, 25). As a result, an ion signaling model managing a global equilibrium of ion concentrations such as Ca^2+^ between stores and the cytosol in plants could be envisaged where steady-state ion fluxes are fine-tuned on the basis of *K*_d_ acting in concert with ligand availability—depending on GLRs’ subcellular localization to various organelles via the CNIH sorting mechanism.

### 4.3. Integrating Ion Channel Gating for Systemic Signal Propagation and Nonsystemic Signals

GLR participation in both systemic and nonsystemic pathways confronts the necessity to decipher various potential activation and regulatory mechanisms, as well as other interacting proteins. In wound-induced systemic signaling in leaves, GLRs are proposed to operate in the undamaged tissue along the phloem and xylem. Chemical diffusion of glutamate or other amino acids emanating from the damage site as an excitatory stimulus would be unlikely to surpass the rates of electrical signaling, and amino acids have so far not been found to propagate over long distances at all upon wounding (146). Seminal work from Diana Bowles’s lab (161) experimentally supported the conclusion that long-distance signaling is achieved through electric potential as opposed to small ligand/hormone diffusion. Their approach distinguished a phloem-transmissible chemical signal and electrical propagation by microcooling the petioles to slow diffusion when applying a wounding stimulus to a cotyledon and measuring electrical propagations, chemical translocation (reported by ^11^C-labeled photosynthate), and proteinase inhibitor (*PIN*) transcripts in the roots. Systemic *PIN* activity was correlated to electrical responses, while diffusion/translocation could be impaired and leave *PIN* activity unaffected (161). Translating these conclusions to GLR research logically demonstrates that GLR-mediated slow electrical potential is associated with the Ca^2+^ waves induced by 100-mM glutamate. Whether observed Ca^2+^ waves result from other chemical elicitors besides 100-mM glutamate remains to be shown. More surprisingly, a pH effect was found on HEK mammalian cells exposed to 100-mM glutamate and attributed to GLRs’ function in Ca^2+^ transport (135). Applying 100-mM glutamate to isolated cells is hardly compatible with any evidence about GLR binding affinity, gating, or function, while pH has long been known to affect Ca^2+^ transport by various different effects (160). Because of the basal, or steady-state, concentrations of apoplastic amino acids, it has been hypothesized that a coincidence-detecting mechanism is employed where ligand binding and membrane voltage or another stimulus collectively activate GLRs. Genetic manipulation of other membrane transporters, such as AHA1, MSL10, and TPC1, that are signaling partners for wound responses (at least in the case of AHA1 and MSL10) is expected to modulate the membrane potential and suggests that a depolarized potential enhances GLR activity (79, 105). However, the underlying mechanism allowing ion channels to communicate requires a much deeper level of understanding of ion channel gating (including channel activation, inactivation, and deactivation), as well as ion selectivity. To interpret GLR behavior from heterologous expression, several considerations must be made, chiefly the limitations of commanding the membrane potential. In patch clamp or two-electrode voltage clamp electrophysiology, the animal cell plasma membrane is only capable of enduring voltage pulses of −140 or −150 mV—just approximating the resting voltages a channel would normally be exposed to in the plant cell plasma membrane. Published literature commonly explores voltages from −100 mV to 0 mV or even more depolarized voltages (115, 140, 152, 166). Robust activations of GLRs in heterologous expression at these potentials suggest that a depolarization-activated current is plausible.

Nonsystemic signaling, however, may require a revised model. For example, during aphid feeding—which precisely attacks the phloem network and is thought to more closely resemble pattern perception regulation—GLRs are involved in local Ca^2+^ signaling, presumably within single cells represented by slower Ca^2+^ wave rates of 6 μm/s, at aphid feeding sites (151). Pattern perception pathways involving microbe- or pathogen-associated molecular patterns (MAMPs or PAMPs) may elevate amino acid concentrations, suggesting an activation mechanism dependent on elevated concentrations of amino acids (40). Elevated amino acid concentrations may arise from exocytosis—eerily similar to the synaptic model of iGluR activation—although pattern recognition receptor (PRR)-activated exocytosis is not well resolved and requires further experimental support. Appreciating the intermediate steps between PAMP perception and its influence on membrane transport will shed light on this topic. Alternatively, amino acids may be recognized as damage-associated molecular patterns (DAMPs), distinguishing self from nonself glutamate pools that are released from injured cells (146) and build a glutamate gradient. Remarkably, leaf crushing generates Ca^2+^ signals confined to the wounded leaf and is an insufficient stimulus to induce systemic signals. Only supplemental application of glutamate at very high concentrations triggered the long-distance signaling (146). Severing the root, however, was sufficient to stimulate a systemic root-to-shoot signal in both *Arabidopsis* and rice (135, 169).

### 4.4. The Transmembrane Domain

The TMD contains two functional regions classically termed the gate and the pore. Owing to the strong structural similarity between GLRs and iGluRs in the M3 gate-forming helix, the argument can be made that the M3 is also part of the ion permeation pathway for both GLR and iGluR structures (45, 150). The M3 and its linkers to the LBD likely play a key role in transducing conformational changes of extracellular domains to the opening and closing of the pore. Studies in AMPARs have shown that the open channel conformation is formed by a rearrangement of the M3 helixes from the inwardly oriented conical shape to one that splays outward (149).

A significant challenge in GLR research is to fully resolve the TMD and determine the order of the M2 and pore loop harboring the selectivity filter. The pore loop and the selectivity filter remain disordered from the full-length *At*GLR3.4 structure and pose a significant hurdle to future research. Despite the community of structural biologists engaged in elucidating iGluR structures, few structures have resolved the pore or selectivity filter, and each successful attempt required the co-expression of auxiliary subunits (19, 150, 171). Site-directed mutagenesis in the iGluR pore demonstrates the role of the Q/R/N editing site in influencing ion selectivity (14, 58, 147). Genes for AMPARs and KARs exonically encode for a glutamine (Q), but postnatal messenger RNA edits to an arginine (R) comprise the majority of the AMPAR/KAR population (50). The Q-to-R mutation renders these iGluRs Ca^2+^ impermeable, and slight chloride (Cl^−^) permeability is also argued to come from electrostatic forces (14, 80). In NMDARs, an encoded asparagine (N) at the Q/R/N site likewise plays a determining role in Ca^2+^ permeability such that targeted mutagenesis to a glutamine (Q) lowers Ca^2+^ permeability (13). At the pore entry facing the cytosol, a highly conserved negative charge—either from an aspartate in AMPARs or a glutamate in NMDARs—plays a role in either AMPARs’ cation versus anion selectivity, or NMDARs’ divalent permeability (131, 150). Interestingly, NMDAR GluN3 subunits lack the conserved negatively charged residue, and heterotetrameric channels with GluN3 greatly reduce Ca^2+^ permeability (116). Last but not least, it must be noted that NMDARs also harbor several more molecular determinants for Ca^2+^ permeability that exist outside the pore (3).

**Figure 5:**
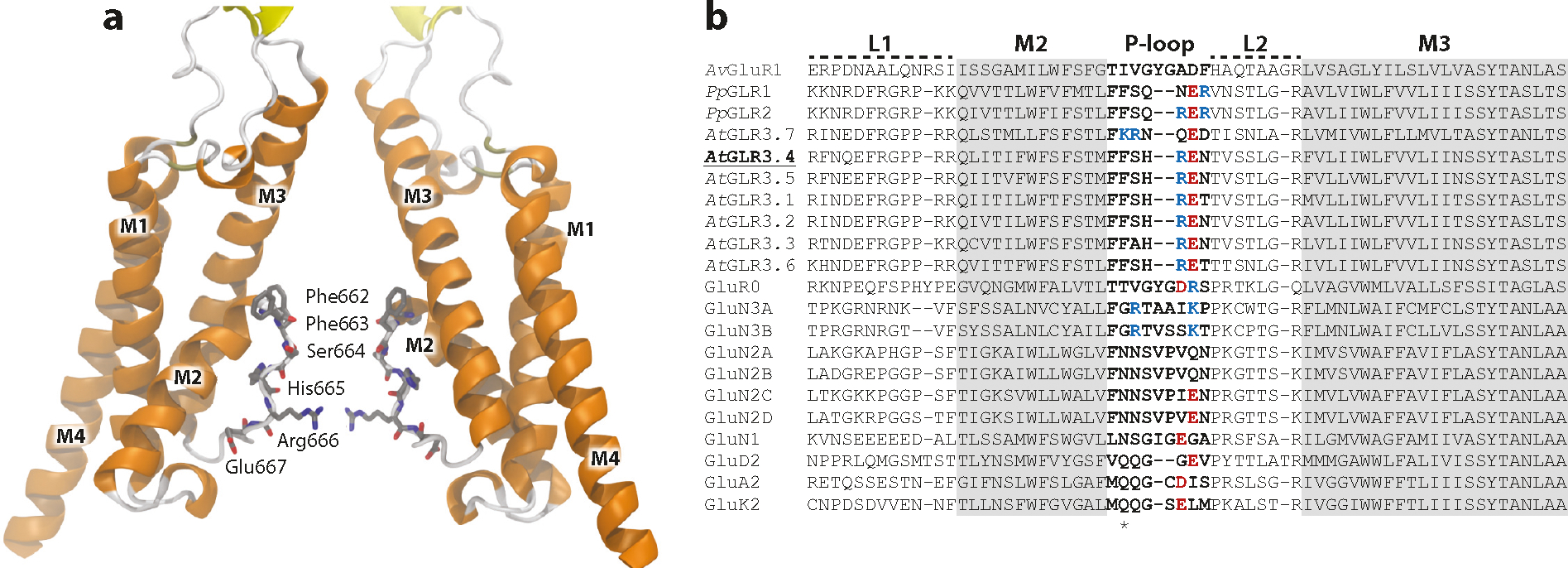
TMD and pore-lining residues of GLRs. (*a*) Model of *At*GLR3.4 TMD and pore-lining residues. α-helices and β-sheets are shown in orange and yellow, respectively. Residues forming the P-loop region are shown in gray in a licorice representation. Only two subunits are shown for clarity. To construct the 3D model of *At*GLR3.4, AlphaFold (62) was used to model the residues from Pro493 to Arg886, focusing on the TMD. To ensure the quality of the model, 24 cycles were used, and the models obtained were subjected to the relax protocol. (*b*) Sequence alignments generated by MUSCLE of clade 3 *At*GLRs, those in *Physcomitrium patens*, and mammalian iGluRs. The asterisk denotes the pair of phenylalanine guarding the selectivity filter entrance. Abbreviations: Arg, arginine; GLR, glutamate receptor-like; Glu, glutamate; His, histidine; iGluRs, ionotropic glutamate receptors; MUSCLE, multiple sequence comparison by log-expectation; Phe, phenylalanine; Pro, proline; Ser, serine; TMD, transmembrane domain; 3D, three-dimensional.

To glean insight into the properties of the GLR M2 and selectivity filter, we analyzed sequence alignments (27, 141) of GLRs and iGluRs and modeled a tetrameric *At*GLR3.4 with AlphaFold (67) to hypothesize the potential pore-lining residues. A pair of phenylalanine residues (Phe662 and Phe663) present strong hydrophobic and aromatic properties guarding the selectivity filter entrance. Marking the narrowest portion of the selectivity filter is a highly conserved arginine (Arg666) bearing a positive charge into the permeation pathway, making an anionic permeation mechanism plausible (Figure 5). Electrophysiology experiments and molecular dynamics simulations provide essential techniques to challenge this hypothesis and advance future models of ion permeation (110).

## 5. CONCLUSION AND FUTURE DIRECTIONS

This and other recent reviews illustrate the dynamics surrounding the field of GLR research. New phenotypes and functions are attributed to this ion channel family on a regular basis. An appreciation of GLR-associated functions in plant biology has now clearly overcome the slow start imposed by their functional redundancy and the elevated number of copies, which delayed integrated molecular genetics approaches. Aside from this positive trend, it is fair to say that the molecular elucidation of the mechanisms of action of the channels is still lagging behind and far from a satisfactory level of knowledge to support all functions that are assigned to GLRs. By and large, the functional validation of most genetic approaches that have been described makes use of a few common denominators: (*a*) GLRs are mostly plasma membrane channels, gated by glutamate, and (*b*) they conduct Ca^2+^. Reasons for this status quo are rooted in an appropriation of concepts from the much more advanced field of mammalian iGluR biology and frequent adaptations to the experimental methods that are easier to access (e.g., Ca^2+^ imaging). Here, we review numerous aspects in which our current knowledge of GLRs is not sufficient to account for many of their attributed functions. Ligand-gating mechanisms, ion selectivity, oligomerization, and interaction with auxiliary proteins are examples of core structural-function properties with incomplete data sets. Knowledge of all these aspects was crucial for the comprehension of the fundamental role of iGluRs in neurobiology, and a call for a similar effort in the plant field to acquire more of the type of mechanistic knowledge necessary for better and more precise functional screenings is warranted.

**Figure 6:**
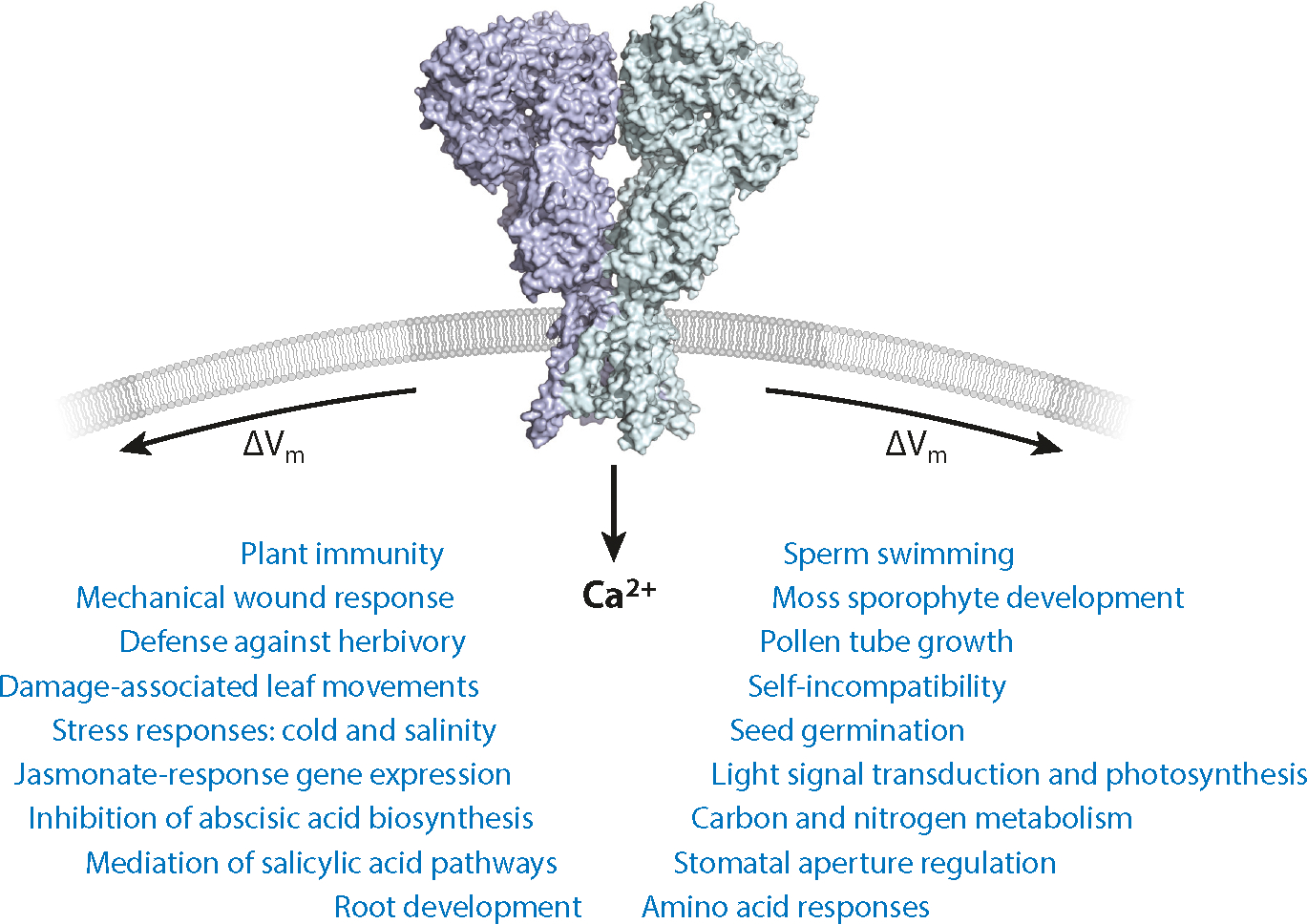
Major physiological processes concerning plant growth and development, reproduction, and defense mechanisms associated with plant glutamate receptors. Physiological phenotypes dependent on GLRs are thus far known to be regulated through electrical signaling by modulating the membrane potential (V_m_) and Ca^2+^ signaling by permitting a Ca^2+^ influx.

Under this prism, the list of future directions will require a communal effort. Atomic structures will be needed to provide insight into the mechanistic basis of ion channel gating and selectivity, offering glimpses of the conformational changes. Ion channel structures are, however, limited in static presentation, and physiological interpretations will require an ensemble approach that couples functional assays and analysis of transgenic GLR mutants. AlphaFold and similar technological advances in structure prediction offer the potential to rapidly generate testable hypotheses to be challenged by functional assays, including electrophysiology. Molecular mechanisms underpinning ion permeation, ligand gating, GLR–CNIH interactions, GLR inactivation and deactivation, putative desensitization, and voltage sensitivity all remain to be determined. New physiological roles wait to be mechanistically linked to GLRs. The latest breakthroughs in genetic screens identifying roles for clade 2 GLRs increase the number of available targets, some of which have been dormant for over 20 years of GLR research. More complete functional descriptions detailing ion selectivity and channel gating made possible by atomic structures, which shed light on molecular properties, are not yet realized. The functional consequences of the ion channel gating mechanism and its contribution to cell signaling, which depends on GLR subunit identity, are almost completely unknown. Mammalian iGluRs are, for example, predominantly excitatory, but some isoforms are involved in selective neuronal inhibition necessary for long-term desensitization and memory (50). The quest to understand plant GLR clade divergence remains unsolved. The study of GLRs so far has relied on *Arabidopsis* and a few other plant species. A comprehension of their mechanistic evolution will require the use of early land plants, which offer the advantage of much simpler genetics. If the trends described here are further consolidated to established roles (Figure 6), namely in terms of wounding repair, reproduction, and host–pathogen interaction, then the extension of functional studies to crops and plants with extreme adaptations will be required, raising the prospect of reaping the benefits of this knowledge in agronomical terms.

## Supplementary Material

Supplementary text

## Figures and Tables

**Table 1:** Experimentally described plant glutamate receptor-like ion channels

Protein (gene accession)	Phenotype	Protein expression/localization [Organ: cell (subcellular)]	Ligand [potentiation/inhibition (method, expression system)]	Observed or inferred ion permeability (method, expression system)	Reference(s)
*At*GLR1.1 (At3g04110)	KO: inhibited seed germination KO: enhances ABA biosynthesis KO: disrupted C/N metabolism KO: reduced pollen tube growth rate WT: chromatin accessibility and induced expression after root wounding	Seeds; Pollen tube; Roots	ND	Nonselective, Ca^2+^ permeable (TEVC, *Xenopus* oocytes)	55, 70, 141, 166
*At*GLR1.2 (At5g48400)	KO: decreased fertility; reduced pollen tube growth rate and abnormal morphogenesis; altered [Ca^2+^]*_cyt_* and growth oscillations; decreased tip Ca^2+^ influx in pollen tube KO: cold sensitivity WT: chromatin accessibility and induced expression after root wounding	Pollen; Roots	Potentiation: d-Ser, Gly; Inhibition: CNQX, DNQX, D-AP5 (whole-cell patch clamp, pollen protoplasts and VP of growing pollen tubes)	Ca^2+^ permeable (Ca^2+^-P and YC3.6 imaging, pollen tubes)	55, 104, 166, 172
*At*GLR1.3 (At5g48410)	KO: decreased cold sensitivity, jasmonate signaling OE: enhanced cold tolerance WT: chromatin accessibility and induced expression after root wounding	Roots	ND	ND	55, 166, 172
*At*GLR1.4 (At3g07520)	KO: decreased Met-induced membrane depolarization in intact cotyledons KO: decreased pollen tube growth rate WT: chromatin accessibility and induced expression after root wounding	Leaves: mesophyll cells (plasma membrane); Roots	Potentiation: Met, Trp, Phe, Leu, Tyr, Asn, Thr; Inhibition: CNQX, DNQX, Arg, Gln, Lys, Val, Iso, His, Cys, Ala, Ser (TEVC, *Xenopus* oocytes)	Nonselective cationic, Ca^2+^ permeable (TEVC, *Xenopus* oocytes)	55, 140, 166
*At*GLR2.1 (At5g27100)	KO: decreased pollen tube growth rate and tip Ca^2+^ influx WT: chromatin accessibility and induced expression after root wounding	Pollen and pollen tubes (tonoplast)	ND	Ca^2+^ (Ca^2+^-VP, pollen tubes)	55, 166
*At*GLR2.2 (At2g24720)	KO: decreased pollen tube growth rate WT: chromatin accessibility and induced expression after root wounding	Pollen tubes; Roots	ND	ND	55, 166
*At*GLR2.3 (At2g24710)	WT: chromatin accessibility and induced expression after root wounding	Roots	ND	ND	55
*At*GLR2.4 (At4g31710)	WT: chromatin accessibility and induced expression after root wounding	Roots	ND	ND	55
*At*GLR2.5 (At5g11210)	WT: chromatin accessibility and induced expression after root wounding	Roots	ND	ND	55
*At*GLR2.6 (At5g11180)	KO: decreased pollen tube growth rate WT: chromatin accessibility after root wounding	Pollen tubes; Roots	ND	ND	55, 166
*At*GLR2.7 (At2g29120)	WT: chromatin accessibility and induced expression after root wounding	Roots	ND	ND	55
*At*GLR2.8 (At2g29110)	WT: chromatin accessibility and induced expression after root wounding	Roots	ND	ND	55
*At*GLR2.9 (At2g29100)	WT: chromatin accessibility and induced expression after root wounding	Roots	ND	ND	55
*At*GLR2.7 *At*GLR2.8 *At*GLR2.9	GLR2.7/2.8/2.9 triple KO: impaired immune responses; reduced [Ca^2+^]*_cyt_* elevations in response to flg22, elf18, and Pep1; susceptibility to Pto infection	Roots	ND	Ca^2+^ (YC3.6 imaging, leaf discs)	7
*At*GLR3.1 (At2g17260)	KO: disrupted herbivory-induced signaling; attenuated [Ca^2+^]*_cyt_* elevations; slower Ca^2+^ and electrical wave propagation; shorter duration membrane potential and [Ca^2+^]*_cyt_* changes WT: chromatin accessibility and induced expression after root wounding OE: impaired Ca^2+^-induced stomata closure	Leaves: xylem contact cells, phloem sieve elements, mesophyll cells (plasma membrane); Roots	Potentiation: Met (whole-cell patch clamp, guard cells)	Ca^2+^ (GCaMP3 imaging, *Arabidopsis* leaves)	22, 55, 75, 111
*At*GLR3.2 (At4g35290)	KO: disrupted herbivory-induced signaling; short duration surface potential changes KO: lateral root primordia overproduction KO: poor plant growth and hypersensitivity to KCl and NaCl under conditions of low extracellular Ca^2+^ WT: chromatin accessibility and induced expression after root wounding	Roots: phloem (sieve plates)	Potentiation: Met, Gly (X-ray crystallography, protein expression in *E. coli*)	Nonselective, Ca^2+^ permeable (whole-cell patch clamp, COS-7 cells; YC3.6 imaging, COS-7 cells)	42, 55, 72, 107, 153, 166
*At*GLR3.3 (At1g42540)	KO: disrupted herbivory- and wound-induced signaling; attenuated [Ca^2+^]*_cyt_* elevation; slowed Ca^2+^ and electrical wave propagations; shorter duration membrane potential cyt Ca^2+^ changes; reduced jasmonate-response gene expression KO: impaired immunity; susceptibility to pathogens and oligogalacturonides; attenuated [Ca^2+^]*_cyt_* produced by GSH KO: abolished amino acid–elicited [Ca^2+^]*_cyt_* signals KO: reduced pollen tube growth rate WT: induced expression after root wounding	Leaves: phloem sieve elements (sieve plate); Roots: ubiquitous expression; Sperm cell membranes	Potentiation: Cys^1,2,4^, Glu^1,2,4^, Gly^1,2,4^, Met^1,2^, Ala^2,4^, L-Ser^2^, Asn^4^, GSH^4,5^, GSSG^4,5^, D-Ser^3^; Inhibition: D-AP5^5^, DNQX^5^	Nonselective, Ca^2+^ permeable (whole-cell patch clamp, COS-7 cells; GCaMP3 imaging, *Arabidopsis* leaves; NES-YC3.6 imaging, *Arabidopsis* root tip cells)	2, 55, 87, 97, 107, 111, 120, 127, 137, 146, 151, 153, 166
*At*GLR3.4 (At1g05200)	KO: lateral root primordia overproduction KO: reduced Ca^2+^ fluxes in growing pollen tube WT: induced expression after root wounding	Roots: phloem (sieve plates); Leaves: epidermal cells (plasma membrane), leaf extract (chloroplast membrane, plasma membrane); Onion epidermal cell (plasma membrane); Pollen tube tip (plasma membrane)	Potentiation: Asn, L-Ser, Gly, Glu (whole-cell patch clamp, HEK293 cells); Potentiation: Glu, GSH (cryo-EM, whole-cell patch clamp, COS-7 cells)	Nonselective, Ca^2+^ permeable (whole-cell patch clamp, COS-7 and HEK293 cells)	45, 55, 102, 144, 152
*At*GLR3.5^6^ (At2g32390)	KO: altered wound-induced electrical signaling; attenuated peak amplitude of membrane depolarization; generation of a new membrane depolarization in leaves not neighboring the wound site KO: reduced seed germination; loss of GLR3.5 enhances ABA biosynthesis KO: early senescence and abnormal organ morphology KO: self-incompatibility: decreased Ca^2+^ elevations in papilla protoplasts WT: reduced expression after root wounding	Leaves (inner mitochondrial membrane, chloroplast membrane); Leaves: leaf mesophyll cells (plasma membrane); Roots	Potentiation: Met (whole-cell patch clamp, guard cells)	Ca^2+^ (YC3.6 imaging, papilla cell protoplasts)	55, 62, 75, 76, 127, 143
*At*GLR3.6 (At3g51480)	KO: disrupted herbivory- and wound-induced signaling; attenuated [Ca^2+^]*_cyt_* elevations; slowed Ca^2+^ and electrical propagations; short duration membrane potential [Ca^2+^]*_cyt_* changes; reduced jasmonate-response gene expression KO: stunted root development WT: reduced expression after root wounding	Leaves: xylem contact cells	Potentiation: Glu	Ca^2+^ (GCaMP3 imaging, *Arabidopsis*)	55, 107, 111, 136, 146
*At*GLR3.7 (At2g32400)	KO: self-incompatibility; decreased Ca^2+^ elevations in papilla protoplasts KO: increased sensitivity to salt stress WT: reduced expression after root wounding	Roots and leaves: ubiquitous mRNA expression	ND	Ca^2+^ (YC3.6 imaging, papilla cell protoplasts)	55, 62, 125, 157
*Arabidopsis*: Triple 1.2/1.4/3.3 Quadruple 3.1/3.2/3.3/3.6 Quadruple 1.2/1.4/2.2/3.3	Triple and quad KOs: increased regeneration frequency after root cap wounding; faster recovery of Ca^2+^ wounding currents; faster callose accumulation during the wounding response; signaling through salicylic acid on regeneration	Roots (calli)	Inhibition: CNQX (Ca^2+^-VP, root wounds)	Ca^2+^ (Ca^2+^-VP, root wounds)	55
*Arabidopsis*: Double 3.3/3.6 Quadruple 3.1/3.2/3.3/3.6	Double and quad KOs: insensitive to ACC elicitation of currents	Roots	Potentiation: ACC (whole-cell patch clamp, root protoplasts)	ND	106
*Arabidopsis*: Double 3.3/3.6	Double KO: abolished herbivory- and wound-induced membrane depolarization and [Ca^2+^]*_cyt_* elevations; reduced jasmonate-response gene expression; decreased [Ca^2+^]*_cyt_* elevations elicited around aphid-feeding sites; abolished wound-induced leaf movements	Leaves	ND	Ca^2+^ (GCaMP3 imaging, *Arabidopsis*)	82, 111, 151
*Arabidopsis*: Double 3.1/3.3	Double KO: weak herbivory-indueed membrane depolarization and [Ca^2+^]*_cyt_* elevations	Leaves	ND	Ca^2+^ (GCaMP3 imaging, *Arabidopsis*)	111
*Arabidopsis*: Double 3.1/3.5	Double KO: impaired Ca^2+^-induced stomata closure	Leaves: stomata	Potentiation: Met (whole-cell patch clamp, guard cells)	Ca^2+^ (aequorin imaging, *Arabidopsis* seedlings)	75
*Pp*GLR1 (Pp3c12_5540V3.1)	KO: impaired reproductive fitness; reduced number of sporophytes and poor sporophyte maturation, potential deregulation of BELL1 transcriptional factor	Vegetative organs and reproductive organs	Potentiation: ACC, Glu, Gly; Weak agonists: His, D-Ser; Inhibitors: Asp, D-AP5, CNQX (YC3.6 imaging, COS-7 cells)	Nonselective, Ca^2+^ permeable (whole-cell patch clamp, COS-7 cells and moss protonema protoplasts)	106, 115
*Pp*GLR2 (Pp3c15_25650V3.1)	KO: strongly impaired reproduction; few sporophytes; poor sporophyte maturation	Reproductive organs	ND	ND	115
*Physcomitrium patens* Double ½	Double KO: sterile; spore production greatly reduced; sporophyte immaturity; loss of sperm chemotaxis response; suppression of BELL1 transcriptional factor and block of diploid to haploid transition	Vegetative organs and reproductive organs	Inhibitors: D-AP5, CNQX (whole-cell patch clamp, COS-7 cells and moss protonema protoplasts)	Nonselective, Ca^2+^ permeable (whole-cell patch clamp, moss protonema protoplasts; Fluo-4-AM, sperm)	115
*Dm*GLR3.4 (Dm_00004609-RA)	ND	Trap: trigger hair	ND	ND	130
*Dm*GLR3.6 (Dm_00002270-RA)	ND	Trap: trigger hair	ND	ND	61, 129, 130
*Os*GLR2.1 (LOC_Os09g25980) *Os*GLR3.2 (LOC_Os02g02540)	WT: K^+^ uptake in bacteria	All organs (plasma membrane)	Potentiation: Glu; Inhibitors: CNQX, DNQX (Ca^2+^ bioluminescence with aequorin, roots)	Ca2+; K^+^ (Indo-1-AM Ca^2+^ imaging, HEK cells)	112
*Os*GLR3.1 (LOC_Os04g49570)	KO: defective root development; inhibited elongation of the primary, adventitious, and lateral roots	ND	ND	ND	88, 112
*Os*GLR3.4 (LOC_Os07g33790)	KO: impaired root-to-shoot systemic wound signaling; diminished SWP amplitude and JA response; plant dwarfism; BR-regulated growth defects and reduced BR sensitivity	*Nicotiana benthamiana* leaves (plasma membrane)	Potentiation: Ala, Arg, Asn, Cys, Glu, Gly, Leu, Lys, Pro, L-Ser, Thr (Ca^2+^-VP, rice coleoptile epidermal cells)	Ca^2+^ (Ca^2+^-VP, rice coleoptile epidermal cells)	169
*Rs*GluR (AY328911)	OE (transgenic *Arabidopsis*): increased glutamate-induced [Ca^2+^]*_cyt_* elevations; morphological defects; stunted growth; local necrosis; enhanced resistance to *Botrytis cinerea*; upregulation of defense-related genes and amino acid metabolism-related genes	Hypocotyl (plasma membrane)	Potentiation: Glu (Fluo-4 AM staining, roots)	ND	71
*Sl*GLR3.5 (AB623205.1)	KO: impaired defense response gene expression; disrupted systemic electrical propagations	ND	ND	ND	156

Due to the variety of techniques used to characterize *At*GLR3.3 ligands, the following superscript annotations are used for clarity:

1 X-ray crystallography.

2 NES-YC3.6 reported Ca^2+^ imaging.

3 Microscale thermophoresis.

4 Membrane potential recordings.

5 Aequorin-based Ca^2+^ imaging.

6 Further, to previously demonstrate roles in stomata response to glutamate (174), *At*GLR3.5 has also been implicated in salicylic acid signaling and stomatal function (175).

Abbreviations: ABA, abscisic acid; ACC, 1-aminocyclopropane-1-carboxylic acid; Ala, alanine; D-AP5, D-2-amino-5-phosphonopentanoate; Asn, asparagine; Arg, arginine; *At*, *Arabidopsis thaliana*; BR, brassinosteroid; COS-7, CV-1 in Origin with SV40 genes; CNQX, 6-cyano-2,3-dihydroxy-7-nitroquinoxaline; cryo-EM, cryo-electron microscopy; Cys, cysteine; cyt, cytosolic; *Dm*, *Dionaea muscipula*; DNQX, 6,7-dinitroquinoxaline-2,3-dione; flg22, flagellin 22; Gln, glutamine; Gly, glycine; GSH, glutathione; GSSG, oxidized glutathione; HEK, human embryonic kidney; His, histidine; Iso, isoleucine; JA, jasmonic acid; KO, knockout; Leu, leucine; Lys, lysine; Met, methionine; ND, not determined; NES, nuclear export signal; OE, overexpression; *Os*, *Oryza sativa*; Phe, phenylalanine; *Pp*, *Physcomitrium patens*; Pto, *Pseudomonas syringae* pv. tomato DC3000; *Rs*, *Raphanus sativus*; Ser, serine; *Sl*, *Solanum lycopersicum*; SWP, slow wave potential; TEVC, two-electrode voltage clamp; Thr, threonine; Trp, tryptophan; Tyr, tyrosine; Val, valine; VP, vibrating probe; WT, wild-type; YC3.6, Yellow Cameleon 3.6.

## NEUROTRANSMISSION

Synapses impose barriers for neurotransmission, which are overcome by glutamate when it binds to specific iGluR subtypes to electrochemically connect neurons. The subtypes are defined by their agonist pharmacology and include α-amino-3-hydroxy-5-methyl-4-isoxazolepropionic acid receptors (AMPARs), *N*-methyl-d-aspartate receptors (NMDARs), and kainate receptors (KARs) (a fourth group, the δ-receptors, is not so well characterized). The release of glutamate into the synaptic cleft is initiated by a presynaptic depolarization activating voltage-gated Ca^2+^ channels (Ca_v_s), which evokes Ca^2+^-dependent exocytosis (90, 132). Glutamate binding to AMPARs and KARs with an EC_50_ as low as 1 μM initiates a rapid depolarization through a Na^+^ influx to propagate the electrical signal (147) (Figure 3a,b). While depolarized, a Ca^2+^ influx at the postsynaptic neuron occurs when glutamate plus glycine or d-serine binds to NMDARs (Figure 3a,b).

## DIFFERENT ENVIRONMENTS: DO PLANT GLUTAMATE RECEPTORS FUNCTION LIKE IONOTROPIC GLUTAMATE RECEPTORS?

Most functional hypotheses about plant GLRs took inspiration from their homology to mammalian iGluRs. The model of electrical and glutamatergic signaling exemplified by iGluRs during neurotransmission is complicated by various factors in plants and should be scrutinized for viable analogies of glutamate receptors or for where evolutionary divergence seems to predicate distinct GLR properties not yet described. First, the Na^+^ concentration in the apoplast is too low to generate a suitable chemical gradient for fast depolarization. Standing concentrations of ions are variant by cell type and species, but overall they are supported by a membrane transport system different from that of animals (5, 33, 34, 100). All other cells—except for animal cells—use H^+^-pumps to energize membranes by a pH gradient. In plants, this role is performed by P-type, auto-inhibited H^+^-ATPase (139). The H^+^ motive force contributes to a hyperpolarized resting membrane potential, allowing an anionic efflux, instead of the Na^+^ influx, to drive depolarizations. The plant action potential is thus orchestrated by H^+^, anions, Ca^2+^, and K^+^ (Figure 3c,d) (6, 26, 41, 52, 53, 57, 65, 77, 95, 117, 142, 148). Classes of plant anion channels [such as aluminum-activated malate transporters (ALMTs) and slow-type anion channel homologs (SLAHs)] have no mammalian homologs, and plants lack fast voltage-dependent Na^+^ or Ca^2+^ channels (54, 113). From a physiological and genetic standpoint, the evidence suggests that electric signaling in plants has taken a different path.

Secondly, glutamatergic signaling in plants lacks the specificity of that in neurons. Glutamate bolsters plant development through the regulation of hundreds of genes (17, 49, 69, 121, 154, 155). Despite incertitude and the emergence of recent conflicting data (17), glutamate and other amino acid concentrations in the apoplast seem to fall into the millimolar range (39, 59, 91), orders of magnitude above the measured EC_50_ of GLRs (74). And no data in plants support the clearance of extracellular glutamate that characterizes synapses and sustains their ligand-gated mode of action.

## SUMMARY POINTS

1. Plant glutamate receptors (GLRs) are pharmacologically and genetically associated with participation in electrical signaling and Ca^2+^ signaling (Figure 6).
2. GLR signaling participates in root morphogenesis and development; carbon and nitrogen metabolism; seed germination; reproduction; wound reactions; defense against herbivory; immunity; stomatal aperture regulation; light signal transduction and photosynthesis; amino acid responses; and hormonal signaling pathways associated with jasmonic acid, salicylic acid, and abscisic acid.
3. Roles in systemic wound- or herbivory-induced signaling, sexual reproduction (even encompassing the transition from the use of freely swimming sperm to pollen tubes), plant immunity, and root development have been documented in more than one plant species and appear to be conserved functions.
4. Overall patterns of gene expression are highly dynamic among all three clades of *Arabidopsis* and actively regulated by stressors, namely wounding.
5. General stress responses for plant immunity and wounding have been recognized to involve upregulation of *AtGLR*s from clade 2.
6. The atomic structure of *At*GLR3.4 resolved by cryogenic electron microscopy revealed a tetrameric ion channel adopting a nonswapped configuration among the individual subunits.
7. The binding of glutathione to the *At*GLR3.4 amino-terminal domain (ATD) presents a new mechanism for allosteric modulation of GLRs.
8. Structure determination of *At*GLR3.4 and isolated ligand-binding domains (LBDs) from *At*GLR3.2, *At*GLR3.3, and *At*GLR3.4 deposit the molecular framework for nondiscriminatory amino acid binding as well as ligand-gating properties. The ligand-binding pocket consists of a conserved sequence motif forming a ring and coordinates the ligand amino acid’s backbone atoms by the amino and carboxyl groups. With an amino acid bound to it, the LBD is observed to have a closed clamshell configuration, resembling conformational changes undergone in iGluR gating cycles.
9. Still awaiting consensus is an accurate calculation of free apoplastic amino acid concentrations—of both proteinogenic and nonproteinogenic varieties—and its consequences for ligand-gating and GLR-mediated signaling, as GLRs possess an apparent submicromolar binding affinity.

## Box

**Ionotropic receptors:** sometimes referred to as ligand-gated ion channels, the receptor is formed by a ligand-binding domain linked to an ion channel–forming transmembrane region

**Electrical signaling:** mechanism of cellular communication based on changes in the membrane potential caused by an ionic flux across the membrane

**Calcium signaling (Ca**^**2+**^ **signaling):** ubiquitous signaling form among eukaryotes, which uses changes in the cytosolic free concentration of calcium ions ([Ca^2+^]_*cyt*_) to perform secondary messenger functions that mediate intracellular processes—often a critical step in signal transduction

**Ion selectivity:** the preference of a channel to allow permeation of specific ionic species

**Gating:** the mechanism of opening and closing the ion channel pore; various stimuli, such as membrane voltage or ligand binding, commonly initiate it

**Depolarization:** a change in the membrane potential to more positive (or less negative) voltages

**Heterologous expression:** gene expression into an organism that does not naturally encode for the gene of interest; common systems include *Xenopus laevis* oocytes and mammalian HEK293T and COS-7 cells

**Intracellular membrane potential measurements:** electrophysiology technique used to directly monitor membrane potential changes through the impalement of cells with glass microelectrodes

**Membrane potential:** the difference in electrical voltage between the interior and the exterior of a biological cell

**Surface potential measurements:** extracellular, noninvasive method to measure the voltage potential variations emanating from the underlying cells and tissues from outside the leaf

**Electrical penetration graph (EPG):** a technique for taking electrical measurements using bio-electrodes inserted into the proboscis of a parasitic aphid, thus permitting access to the phloem sap

**EC**_**50**_: half of the maximal effective concentration of any ligand or elicitor

**Agonists:** molecules that serve as a ligand to activate a biological response, often initiating the opening of ion channels to pass a current

**Antagonists:** ligands that prevent the binding of another ligand to the same site; often have an inhibitory effect on channel current

**Protoplast:** intact nonanimal cell whose cell wall is removed enzymatically, displaying the exposed plasma membrane

**Holo:** the ligand-bound state of a ligand-gated channel

**Apo:** the ligand-free state of a ligand-gated channel
