# Supplementary material for: Merging Signaling with Structure: Functions and Mechanisms of Plant Glutamate Receptor Ion Channels
Source: Annu Rev Plant Biol. Author manuscript; Available in PMC 2024 Oct 15. (PMC11479355; doi:10.1146/annurev-arplant-070522-033255)
Supplement: Supplementary text [file NIHMS2025674-supplement-Supplementary_text.pdf]

Supplemental Material: Annu. Rev. Plant Biol. 2023. 74.
https://doi.org/10.1146/annurev-arplant-070522-033255
Merging Signaling with Structure: Functions and Mechanisms of Plant Glutamate Receptor Ion Channels
Simon, Navarro-Retamal, and Feijó

Downloaded from www.annualreviews.org. Guest (guest) IP: 129.2.89.114 On: Mon, 07 Oct 2024 14:58:42

Glutamate receptor sequences and selected accession numbers

```
>AvGluR1
MRIFRENISWLLLYVLHNLKSVMAIWPTPSNSSLQLLGFFPNAENTSQTKELSIHTQAMFKAAVLLSHQYNITIEGQ
YIQWQASFTDGNVMNTLGNTCRAISSSNIVGIIGPIFSREAHQIADFANKIGIPVVSSTATDSDLSNRDNYHAFYRT
VPSDSTIALALAKLFIRYNWTSCIIIYQSDVYGTGGTKVISETFLKYNIEVTDLIVFDMVMNSIRGNLRTYLTTSIS
RIVILWTDIVYISQILRYALDADILGPHFTWILTSSISLDSFDQIYHSKLIGILTIEPVTGTVVDAPINSTLLHAAY
QLWQQYEPESFPTSAKVNSYALFAFDATWTLIQSLQKFCSSLKDNSSSCSAYDGPLFCFDRHFIHSNLLFNIMNSLS
FLGVSGHVQFTMNVTDRVNGSYYYAQNIQYTSNHISFTPVLKYDSSDDWQTYSKTNVIIWPGNSLIPPIDRARLKGI
TLRIGVIESVPFTIVANVIDTSGRNTTKLTGYVLDLIEYLRDKMGFVADVQLAPPNTSYTGLVLAVANGDYDIAIGD
ITVTSARREIVAFSNSISDNSMRILMRKTPAIQVDLLSYLKPFSRNLWLLLLGATIFASIILCVIERPDNAALQNRS
IISSGAMILWFSFGTIVGYGADFHAQTAAGRLVSAGLYILSLVLVASYTANLASELTILKTKDLIDGMDDLKNGKIP
YNRIGIRIGTAGEDYYLREISGGSRNFYPLKSRQEMYDSLLAGIIDVSFMDIGTAEYVTNNIYCNLTLVGEDFDKST
FGIVTPKEWLYAKDLDVNILSLRETGILDNLKKKWFQTKACPQTSEISTALGLESLSGLFLTFGVICVLSIGLYAWN
KRNMIRKYVNILRHQKTISLRSETYMVDFTNKPHTSTTSSSTISQIIA


>CeGLR1
MFSSFSFLNMFGVLFTVFNLTVVQPYPSHIIIKSFGNNEEVSRVALKAMEYTSDHINSRDDVPFKLAFDH
RVVEEGAAVSWNMVNAVCDELKEGAMALLSSVDGKGREGIRGVSDALEMPLVSLTALSNDDHQQQQFGNL
FEVSVRPPISELLADFIVHKGWGEVLVLIDPVHASLHLPSLWRHLRTRTNTSVKASMFDLPADEKQFEAY
LMQFNMMRNNETNRILIDCASPKRLKKLLINIRSAQFNQANYHYVLANYDFLPYDQEMFQNGNINISGFN
IINKDGREYWSLKKHLKTSSSLGGGDDVSVEAAVGHDAMLVTWHGFAKCLQANDSLFHGTFRHRRFFNRG
FPGIYCDPLSDRSHPNRPFSSFEHGKTIGVAFRNMKIGHKEGTLTGNIEFDRFGNRKNFDVSIVDLVSNT
KATFNSKEVLAWRQGVGFFSNRTVAQHSRKSQNDHKDNQVIVLTNLVAPFVMIKRECLEMANLTECQGNN
KFEGFCIDLLKLLADKIEEFNYEIKLGTKAGSKQADGSWDGMIGELLSGRAHAVVASLTINQERERVVDF
SKPFMTTGISIMIKKPDKQEFSVFSFMQPLSTEIWMYIIFAYIGVSVVIFLVSRFSPYEWRVEETSRGGF
TISNDFSVYNCLWFTLAAFMQQGTDILPRSISGRIASSAWWFFTMIIVSSYTANLAAFLTLEKMQAPIES
VEDLAKQSKIKYGIQGGGSTASFFKYSSVQIYQRMWRYMESQVPPVFVASYAEGIERVRSHKGRYAFLLE
ATANEYENTRKPCDTMKVGANLNSIGYGIATPFGSDWKDHINLAILALQERGELKKLENKWWYDRGQCDA
GITVDGSSASLNLSKVAGIFYILMGGMVISMLAALGEFLYRSRIEARKSNSNSMVANFAKNLKSALSSQL
RLSVEGGAVAQPGSQSHNAIRRQQVAAFLPANEKEAFNNVDRPANTLYNTAV

>CeGLR2
MNKNLLVFGFLIFVKIGETSKKFPLRAFVASTDIDNDTAHAIIEMLRLAEMTFNALSDVDFDVLLGTRDLPPMEMAT
MMWNLNRIICDEMKLGYMLMLAGTNFKNYGIYEDIANHMKMPLIDWEPSKSENIGKTTENNPMIFSVAPSAEQLLID
YIQYKGWRDVVYIHDGKNADRTLRTMFSYLHEKSPKYQLFVDNYVAPSDEEMFKEFLNEFHRRISTQHTLKSNDSSE
EIDEPIPVNVIVDLEGSYRTRAFLRALEESVLVKKEYHYVFSNFDVDETDLSGFHFSLINITIFRIFDKNNKKFLKT
RAEFHDVYRGGFSNTDSIPTAAAFAHDAILVAGKALQIAMNEHGKGIFDKSFVRHQLFNRGRKGLYCRPHEDQTESR
QFETFEHGKKIAEAIKKVVLTDKDGTLTGRIQFDKVTGKRTNFSAEIVEIKPGVNSLNSIWERFQWAEGEGFLLGGE
RYVQEKKKDSSQTRKGILPSKPWQLRFNVVTVLVKPFVMLKRRNPGEPELKGNDRFEGYCIDLLNLLAKNITGFEYD
VFISDGNKYGSRQADGSWDGMIGYLLNETADVAVAPLTITQERERAVDFSKPFMTTGISIMIKKPEKQEFNIFSFME
PLGMTIWIFTLSSYFGVSLTIFLVSWFSPYEKRIEFKRGEFTVTNEFTLYNSLWFTLAAFMQQGTDILPRAVSGRIA
SSCWWFFTLIIVSSYTANLAAFLTLERMTPPIESVEDLANQNKILYGVNEGGSTAAFFEDSIVPLYKKMWNFMVSTT
QKQIELEKQSITNSTSNRIFVSSYADGIEKVRTSKGKYAFLLEETTNNYESGRRPCDTMKVGQNLNTLGYGIATKIG
NPLRVSLNLAILYLSEKGELKKLENKWWYDRGQCDTGTSDGGTSSSLNLSKVAGIFYILLAGMVLSMCTALVEFLFR
KNKENREKERNRMRSSRPLKPGILASCERAKQKQLQNRRTKSEEVSTPRSTLF

>CeGLR3
MFWIAKTLIAFLILLKTDCYKIAIPANLIDEVNPVLEFVDFRVQVIPYETKPLWRIKQESFKIVGISIENGLFSVCN
CLILGASAIILPEQYDGHLAAAAIVQSIADNTNVPCVSLHLSPPTRPSTHLHPHLNAKSLAVAAFIKREKWKDVVVV
FEEPDELLEITDMITAGHFDPDSFSSQLVRLKHGDDYGNELKHIKNKLDRYRIVINIPLQKALHFLEQAANMSMCGV
LYHYVVMDMDLVTVDIDSIRGIEDCNITSFGVHDVNSEYIEDIRQEIVHKSSIRLPKKGVPYTTSIWIDTLRLLIRS
MKSIQIWDEPRCGSSWKSGSDIKKRFFENPLAGISGDLHWAPSGERSNYTLHVYRRTLSFQKFAEWSSRTRRIASSE
AVVIANSSEKLTLEGKHLKISVYLEAPFVMITSNGSYEGYCIDLLHKIANILKFTYTIQKVRDNAYGSKESNGKWSG
MVGELQRGDADLAVASLTISYGRSEVIDFTVPYMHLGISILFKKPRIRDSDWFKFMDPLSTQVWIMTFASYFVVSVA
IWIIAKISPYEQFERDEDNGQYKPVDNQFSLRNSFWFTVCSLMQQGSELCPRAASTRLLTGIWWFFALILISSYTAN
```

LAAVLTTRRMETPIENADDLAAQTKIKYGTLGRGSTMSFFNESKIETYERMWQLMSSSPGLFVQSSKEGIARVKSSD
YAYLMESSMLEYAVERDCELMQIGGLIDQKGYGIGLPKGSPYRELISTAILRLQEKTELTELKEKWWKDKSVVCEQP
KRKDQDDGESIGGIFIILVVGLVLTAVLVIFELITTRKPSPAQSQVIRHVNVIPSFKLGFFRWNVN

>CeGLR4
MQEKCRLVRGFSILLVIFYIANSAADDRFHIGTIANRGSFAYEHLRYAIDRWNTEHGAHTQIKFSIVSPIRYDNNYE
ERMCEIMQQGIVAVVLSNEESEQDSQLIKSMCHYFNIPCLSLQSTSLRDSVSDFVTLLGPSRGAGARATSEFLDSMR
WTGFLLAYQHGSDLEDLSPLMQYKQIVDTGGRRIHIKIRRLPNNTDDYEPFLKYVKTRLKQTNIIIHSNNITVLYNL
LQQARGLNMAEPPFSYVFTNTDLSLLEDFLNNMYGASFHCNITGLQLVKNDPMMKTQLALTSEAVYVVGMSIYRMRE
LGHAPRQSSIMCDSHDIWSDGRIMNEGIRKLKLRNQLTGDVQFKSNGERDDIMYHGVGRINSQFVKLGNWSEKRGWN
FDSRYANRWEFDIDPDSEDLEGLHLRVVVYLEEPFVIKTGENQYEGFCIDLLNEMTQVLKFNYTIIEVQDGTYGIED
ESGRWNGIIGALQRHEADLSLSAVTITYSRAEVVDFTLPFMHLGISILLARTSEETDKGSLWTFLEPLSLTVWISLL
ISYCIVSYSMHILAKFSPYEWYNLERIDERDFENIKNQKNQFTVLNSFWFTMGSLMQQGSDVIPRAAATRLIAVVWW
MFTQIIISSYTAQLAAFLTVERMSTPIESTQDLANQQKIRYGVLKSGSTMDFFRESKIPMYERMWSVMESSSPGVFV
NSSREGIARVKSGGYAYMMESSMLEYYLERDCELQSIGGLLDSKGYGIALPKGSPLRDILSRTVLQLQERTILEALK
NKWWRDRREGPSCGPPPSEKATNSKPQNIFGIFYVLLTGLIVAFLLACGEYCIESRHEAFRLKLTIVGKFMDWYRGN
SNDSKQRSKLRNMQLDTAGQEVSISTRHDPPTPPQPALTSRPDPTRLSISSPAPTICVVSCDDGTPISRDRPPSISR
AGLRRLSRMLPAATHEQLSRFRQGSMS

>CeGLR5
MQFNNNYIFSIFTILHVFSTSCIAYKIGAIFREREDAHIEAAVRYSVEWLSQRGIYGDIDLVIEYIDVLDHYDAIKK
ACKMLEKDHIVALLGGSHAALNAQLERITDDLDIPFLTAIDDLRTDMGKSKIDFWPRPQLFEAVVDMFTHWRWNRIV
LVYEGDERIRRLEQLLESEEYASIRFYLIKVHDGDYMKAARQVKELEECRLLHKKDCSEFSRLLVDMNPEHTYTFLL
ASLQMGLIELKHWFLLTNMELSTMDMELFRYNHARFISPYPVDSTFLTENRDAFNFSHFKEHITEKWAMKTDNSRNL
KMMEAVFTFDAVYAFANVFNELSSHMQMNDVPQTLCRKSSRNSRKYQHGRSLIDNIVHNDLHGLSGDLRRLNGHPLK
SNFSMRIQLLGYSGRLDDIGFWEPATNVHVNMSGDSKAQLQRNVQVSDELKPHFRVTTIMERPYVMLKKNHYELDAN
SKFEGFCIDLLAELSKDLGFTYTIHAVKDGKYGNDKYGNGSWDGMMGEILRGEAEMAVAPLTVNYRRSEAVDFTKPF
LSLGISILYKVPDDQQPDLFSFLNPLSWQIWTAIATSIITVTLGMYFVANVTPYEWNLNFSCCTAHEPHPAAAFATN
QEAPIVMSNNYSFWNTVWYVLSTMLKGGCDFGPRAVSTRLLGGTWWVFYLVIISAYTANLAAVLTVSRPYIPIKNLD
DLANQTTISYGTIRGGSTMQFFQESRIAAHVKMWQYMKDKDVFVTSNGKGVERALSMNYAYLMESTSLEYETQQNCN
LTQIGGVLGSKGYGIALAKKSEWTDRISRQILLYAKRGIIEMKKTKWWRSKGAACASTASAVKHDRFALSMYNVAGL
FITLGVGIVLAAIVVIFELIHRCHHIAKKEGKPFLEELLYELRFALNMNSLSDHRSRQKMSTCNSNGNGKPNHLNEN
GHLKKTTI

>CeGLR6
MLNIFRFLLLSFLGTGVSSIRIGLITSHHLEHSVRTAVNIIVPRINEQIVKTRGACFITHTSFIALKNSHEDLNTKI
CDLANSEVSVIIGVVDRESGSIIEEQCAELNILFIHYYWTPGYQKKQQTSLNLYPSMEYSQLMERLINLWRWDNFVY
IYSNYDAPKQLIETLSRLEKSPALLRAHTLDDSMMATALALRDTCDRNRCWPKKNRVLIELSPNETLTFFDASLKLG
MISVHNWFLITALDDLNDHLSQYTHNGMRVSLLTVSKEKWNQNDLAIKLPDMYQEYLAKIPSNPRTPFKDFAFIFDS
ILLACQAGDRRIISCGDNEAVKKFKLPIVKPFKGLTGKISFNGTSDRSDSELHIWEMGITGAGLHTGIWKSTWEGSK
ELTMLAKNIPGTHEHYQASVRESRTLKVTSIHEKPYVIEKIMPDGRIKHEGFCVDLLDKLAEMLHFNYTLKIVKDNK
YGERKNGTDEWDGMIGEILRGDADMAVAPITVTATRLEVIDFTDPFLQLGISMLMRQPNPKSSSSLTRFLWPLSASV
WTFSAIATVITALLVTVAAVLSPKESTAEFKIQNSVWYLVCILLRAGSGYNCQAGATRLISAVWWSFTLVLIAQYTA
NFAALLTVDRKSMPFNSFEELGNQTEYNFGSILGGSTMQFFKYSRIETFRRLWERMQSAEPSAFVGTNHEGVNRVLN
EKYVFLMESATLDYQVTQNCNLTRVGNVVLGSNGYSIALPKGSKWREKLTRQILDLNEKGIILMLKNNWWKKSQQEC
QSSEPEDLQTALGAENVYGLFLLLALGSGIGVLCAVLEHTHFIFFEKNKRNGQTPKLQQMIETIHAEIRNSPKF

>CeGLR7
MFFVDKYRVDVISLERYDFNDSSDINSLCDQMVNVSFVISVCRSNDSISLVAELARISQVPTIQVDLNYWSLPLNFN
GTSVPTISSPSYTTMVLSTLVFQNVITDIFADLNITPNSTVLYDNMYPPDFTAWRDAFSVLPGIRFVPMETTVLRMR
AQITQLRMASINSLILVAKTENVERFTLEAADFIEQRVFNTYVLTKDITAFKCDSCESAFMFWLRPFPVGKLMEIRD
LDDYLFRNEIGLELDYSINGWDSLNVAFYMNVMGYAFETIRTMNATFDVLPTFTCQTGPSEPNITTTVRDIILNDPA
HEYGNYTEAGLNMFFQDVEVRIYKIDRDRDHEDALFNKEVGVWSPISKLSVMYGTLQVDVRNLNIFRVVTLIQPPFV
QRTGDPDTPYEGYCIDLINMIQVEVNFTYTIYEVEDGSFGTMDDNGNWNGLIGALVSGSADIALAPLSVMAERENDV
DFTVPYYDLVGTTILMKKADVEYSLFKFMKVLEWPVWLCIVAAYLFTSILLWIFDRFSPYSFTNNKERYQNDIEKRQ
FSLKECLWFCMTSLTPQGGGEAPKNISGRLVAATWWLFGFIIIASYTANLAAFLTVSRLEQPISSLDDLAKQYKIEY
APIKGSASETYFRRMAEIEETFYNMWKEMSLNESMSPRDRAKLAVWDYPVSDKFTNMWRYMQESKLPVNMDTAVDRV

Downloaded from www.annualreviews.org. Guest (guest) IP: 129.2.89.114 On: Mon, 07 Oct 2024 14:58:42

LNSVDGFAFIGDATEIKYAALTNCNLQQVGTEFSRKPYAIAVQSGHILKDKISSAILMLLNERRLETLKEKWWTDNP
NKVSCPDSSDESDGISIQNIGGVFIVILAGIALSIVTLAFEYYYYKRKARKAAEKEQEELEMKQSQQVVPNGFHTNG
VKQDAPKLTNGTKTNGNTFRRTKRSNSVSAYENTAFQY

>CeGLR8
MKGRTLKMVVPAIEPPYVNYVNFSDAAVTDKGYGPGVVMEILKEIGKRLNLTYEILPALGSTWGEYLNGSWTGAFGQ
LVRGEVDLLAGGAIMEYDRSVIADLTYPFQFEPTGIMIRSPEKYEDDTLLIVTEPFSWEVWVITAAVILISGVIFLV
MTNIIRKVYEEMTVTPFESIWVFFSIFVQQGLPEQPRSWSCRVLVALWWLASITLSATFTGSLVALFAVDKTNVPFQ
NIDQLVRLVKQGKFEIVMDENSFTRTEMIARSKLPVYRDLWHEMIVNHKVKYVNGIARGVAFVRANPGYALLGPMAT
LNFYAYSDCKVILFNDGILPVYLSIPLVKNSIYSPYFSTKIREMVERGFTQKWIADYRSYVAMQKINECNSTTIGPK
SYLDLKRAQGAFWVFLGGAGLGLALFVGEFIFKFFREKMTKHTKTTTVPPEVATVSANVAENSTSLDIPDEVLPSAP
LPSEDGNWSLRRRNILNLSLDLSNIAN

>CeNMR-1
MFRISVIFIWIFLQLGYTIDYKVSVLIVSEPNQETFKELKVSVTAAFVEVFGSTSYHLGNDTISAAFVDA
KSGDNRLELTQDIVCSQMLNHSLASVIFSPLLTSSSRFIDLVTSSAYTLSFYKLPVVGVMVRDAEFSKKN
IYPTFVRPTAPLSDEAFVFLHMLLSLKYRQVVVLSVKRDINADQFVEEFEKRRVEFKIIVQRYIEVELNE
NLNDTLAESFEEVTSNIIVLFAKKDDAVRIFANAGDLTGKGKVWIVSESAGEAHNVPNGSLGCRLGQTAF
SVLRDSFSILKSAMETIFRESKIDIFPPVECDRDSVDAEWNSLQAPALLNEICGTSTSRVHFNDKCERIG
VEYDIINFHMERKQVGNMVGDILRLDEDSIEWAGGTKPLEISLPKHLRVVTVADPPFVYTTPIGSPSQCA
ELGNTVVEWSIFDKIVVSGPWYSCPLTLENSTEYFCCAGLAIDLLSNLSLPEANNSIDTSFTFSLHLNES
YGVVQASETTGITISGVIGELDGDTADMAIGGITINPERERIVDFTEPWLYHGIRILEKNIPRDSPMQSF
LQPLQSSLWTALFISVILVGLAIYCLDFKSPFERFYQADKEMEQDLKKEFELWIGKDADENVNFGEAMWF
VWGVLLNSGVSEKTPRSCSARVLGIVWCGFCMIMVASYTANLAAFLVLDQPEKGLTGVTDPRLRNPSANF
SFGTVLNSNVYQYFKRHVELSSMFRKMEPHNVRRASEAVHSLLNGSLDAFIWDSTRLEFEAARHCELRTR
GSLFGRSAYGIGLQKNSPWTPHITSAILRMSESGVMEKLDQKWIDRGGPNCVVEAHKSPARLGLVNMKDI
FILVSSGVALGIFLSFVEVSYGRRLADKGRRRRIVTRYFQKWHDLTLGKKRRPYRLKYNLDRMIVRRGFS
GLERCSFQELRERRQIRGLPTSKVDPYCFWPDLDFKDKPLVLFCSRCRNIVESDVHRETGLFAFLSCFLF
AILFLWPCSPLPCFLSSFSDFVHICPLCSHIMGRFRRARSTRFYV

>CeNMR-2
MKHRIWFILLLLATFFEKNQSIDLGGSRKVQKPKEDPKREDVNIAVVYQHYAGRSKSYDKAFKEVIRKIN
DATAVSSLRRLATRYNFQAVDCILPTGTFFVKEVLDCLCNNVTSNNVALIIFVTASETYDSTTAAEQYFL
TAASYTGIPIIAWNADNAGFTFENDLGPYRIIQMAPPIEHQARAMLALLRRYNWPKFGVVTSEMAGNDRF
VTAIREELELFSNKSTKFEMIHFSHMNTKNDTDVDLKLADVKKNQAKIILLYANAAQAGTIFSHAEKMDM
IGEKYLWIGTQSVKGTQTTVKAPAQAGMLCVNFHTVSNAMFAPRDDILPLIIQLAPKLFGAALLQLRPNE
MFSLKSNVSCKSEDGDPYWDNGKYIYEHMKAAFVKGNPFHVDDGHDSFFYTFEKTGRLRNSILQISNLRT
NSKGEKTWEKVGIFTNNELKMADVQWPGEKANPPQGAADKFHVKVVTLHEPPFIAVSDVDPDTQKCPGNQ
GSICDWGDVEYTDGAGVKKNRTLLKCCSGYCVDLLNKLANDIGFTYTLYKVRDEKWGLKTENGWNGLIAD
LMHNKADMCVTSLKLNSERARDIDFSLPFLDTGISIIVKIRSGVLSPTAFLEPFEYSTWVIILFVCIHVA
AISIFLFEWVSPYSFNMQKYPPPEHKFSLFRSYWLVWATLFSASVSTDVPKSTVSRLMALVWAAFGLTFL
AVYTANLAAFMITRVQYYDLSGIHDPMLNFPHDQKPPFRFGTVDGGNTHETMKRNWHKMHEYVKHNKYFR
MNISAGIEAVKNEELDAFIYDAVVLDYWAGKDANCALMTVGKWASMTGYGIGFPKNSPHTSLVNHYMLQY
QQKGDLERLQNFWLTGACTPDSHSQTQSAPLGIENFLSAFVLLAGGIIVSVIVLGFEHIYCMHLRKPLQK
IDPNGWCGIISMAMGKSLTFTEAVDRVQEWRSRTQSLASTNSPQLKRRRSANLRPTEEPFNQDLNPPRRS
PRFLQVETNL

>GluR0
MLILRRLILVVSLSFATIAIDITTRPGLEPVQGQGVETVDSQTLKVGVVGNPPFVFYGEGKNAAFTGISLDVWRAVA
ESQKWNSEYVRQNSISAGITAVAEGELDILIGPISVTPERAAIEGITFTQPYFSSGIGLLIPGKPVSLWERFSPFFG
IAALSSAGVLTLLLFLVGNLIWLAEHRKNPEQFSPHYPEGVQNGMWFALVTLTTVGYGDRSPRTKLGQLVAGVWMLV
ALLSFSSITAGLASAFSTALSEASATPLFRSVGDLKNKEVAVVRDTTAVDWANFYQADVRETNNLTAAITLLQKKQV
EAVMFDRPALIYYTRQNPNLNLEVTEIRVSLEPYGFVLKENSPLQKTINVEMLNLLYSRVIAEFTERWLGPGIEENQ
DLLPQNIGESPS

Downloaded from www.annualreviews.org. Guest (guest) IP: 129.2.89.114 On: Mon, 07 Oct 2024 14:58:42

>GluD1
MEALTLWLLPWICQCVSVRADSIIHIGAIFEENAAKDDRVFQLAVSDLSLNDDILQSEKITYSIKVIEANNPFQAVQ
EACDLMTQGILALVTSTGCASANALQSLTDAMHIPHLFVQRNPGGSPRTACHLNPSPDGEAYTLASRPPVRLNDVML
RLVTELRWQKFVMFYDSEYDIRGLQSFLDQASRLGLDVSLQKVDKNISHVFTSLFTTMKTEELNRYRDTLRRAILLL
SPQGAHSFINEAVETNLASKDSHWVFVNEEISDPEILDLVHSALGRMTVVRQIFPSAKDNQKCTRNNHRISSLLCDP
QEGYLQMLQISNLYLYDSVLMLANAFHRKLEDRKWHSMASLNCIRKSTKPWNGGRSMLDTIKKGHITGLTGVMEFRE
DSSNPYVQFEILGTTYSETFGKDMRKLATWDSEKGLNGSLQERPMGSRLQGLTLKVVTVLEEPFVMVAENILGQPKR
YKGFSIDVLDALAKALGFKYEIYQAPDGRYGHQLHNTSWNGMIGELISKRADLAISAITITPERESVVDFSKRYMDY
SVGILIKKPEEKISIFSLFAPFDFAVWACIAAAIPVVGVLIFVLNRIQAVRAQSAAQPRPSASATLHSAIWIVYGAF
VQQGGESSVNSMAMRIVMGSWWLFTLIVCSSYTANLAAFLTVSRMDNPIRTFQDLSKQVEMSYGTVRDSAVYEYFRA
KGTNPLEQDSTFAELWRTISKNGGADNCVSSPSEGIRKAKKGNYAFLWDVAVVEYAALTDDDCSVTVIGNSISSKGY
GIALQHGSPYRDLFSQRILELQDTGDLDVLKQKWWPHMGRCDLTSHASAQADGKSLKLHSFAGVFCILAIGLLLACL
VAALELWWNSNRCHQETPKEDKEVNLEQVHRRMNSLMDEDIAHKQISPASIELSALEMGGLAPTQTLEPTREYQNTQ
LSVSTFLPEQSSHGTSRTLSSGPSSNLPLPLSSSATMPSMQCKHRSPNGGLFRQSPVKTPIPMSFQPVPGGVLPEAL
DTSHGTSI

>GluD2
MEVFPFLLVLSVWWSRTWDSANADSIIHIGAIFDESAKKDDEVFRTAVGDLNQNEEILQT
EKITFSVTFVDGNNPFQAVQEACELMNQGILALVSSIGCTSAGSLQSLADAMHIPHLFIQ
RSTAGTPRSGCGLTRSNRNDDYTLSVRPPVYLHDVILRVVTEYAWQKFIIFYDSEYDIRG
IQEFLDKVSQQGMDVALQKVENNINKMITTLFDTMRIEELNRYRDTLRRAILVMNPATAK
SFITEVVETNLVAFDCHWIIINEEINDVDVQELVRRSIGRLTIIRQTFPVPQNISQRCFR
GNHRISSTLCDPKDPFAQNMEISNLYIYDTVLLLANAFHKKLEDRKWHSMASLSCIRKNS
KPWQGGRSMLETIKKGGVSGLTGELEFGENGGNPNVHFEILGTNYGEELGRGVRKLGCWN
PVTGLNGSLTDKKLENNMRGVVLRVVTVLEEPFVMVSENVLGKPKKYQGFSIDVLDALSN
YLGFNYEIYVAPDHKYGSPQEDGTWNGLVGELVFKRADIGISALTITPDRENVVDFTTRY
MDYSVGVLLRRAEKTVDMFACLAPFDLSLWACIAGTVLLVGLLVYLLNWLNPPRLQMGSM
TSTTLYNSMWFVYGSFVQQGGEVPYTTLATRMMMGAWWLFALIVISSYTANLAAFLTITR
IESSIQSLQDLSKQTEIPYGTVLDSAVYEHVRMKGLNPFERDSMYSQMWRMINRSNGSEN
NVLESQAGIQKVKYGNYAFVWDAAVLEYVAINDPDCSFYTIGNTVADRGYGIALQHGSPY
RDVFSQRILELQQNGDMDILKHKWWPKNGQCDLYSSVDTKQKGGALDIKSFAGVFCILAA
GIVLSCFIAMLETWWNKRKGSRVPSKEDDKEIDLEHLHRRVNSLCTDDDSPHKQFSTSSI
DLTPLDIDTLPTRQALEQISDFRNTHITTTTFIPEQIQTLSRTLSAKAASGFTFGNVPEH
RTGPFRHRAPNGGFFRSPIKTMSSIPYQPTPTLGLNLGNDPDRGTSI

>GluA1
MPYIFAFFCTGFLGAVVGANFPNNIQIGGLFPNQQSQEHAAFRFALSQLTEPPKLLPQIDIVNISDSFEM
TYRFCSQFSKGVYAIFGFYERRTVNMLTSFCGALHVCFITPSFPVDTSNQFVLQLRPELQEALISIIDHY
KWQTFVYIYDADRGLSVLQRVLDTAAEKNWQVTAVNILTTTEEGYRMLFQDLEKKKERLVVVDCESERLN
AILGQIVKLEKNGIGYHYILANLGFMDIDLNKFKESGANVTGFQLVNYTDTIPARIMQQWRTSDSRDHTR
VDWKRPKYTSALTYDGVKVMAEAFQSLRRQRIDISRRGNAGDCLANPAVPWGQGIDIQRALQQVRFEGLT
GNVQFNEKGRRTNYTLHVIEMKHDGIRKIGYWNEDDKFVPAATDAQAGGDNSSVQNRTYIVTTILEDPYV
MLKKNANQFEGNDRYEGYCVELAAEIAKHVGYSYRLEIVSDGKYGARDPDTKAWNGMVGELVYGRADVAV
APLTITLVREEVIDFSKPFMSLGISIMIKKPQKSKPGVFSFLDPLAYEIWMCIVFAYIGVSVVLFLVSRF
SPYEWHSEEFEEGRDQTTSDQSNEFGIFNSLWFSLGAFMQQGCDISPRSLSGRIVGGVWWFFTLIIISSY
TANLAAFLTVERMVSPIESAEDLAKQTEIAYGTLEAGSTKEFFRRSKIAVFEKMWTYMKSAEPSVFVRTT
EEGMIRVRKSKGKYAYLLESTMNEYIEQRKPCDTMKVGGNLDSKGYGIATPKGSALRNPVNLAVLKLNEQ
GLLDKLKNKWWYDKGECGSGGGDSKDKTSALSLSNVAGVFYILIGGLGLAMLVALIEFCYKSRSESKRMK
GFCLIPQQSINEAIRTSTLPRNSGAGASGGGGSGENGRVVSQDFPKSMQSIPCMSHSSGMPLGATGL

>GluA2
MQKIMHISVLLSPVLWGLIFGVSSNSIQIGGLFPRGADQEYSAFRVGMVQFSTSEFRLTPHIDNLEVANSFAVTNAF
CSQFSRGVYAIFGFYDKKSVNTITSFCGTLHVSFITPSFPTDGTHPFVIQMRPDLKGALLSLIEYYQWDKFAYLYDS
DRGLSTLQAVLDSAAEKKWQVTAINVGNINNDKKDETYRSLFQDLELKKERRVILDCERDKVNDIVDQVITIGKHVK
GYHYIIANLGFTDGDLLKIQFGGANVSGFQIVDYDDSLVSKFIERWSTLEEKEYPGAHTATIKYTSALTYDAVQVMT
EAFRNLRKQRIEISRRGNAGDCLANPAVPWGQGVEIERALKQVQVEGLSGNIKFDQNGKRINYTINIMELKTNGPRK

Downloaded from www.annualreviews.org. Guest (guest) IP: 129.2.89.114 On: Mon, 07 Oct 2024 14:58:42

IGYWSEVDKMVVTLTELPSGNDTSGLENKTVVVTTILESPYVMMKKNHEMLEGNERYEGYCVDLAAEIAKHCGFKYK
LTIVGDGKYGARDADTKIWNGMVGELVYGKADIAIAPLTITLVREEVIDFSKPFMSLGISIMIKKPQKSKPGVFSFL
DPLAYEIWMCIVFAYIGVSVVLFLVSRFSPYEWHTEEFEDGRETQSSESTNEFGIFNSLWFSLGAFMQQGCDISPRS
LSGRIVGGVWWFFTLIIISSYTANLAAFLTVERMVSPIESAEDLSKQTEIAYGTLDSGSTKEFFRRSKIAVFDKMWT
YMRSAEPSVFVRTTAEGVARVRKSKGKYAYLLESTMNEYIEQRKPCDTMKVGGNLDSKGYGIATPKGSSLGNAVNLA
VLKLNEQGLLDKLKNKWWYDKGECGSGGGDSKEKTSALSLSNVAGVFYILVGGLGLAMLVALIEFCYKSRAEAKRMK
VAKNPQNINPSSSQNSQNFATYKEGYNVYGIESVKI

>GluA3 MGQSVLRAVFFLVLGLLGHSHGGFPNTISIGGLFMRNTVQEHSAFRFAVQLYNTNQNTTEKPFHLNYHVD
HLDSSNSFSVTNAFCSQFSRGVYAIFGFYDQMSMNTLTSFCGALHTSFVTPSFPTDADVQFVIQMRPALK
GAILSLLSYYKWEKFVYLYDTERGFSVLQAIMEAAVQNNWQVTARSVGNIKDVQEFRRIIEEMDRRQEKR
YLIDCEVERINTILEQVVILGKHSRGYHYMLANLGFTDILLERVMHGGANITGFQIVNNENPMVQQFIQR
WVRLDEREFPEAKNAPLKYTSALTHDAILVIAEAFRYLRRQRVDVSRRGSAGDCLANPAVPWSQGIDIER
ALKMVQVQGMTGNIQFDTYGRRTNYTIDVYEMKVSGSRKAGYWNEYERFVPFSDQQISNDSSSSENRTIV
VTTILESPYVMYKKNHEQLEGNERYEGYCVDLAYEIAKHVRIKYKLSIVGDGKYGARDPETKIWNGMVGE
LVYGRADIAVAPLTITLVREEVIDFSKPFMSLGISIMIKKPQKSKPGVFSFLDPLAYEIWMCIVFAYIGV
SVVLFLVSRFSPYEWHLEDNNEEPRDPQSPPDPPNEFGIFNSLWFSLGAFMQQGCDISPRSLSGRIVGGV
WWFFTLIIISSYTANLAAFLTVERMVSPIESAEDLAKQTEIAYGTLDSGSTKEFFRRSKIAVYEKMWSYM
KSAEPSVFTKTTADGVARVRKSKGKFAFLLESTMNEYIEQRKPCDTMKVGGNLDSKGYGVATPKGSALGN
AVNLAVLKLNEQGLLDKLKNKWWYDKGECGSGGGDSKDKTSALSLSNVAGVFYILVGGLGLAMMVALIEF
CYKSRAESKRMKLTKNTQNFKPAPATNTQNYATYREGYNVYGTESVKI

>GluA4
MRIICRQIVLLFSGFWGLAMGAFPSSVQIGGLFIRNTDQEYTAFRLAIFLHNTSPNASEAPFNLVPHVDN
IETANSFAVTNAFCSQYSRGVFAIFGLYDKRSVHTLTSFCSALHISLITPSFPTEGESQFVLQLRPSLRG
ALLSLLDHYEWNCFVFLYDTDRGYSILQAIMEKAGQNGWHVSAICVENFNDVSYRQLLEELDRRQEKKFV
IDCEIERLQNILEQIVSVGKHVKGYHYIIANLGFKDISLERFIHGGANVTGFQLVDFNTPMVTKLMDRWK
KLDQREYPGSETPPKYTSALTYDGVLVMAETFRSLRRQKIDISRRGNAGDCLANPAAPWGQGIDMERTLK
QVRIQGLTGNVQFDHYGRRVNYTMDVFELKSTGPRKVGYWNDMDKLVLIQDMPTLGNDTAAIENRTVVVT
TIMESPYVMYKKNHEMFEGNDKYEGYCVDLASEIAKHIGIKYKIAIVPDGKYGARDADTKIWNGMVGELV
YGKAEIAIAPLTITLVREEVIDFSKPFMSLGISIMIKKPQKSKPGVFSFLDPLAYEIWMCIVFAYIGVSV
VLFLVSRFSPYEWHTEEPEDGKEGPSDQPPNEFGIFNSLWFSLGAFMQQGCDISPRSLSGRIVGGVWWFF
TLIIISSYTANLAAFLTVERMVSPIESAEDLAKQTEIAYGTLDSGSTKEFFRRSKIAVYEKMWTYMRSAE
PSVFTRTTAEGVARVRKSKGKFAFLLESTMNEYTEQRKPCDTMKVGGNLDSKGYGVATPKGSSLGNAVNL
AVLKLNEQGLLDKLKNKWWYDKGECGSGGGDSKDKTSALSLSNVAGVFYILVGGLGLAMLVALIEFCYKS
RAEAKRMKLTFSEATRNKARLSITGSVGENGRVLTPDCPKAVHTGTAIRQSSGLAVIASDLP

>GluK1
MEHGTLLAQPGLWTRDTSWALLYFLCYILPQTAPQVLRIGGIFETVENEPVNVEELAFKF
AVTSINRNRTLMPNTTLTYDIQRINLFDSFEASRRACDQLALGVAALFGPSHSSSVSAVQ
SICNALEVPHIQTRWKHPSVDNKDLFYINLYPDYAAISRAILDLVLYYNWKTVTVVYEDS
TGLIRLQELIKAPSRYNIKIKIRQLPSGNKDAKPLLKEMKKGKEFYVIFDCSHETAAEIL
KQILFMGMMTEYYHYFFTTLDLFALDLELYRYSGVNMTGFRLLNIDNPHVSSIIEKWSME
RLQAPPRPETGLLDGMMTTEAALMYDAVYMVAIASHRASQLTVSSLQCHRHKPWRLGPRF
MNLIKEARWDGLTGHITFNKTNGLRKDFDLDIISLKEEGTEKAAGEVSKHLYKVWKKIGI
WNSNSGLNMTDSNKDKSSNITDSLANRTLIVTTILEEPYVMYRKSDKPLYGNDRFEGYCL
DLLKELSNILGFIYDVKLVPDGKYGAQNDKGEWNGMVKELIDHRADLAVAPLTITYVREK
VIDFSKPFMTLGISILYRKPNGTNPGVFSFLNPLSPDIWMYVLLACLGVSCVLFVIARFT
PYEWYNPHPCNPDSDVVENNFTLLNSFWFGVGALMQQGSELMPKALSTRIVGGIWWFFTL
IIISSYTANLAAFLTVERMESPIDSADDLAKQTKIEYGAVRDGSTMTFFKKSKISTYEKM
WAFMSSRQQTALVRNSDEGIQRVLTTDYALLMESTSIEYVTQRNCNLTQIGGLIDSKGYG
VGTPIGSPYRDKITIAILQLQEEGKLHMMKEKWWRGNGCPEEDNKEASALGVENIGGIFI
VLAAGLVLSVFVAIGEFIYKSRKNNDIEQAFCFFYGLQCKQTHPTNSTSGTTLSTDLECG
KLIREERGIRKQSSVHTV

Downloaded from www.annualreviews.org. Guest (guest) IP: 129.2.89.114 On: Mon, 07 Oct 2024 14:58:42

>GluK2
MKIIFPILSNPVFRRTVKLLLCLLWIGYSQGTTHVLRFGGIFEYVESGPMGAEELAFRFAVNTINRNRTLLPNTTLT
YDTQKINLYDSFEASKKACDQLSLGVAAIFGPSHSSSANAVQSICNALGVPHIQTRWKHQVSDNKDSFYVSLYPDFS
SLSRAILDLVQFFKWKTVTVVYDDSTGLIRLQELIKAPSRYNLRLKIRQLPADTKDAKPLLKEMKRGKEFHVIFDCS
HEMAAGILKQALAMGMMTEYYHYIFTTLDLFALDVEPYRYSGVNMTGFRILNTENTQVSSIIEKWSMERLQAPPKPD
SGLLDGFMTTDAALMYDAVHVVSVAVQQFPQMTVSSLQCNRHKPWRFGTRFMSLIKEAHWEGLTGRITFNKTNGLRT
DFDLDVISLKEEGLEKIGTWDPASGLNMTESQKGKPANITDSLSNRSLIVTTILEEPYVLFKKSDKPLYGNDRFEGY
CIDLLRELSTILGFTYEIRLVEDGKYGAQDDANGQWNGMVRELIDHKADLAVAPLAITYVREKVIDFSKPFMTLGIS
ILYRKPNGTNPGVFSFLNPLSPDIWMYILLAYLGVSCVLFVIARFSPYEWYNPHPCNPDSDVVENNFTLLNSFWFGV
GALMQQGSELMPKALSTRIVGGIWWFFTLIIISSYTANLAAFLTVERMESPIDSADDLAKQTKIEYGAVEDGATMTF
FKKSKISTYDKMWAFMSSRRQSVLVKSNEEGIQRVLTSDYAFLMESTTIEFVTQRNCNLTQIGGLIDSKGYGVGTPM
GSPYRDKITIAILQLQEEGKLHMMKEKWWRGNGCPEEESKEASALGVQNIGGIFIVLAAGLVLSVFVAVGEFLYKSK
KNAQLEKRSFCSAMVEELRMSLKCQRRLKHKPQAPVIVKTEEVINMHTFNDRRLPGKETMA

>GluK3
MTAPWRRLRSLVWEYWAGLLVCAFWIPDSRGMPHVIRIGGIFEYADGPNAQVMNAEEHAF
RFSANIINRNRTLLPNTTLTYDIQRIHFHDSFEATKKACDQLALGVVAIFGPSQGSCTNA
VQSICNALEVPHIQLRWKHHPLDNKDTFYVNLYPDYASLSHAILDLVQYLKWRSATVVYD
DSTGLIRLQELIMAPSRYNIRLKIRQLPIDSDDSRPLLKEMKRGREFRIIFDCSHTMAAQ
ILKQAMAMGMMTEYYHFIFTTLDLYALDLEPYRYSGVNLTGFRILNVDNPHVSAIVEKWS
MERLQAAPRSESGLLDGVMMTDAALLYDAVHIVSVCYQRAPQMTVNSLQCHRHKAWRFGG
RFMNFIKEAQWEGLTGRIVFNKTSGLRTDFDLDIISLKEDGLEKVGVWSPADGLNITEVA
KGRGPNVTDSLTNRSLIVTTVLEEPFVMFRKSDRTLYGNDRFEGYCIDLLKELAHILGFS
YEIRLVEDGKYGAQDDKGQWNGMVKELIDHKADLAVAPLTITHVREKAIDFSKPFMTLGV
SILYRKPNGTNPSVFSFLNPLSPDIWMYVLLAYLGVSCVLFVIARFSPYEWYDAHPCNPG
SEVVENNFTLLNSFWFGMGSLMQQGSELMPKALSTRIIGGIWWFFTLIIISSYTANLAAF
LTVERMESPIDSADDLAKQTKIEYGAVKDGATMTFFKKSKISTFEKMWAFMSSKPSALVK
NNEEGIQRALTADYALLMESTTIEYVTQRNCNLTQIGGLIDSKGYGIGTPMGSPYRDKIT
IAILQLQEEDKLHIMKEKWWRGSGCPEEENKEASALGIQKIGGIFIVLAAGLVLSVLVAV
GEFVYKLRKTAEREQRSFCSTVADEIRFSLTCQRRVKHKPQPPMMVKTDAVINMHTFNDR
RLPGKDSMACSTSLAPVFP

>GluK4
MPRVSAPLVLLPAWLVMVACSPHSLRIAAILDDPMECSRGERLSITLAKNRINRAPERLG
KAKVEVDIFELLRDSEYETAETMCQILPKGVVAVLGPSSSPASSSIISNICGEKEVPHFK
VAPEEFVKFQFQRFTTLNLHPSNTDISVAVAGILNFFNCTTACLICAKAECLLNLEKLLR
QFLISKDTLSVRMLDDTRDPTPLLKEIRDDKTATIIIHANASMSHTILLKAAELGMVSAY
YTYIFTNLEFSLQRMDSLVDDRVNILGFSIFNQSHAFFQEFAQSLNQSWQENCDHVPFTG
PALSSALLFDAVYAVVTAVQELNRSQEIGVKPLSCGSAQIWQHGTSLMNYLRMVELEGLT
GHIEFNSKGQRSNYALKILQFTRNGFRQIGQWHVAEGLSMDSHLYASNISDTLFNTTLVV
TTILENPYLMLKGNHQEMEGNDRYEGFCVDMLKELAEILRFNYKIRLVGDGVYGVPEANG
TWTGMVGELIARKADLAVAGLTITAEREKVIDFSKPFMTLGISILYRVHMGRKPGYFSFL
DPFSPGVWLFMLLAYLAVSCVLFLVARLTPYEWYSPHPCAQGRCNLLVNQYSLGNSLWFP
VGGFMQQGSTIAPRALSTRCVSGVWWAFTLIIISSYTANLAAFLTVQRMDVPIESVDDLA
DQTAIEYGTIHGGSSMTFFQNSRYQTYQRMWNYMYSKQPSVFVKSTEEGIARVLNSNYAF
LLESTMNEYYRQRNCNLTQIGGLLDTKGYGIGMPVGSVFRDEFDLAILQLQENNRLEILK
RKWWEGGKCPKEEDHRAKGLGMENIGGIFVVLICGLIVAIFMAMLEFLWTLRHSEATEVS
VCQEMVTELRSIILCQDSIHPRRRRAAVPPPRPPIPEERRPRGTATLSNGKLCGAGEPDQ
LAQRLAQEAALVARGCTHIRVCPECRRFQGLRARPSPARSEESLEWEKTTNSSEPE

>GluK5
MPAELLLLLIVAFANPSCQVLSSLRMAAILDDQTVCGRGERLALALAREQINGIIEVPAKARVEVDIFEL
QRDSQYETTDTMCQILPKGVVSVLGPSSSPASASTVSHICGEKEIPHIKVGPEETPRLQYLRFASVSLYP
SNEDVSLAVSRILKSFNYPSASLICAKAECLLRLEELVRGFLISKETLSVRMLDDSRDPTPLLKEIRDDK
VSTIIIDANASISHLVLRKASELGMTSAFYKYILTTMDFPILHLDGIVEDSSNILGFSMFNTSHPFYPEF

Downloaded from www.annualreviews.org. Guest (guest) IP: 129.2.89.114 On: Mon, 07 Oct 2024 14:58:42

VRSLNMSWRENCEASTYPGPALSAALMFDAVHVVVSAVRELNRSQEIGVKPLACTSANIWPHGTSLMNYL
RMVEYDGLTGRVEFNSKGQRTNYTLRILEKSRQGHREIGVWYSNRTLAMNATTLDINLSQTLANKTLVVT
TILENPYVMRRPNFQALSGNERFEGFCVDMLRELAELLRFRYRLRLVEDGLYGAPEPNGSWTGMVGELIN
RKADLAVAAFTITAEREKVIDFSKPFMTLGISILYRVHMGRKPGYFSFLDPFSPAVWLFMLLAYLAVSCV
LFLAARLSPYEWYNPHPCLRARPHILENQYTLGNSLWFPVGGFMQQGSEIMPRALSTRCVSGVWWAFTLI
IISSYTANLAAFLTVQRMEVPVESADDLADQTNIEYGTIHAGSTMTFFQNSRYQTYQRMWNYMQSKQPSV
FVKSTEEGIARVLNSRYAFLLESTMNEYHRRLNCNLTQIGGLLDTKGYGIGMPLGSPFRDEITLAILQLQ
ENNRLEILKRKWWEGGRCPKEEDHRAKGLGMENIGGIFVVLICGLIIAVFVAVMEFIWSTRRSAESEEVS
VCQEMLQELRHAVSCRKTSRSRRRRRPGGPSRALLSLRAVREMRLSNGKLYSAGAGGDAGAHGGPQRLLD
DPGPPGGPRPQAPTPCTHVRVCQECRRIQALRASGAGAPPRGLGTPAEATSPPRPRPGPTGPRELTEHE

>GluN1
MSTMHLLTFALLFSCSFARAACDPKIVNIGAVLSTRKHEQMFREAVNQANKRHGSWKIQLNATSVTHKPNAIQMALS
VCEDLISSQVYAILVSHPPTPNDHFTPTPVSYTAGFYRIPVLGLTTRMSIYSDKSIHLSFLRTVPPYSHQSSVWFEM
MRVYNWNHIILLVSDDHEGRAAQKRLETLLEERESKAEKVLQFDPGTKNVTALLMEARELEARVIILSASEDDAATV
YRAAAMLNMTGSGYVWLVGEREISGNALRYAPDGIIGLQLINGKNESAHISDAVGVVAQAVHELLEKENITDPPRGC
VGNTNIWKTGPLFKRVLMSSKYADGVTGRVEFNEDGDRKFANYSIMNLQNRKLVQVGIYNGTHVIPNDRKIIWPGGE
TEKPRGYQMSTRLKIVTIHQEPFVYVKPTMSDGTCKEEFTVNGDPVKKVICTGPNDTSPGSPRHTVPQCCYGFCIDL
LIKLARTMNFTYEVHLVADGKFGTQERVNNSNKKEWNGMMGELLSGQADMIVAPLTINNERAQYIEFSKPFKYQGLT
ILVKKEIPRSTLDSFMQPFQSTLWLLVGLSVHVVAVMLYLLDRFSPFGRFKVNSEEEEEDALTLSSAMWFSWGVLLN
SGIGEGAPRSFSARILGMVWAGFAMIIVASYTANLAAFLVLDRPEERITGINDPRLRNPSDKFIYATVKQSSVDIYF
RRQVELSTMYRHMEKHNYESAAEAIQAVRDNKLHAFIWDSAVLEFEASQKCDLVTTGELFFRSGFGIGMRKDSPWKQ
NVSLSILKSHENGFMEDLDKTWVRYQECDSRSNAPATLTFENMAGVFMLVAGGIVAGIFLIFIEIAYKRHKDARRKQ
MQLAFAAVNVWRKNLQDRKSGRAEPDPKKKATFRAITSTLASSFKRRRSSKDTSTGGGRGALQNQKDTVLPRRAIER
EEGQLQLCSRHRES

>GluN2A
MGRLGYWTLLVLPALLVWRDPAQNAAAEKGPPALNIAVLLGHSHDVTERELRNLWGPEQATGLPLDVNVV
ALLMNRTDPKSLITHVCDLMSGARIHGLVFGDDTDQEAVAQMLDFISSQTFIPILGIHGGASMIMADKDP
TSTFFQFGASIQQQATVMLKIMQDYDWHVFSLVTTIFPGYRDFISFIKTTVDNSFVGWDMQNVITLDTSF
EDAKTQVQLKKIHSSVILLYCSKDEAVLILSEARSLGLTGYDFFWIVPSLVSGNTELIPKEFPSGLISVS
YDDWDYSLEARVRDGLGILTTAASSMLEKFSYIPEAKASCYGQAEKPETPLHTLHQFMVNVTWDGKDLSF
TEEGYQVHPRLVVIVLNKDREWEKVGKWENQTLSLRHAVWPRYKSFSDCEPDDNHLSIVTLEEAPFVIVE
DIDPLTETCVRNTVPCRKFVKINNSTNEGMNVKKCCKGFCIDILKKLSRTVKFTYDLYLVTNGKHGKKVN
NVWNGMIGEVVYQRAVMAVGSLTINEERSEVVDFSVPFVETGISVMVSRSNGTVSPSAFLEPFSASVWVM
MFVMLLIVSAIAVFVFEYFSPVGYNRNLAKGKAPHGPSFTIGKAIWLLWGLVFNNSVPVQNPKGTTSKIM
VSVWAFFAVIFLASYTANLAAFMIQEEFVDQVTGLSDKKFQRPHDYSPPFRFGTVPNGSTERNIRNNYPY
MHQYMTRFNQRGVEDALVSLKTGKLDAFIYDAAVLNYKAGRDEGCKLVTIGSGYIFASTGYGIALQKGSP
WKRQIDLALLQFVGDGEMEELETLWLTGICHNEKNEVMSSQLDIDNMAGVFYMLAAAMALSLITFIWEHL
FYWKLRFCFTGVCSDRPGLLFSISRGIYSCIHGVHIEEKKKSPDFNLTGSQSNMLKLLRSAKNISNMSNM
NSSRMDSPKRATDFIQRGSLIVDMVSDKGNLIYSDNRSFQGKDSIFGDNMNELQTFVANRHKDNLSNYVF
QGQHPLTLNESNPNTVEVAVSTESKGNSRPRQLWKKSMESLRQDSLNQNPVSQRDEKTAENRTHSLKSPR
YLPEEVAHSDISETSSRATCHREPDNNKNHKTKDNFKRSMASKYPKDCSDVDRTYMKTKASSPRDKIYTI
DGEKEPSFHLDPPQFVENITLPENVGFPDTYQDHNENFRKGDSTLPMNRNPLHNEDGLPNNDQYKLYAKH
FTLKDKGSPHSEGSDRYRQNSTHCRSCLSNLPTYSGHFTMRSPFKCDACLRMGNLYDIDEDQMLQETGNP
ATREEVYQQDWSQNNALQFQKNKLRINRQHSYDNILDKPREIDLSRPSRSISLKDRERLLEGNLYGSLFS
VPSSKLLGNKSSLFPQGLEDSKRSKSLLPDHASDNPFLHTYGDDQRLVIGRCPSDPYKHSLPSQAVNDSY
LRSSLRSTASYCSRDSRGHSDVYISEHVMPYAANKNTMYSTPRVLNSCSNRRVYKKMPSIESDV

>GluN2B
MKPSAECCSPKFWLVLAVLAVSGSKARSQKSPPSIGIAVILVGTSDEVAIKDAHEKDDFHHLSVVPRVEL
VAMNETDPKSIITRICDLMSDRKIQGVVFADDTDQEAIAQILDFISAQTLTPILGIHGGSSMIMADKDES
SMFFQFGPSIEQQASVMLNIMEEYDWYIFSIVTTYFPGYQDFVNKIRSTIENSFVGWELEEVLLLDMSLD
DGDSKIQNQLKKLQSPIILLYCTKEEATYIFEVANSVGLTGYGYTWIVPSLVAGDTDTVPSEFPTGLISV
SYDEWDYGLPARVRDGIAIITTAASDMLSEHSFIPEPKSSCYNTHEKRIYQSNMLNRYLINVTFEGRNLS
FSEDGYQMHPKLVIILLNKERKWERVGKWKDKSLQMKYYVWPRMCPETEEQEDDHLSIVTLEEAPFVIVE
SVDPLSGTCMRNTVPCQKRIISENKTDEEPGYIKKCCKGFCIDILKKISKSVKFTYDLYLVTNGKHGKKI

Downloaded from www.annualreviews.org. Guest (guest) IP: 129.2.89.114 On: Mon, 07 Oct 2024 14:58:42

NGTWNGMIGEVVMKRAYMAVGSLTINEERSEVVDFSVPFIETGISVMVSRSNGTVSPSAFLEPFSADVWV
MMFVMLLIVSAVAVFVFEYFSPVGYNRCLADGREPGGPSFTIGKAIWLLWGLVFNNSVPVQNPKGTTSKI
MVSVWAFFAVIFLASYTANLAAFMIQEEYVDQVSGLSDKKFQRPNDFSPPFRFGTVPNGSTERNIRNNYA
EMHAYMGKFNQRGVDDALLSLKTGKLDAFIYDAAVLNYMAGRDEGCKLVTIGSGKVFASTGYGIAIQKDS
GWKRQVDLAILQLFGDGEMEELEALWLTGICHNEKNEVMSSQLDIDNMAGVFYMLGAAMALSLITFICEH
LFYWQFRHCFMGVCSGKPGMVFSISRGIYSCIHGVAIEERQSVMNSPTATMNNTHSNILRLLRTAKNMAN
LSGVNGSPQSALDFIRRESSVYDISEHRRSFTHSDCKSYNNPPCEENLFSDYISEVERTFGNLQLKDSNV
YQDHYHHHHRPHSIGSTSSIDGLYDCDNPPFTTQPRSISKKPLDIGLPSSKHSQLSDLYGKFSFKSDRYS
GHDDLIRSDVSDISTHTVTYGNIEGNAAKRRKQQYKDSLKKRPASAKSRREFDEIELAYRRRPPRSPDHK
RYFRDKEGLRDFYLDQFRTKENSPHWEHVDLTDIYKERSDDFKRDSVSGGGPCTNRSHLKHGTGEKHGVV
GGVPAPWEKNLTNVDWEDRSGGNFCRSCPSKLHNYSSTVAGQNSGRQACIRCEACKKAGNLYDISEDNSL
QELDQPAAPVAVTSNASSTKYPQSPTNSKAQKKNRNKLRRQHSYDTFVDLQKEEAALAPRSVSLKDKGRF
MDGSPYAHMFEMPAGESSFANKSSVPTAGHHHNNPGSGYMLSKSLYPDRVTQNPFIPTFGDDQCLLHGSK
SYFFRQPTVAGASKTRPDFRALVTNKPVVVTLHGAVPGRFQKDICIGNQSNPCVPNNKNPRAFNGSSNGH
VYEKLSSIESDV

>GluN2C
MGGALGPALLLTSLLGAWARLGAGQGEQAVTVAVVFGSSGPLQTQARTRLTSQNFLDLPLEIQPLTVGVN
NTNPSSILTQICGLLGAARVHGIVFEDNVDTEAVAQLLDFVSSQTHVPILSISGGSAVVLTPKEPGSAFL
QLGVSLEQQLQVLFKVLEEYDWSAFAVITSLHPGHALFLEGVRAVADASYLSWRLLDVLTLELGPGGPRA
RTQRLLRQVDAPVLVAYCSREEAEVLFAEAAQAGLVGPGHVWLVPNLALGSTDAPPAAFPVGLISVVTES
WRLSLRQKVRDGVAILALGAHSYRRQYGTLPAPAGDCRSHPGPVSPAREAFYRHLLNVTWEGRDFSFSPG
GYLVRPTMVVIALNRHRLWEMVGRWDHGVLYMKYPVWPRYSTSLQPVVDSRHLTVATLEERPFVIVESPD
PGTGGCVPNTVPCRRQSNHTFSSGDLTPYTKLCCKGFCIDILKKLAKVVKFSYDLYLVTNGKHGKRVRGV
WNGMIGEVYYKRADMAIGSLTINEERSEIIDFSVPFVETGISVMVSRSNGTVSPSAFLEPYSPAVWVMMF
VMCLTVVAITVFMFEYFSPVSYNQNLTKGKKPGGPSFTIGKSVWLLWALVFNNSVPIENPRGTTSKIMVL
VWAFFAVIFLASYTANLAAFMIQEQYIDTVSGLSDKKFQRPQDQYPPFRFGTVPNGSTERNIRSNYRDMH
THMVKFNQRSVEDALTSLKMGKLDAFIYDAAVLNYMAGKDEGCKLVTIGSGKVFATTGYGIAMQKDSHWK
RAIDLALLQLLGDGETQKLETVWLSGICQNEKNEVMSSKLDIDNMAGVFYMLLVAMGLALLVFAWEHLVY
WKLRHSVPNSSQLDFLLAFSRGIYSCFNGVQSLPSPARPPSPDLTADSAQANVLKMLQAARDMVNTADVS
SSLDRATRTIENWGNNRRVPAPTASGPRSSTPGPPGQPSPSGWGPPGGGRTPLARRAPQPPARPATCGPP
LPDVSRPSCRHASDARWPVRVGHQGPHVSASERRALPERSLLPAHCHYSSFPRAERSGRPYLPLFPEPPE
PDDLPLLGPEQLARREAMLRAAWARGPRPRHASLPSSVAEAFTRSNPLPARCTGHACACPCPQSRPSCRH
LAQAQSLRLPSYPEACVEGVPAGVATWQPRQHVCLHAHTRLPFCWGTVCRHPPPCTSHSPWLIGTWEPPA
HRVRTLGLGTGYRDSGVLEEVSREACGTQGFPRSCTWRRVSSLESEV

>GluN2D
MRGAGGPRGPRGPAKMLLLLALACASPFPEEVPGPGAVGGGTGGARPLNVALVFSGPAYAAEAARLGPAV
AAAVRSPGLDVRPVALVLNGSDPRSLVLQLCDLLSGLRVHGVVFEDDSRAPAVAPILDFLSAQTSLPIVA
VHGGAALVLTPKEKGSTFLQLGSSTEQQLQVIFEVLEEYDWTSFVAVTTRAPGHRAFLSYIEVLTDGSLV
GWEHRGALTLDPGAGEAVLGAQLRSVSAQIRLLFCAREEAEPVFRAAEEAGLTGPGYVWFMVGPQLAGGG
GSGVPGEPLLLPGGSPLPAGLFAVRSAGWRDDLARRVAAGVAVVARGAQALLRDYGFLPELGHDCRTQNR
THRGESLHRYFMNITWDNRDYSFNEDGFLVNPSLVVISLTRDRTWEVVGSWEQQTLRLKYPLWSRYGRFL
QPVDDTQHLTVATLEERPFVIVEPADPISGTCIRDSVPCRSQLNRTHSPPPDAPRPEKRCCKGFCIDILK
RLAHTIGFSYDLYLVTNGKHGKKIDGVWNGMIGEVFYQRADMAIGSLTINEERSEIVDFSVPFVETGISV
MVARSNGTVSPSAFLEPYSPAVWVMMFVMCLTVVAVTVFIFEYLSPVGYNRSLATGKRPGGSTFTIGKSI
WLLWALVFNNSVPVENPRGTTSKIMVLVWAFFAVIFLASYTANLAAFMIQEEYVDTVSGLSDRKFQRPQE
QYPPLKFGTVPNGSTEKNIRSNYPDMHSYMVRYNQPRVEEALTQLKAGKLDAFIYDAAVLNYMARKDEGC
KLVTIGSGKVFATTGYGIALHKGSRWKRPIDLALLQFLGDDEIEMLERLWLSGICHNDKIEVMSSKLDID
NMAGVFYMLLVAMGLSLLVFAWEHLVYWRLRHCLGPTHRMDFLLAFSRGMYSCCSAEAAPPPAKPPPPPQ
PLPSPAYPAARPPPGPAPFVPRERAAADRWRRAKGTGPPGGAAIADGFHRYYGPIEPQGLGLGEARAAPR
GAAGRPLSPPTTQPPQKPPPSYFAIVREQEPTEPPAGAFPGFPSPPAPPAAAAAAVGPPLCRLAFEDESP
PAPSRWPRSDPESQPLLGGGAGGPSAGAPTAPPPRRAAPPPCAYLDLEPSPSDSEDSESLGGASLGGLEP
WWFADFPYPYAERLGPPPGRYWSVDKLGGWRAGSWDYLPPRGGPAWHCRHCASLELLPPPRHLSCSHDGL
DGGWWAPPPPPWAAGPPPRRRARCGCPRPHPHRPRASHRAPAAAPHHHRHRRAAGGWDFPPPAPTSRSLE
DLSSCPRAAPTRRLTGPSRHARRCPHAAHWGPPLPTASHRRHRGGDLGTRRGSAHFSSLESEV

Downloaded from www.annualreviews.org. Guest (guest) IP: 129.2.89.114 On: Mon, 07 Oct 2024 14:58:42

>GluN3A
MRRLSLWWLLSRVCLLLPPPCALVLAGVPSSSSHPQPCQILKRIGHAVRVGAVHLQPWTTAPRAASRAQE
GGRAGAQRDDPESGTWRPPAPSQGARWLGSALHGRGPPGSRKLGEGAGAETLWPRDALLFAVENLNRVEG
LLPYNLSLEVVMAIEAGLGDLPLMPFSSPSSPWSSDPFSFLQSVCHTVVVQGVSALLAFPQSQGEMMELD
LVSSVLHIPVLSIVRHEFPRESQNPLHLQLSLENSLSSDADVTVSILTMNNWYNFSLLLCQEDWNITDFL
LLTENNSKFHLESVINITANLSSTKDLLSFLQVQMDNIRNSTPTMVMFGCDMDSIRQIFEMSTQFGLSPP
ELHWVLGDSQNVEELRTEGLPLGLIAHGKTTQSVFEYYVQDAMELVARAVATATMIQPELALLPSTMNCM
DVKTTNLTSGQYLSRFLANTTFRGLSGSIKVKGSTIISSENNFFIWNLQHDPMGKPMWTRLGSWQGGRIV
MDSGIWPEQAQRHKTHFQHPNKLHLRVVTLIEHPFVFTREVDDEGLCPAGQLCLDPMTNDSSMLDRLFSS
LHSSNDTVPIKFKKCCYGYCIDLLEQLAEDMNFDFDLYIVGDGKYGAWKNGHWTGLVGDLLSGTANMAVT
SFSINTARSQVIDFTSPFFSTSLGILVRTRDTAAPIGAFMWPLHWTMWLGIFVALHITAIFLTLYEWKSP
FGMTPKGRNRNKVFSFSSALNVCYALLFGRTAAIKPPKCWTGRFLMNLWAIFCMFCLSTYTANLAAVMVG
EKIYEELSGIHDPKLHHPSQGFRFGTVRESSAEDYVRQSFPEMHEYMRRYNVPATPDGVQYLKNDPEKLD
AFIMDKALLDYEVSIDADCKLLTVGKPFAIEGYGIGLPPNSPLTSNISELISQYKSHGFMDVLHDKWYKV
VPCGKRSFAVTETLQMGIKHFSGLFVLLCIGFGLSILTTIGEHIVHRLLLPRIKNKSKLQYWLHTSQRFH
RALNTSFVEEKQPRSKTKRVEKSRWRRWTCKTEGDSELSLFPRSNLGPQQLMVWNTSNLSHDNQRKYIFN
DEEGQNQLGTQAHQDIPLPQRRRELPASLTTNGKADSLNVTRSSVIQELSELEKQIQVIRQELQLAVSRK
TELEEYQKTNRTCES

>GluN3B
MESVRTLWLSVALALAVGSRVVRGHPQPCRVPTRAGASVRLAALLPRAPAARARVLAALATPAPRLPHNL
SLELVAVASPTRDPASLARGLCQVLAPPGVVASIAFPEARPELRLLQFLAAATETPVVSVLRREVRTALG
APTPFHLQLDWASPLETILDVLVSLVRAHAWEDIALVLCRVRDPGSLVTLWTNHASQAPKFVLDLSRLDS
RNDSLRAGLALLGALEGGGTPVPAAVLLGCSTARAHEVLEAAPPGPQWLLGTPLPAEALPTTGLPPGVLA
LGETEQHSLEAVVHDMVELVAQALSSMALVHPERALLPAVVNCDDLKTGGSEATGRTLARFLGNTSFQGR
TGAVWVTGSSQVHVSRHFKVWSLRRDPLGAPAWATVGSWQDGQLDFQPGAAALRVPSPSGTQARPKLRVV
TLVEHPFVFTRESDEDGQCPAGQLCLDPGTNDSARLDALFAALVNGSVPRTLRRCCYGYCIDLLERLAED
LAFDFELYIVGDGKYGALRDGRWTGLVGDLLAGRAHMAVTSFSINSARSQVVDFTSPFFSTSLGIMVRTR
DTASPIGAFMWPLHWSMWVGVFAALHLTALFLTLYEWRSPYGLTPRGRNRGTVFSYSSALNLCYAILFGR
TVSSKTPKCPTGRFLMNLWAIFCLLVLSSYTANLAAVMVGDKTFEELSGIHDPKLHHPSQGFRFGTVWES
SAEAYIKASFPEMHAHMRRHSAPTTPHGVAMLTSDPPKLNAFIMDKSLLDYEVSIDADCKLLTVGKPFAI
EGYGIGLPQNSPLTSNLSEFISRYKSSGFIDLLHDKWYKMVPCGKRVFAVTETLQMGVYHFSGLFVLLCL
GLGSALLTSLGEHVFYRLVLPRIRRGNKLQYWLHTSQKIHRALNTGPPEGQQERAEQERSGPKDELPATD
GAGRWRRVRRAVERERRVRFLLEPGEAGGDRPWLCSNGPGLQAELRELELRIEAARERLRSALLRRGELR
ALLGDGTRLRPLRLLHAAPAES

>PpGLR1
MWEIWRVLLAMQLMAVRCWFADAQPVSPPATIRIGALLAYNSTIGKAVRPALELAVRDINNSSLLGDSQLVLHLGNS
NCSAFQGAATASNLLKDEVVAILGPQTSVVSHFVSHMATVTQVPLVSFSATDPSLSEEQYFYFVRVTHSDDVQMQAI
AGIIQHYGWREVTALYIDDDFGNNGINSLLDALQSMGPNTVRKSNLSPTITSEEISTLLTKLSEMESRVFVVHVEPK
LGRELFIMAQRLQMMSQGYVWIVTEAMTSVMNDLSTDPKFSQALQGVIGTRSHIPGSSLLQDYKDRWVELHGNDSSV
GPAQMNNVYAWYAYDAMWTVANGIRIFLDAGGATTFVDPPARPSDAGGESELASLKVFRDGKLLLDSILDQQFTGLT
GPVQLDERNDLMGSSFDVVNMVGEGLRVVGYWSNATGCLPFAPALNTTSMLNENSSQSQLQTVIWPGGGVDVPKGWV
VPKIGRPLVIGVPNRVGYKEFVESSVDSNNRTAFRGFCIDVFQQALSNLPYAVSYYFTSFGDGNSTPSYDALVDEIA
EKKFDAVVGDVTITTKRSMSVDFTQPFTTSGLVVVVPVKQSNANYAWAFLRPFTPLMWLTTGAFFFFTGLVVWFLEH
KKNRDFRGRPKKQVVTTLWFVFMTLFFSQNERVNSTLGRAVLVIWLFVVLIIISSYTASLTSFLTVQQLLPTIQGIS
SLVSSNVPIGYQTGSFVRDYLLQLNVAPDRLVALNTLDEYTAALTKGAGRGGVGAIVDELPYVQSFLSTECAFTIAG
QEFTKSGWGFAFPKGSQLAIDFSTAILKLAENGELQRIHDLWVNTNTCSNRNVQTDSMELGVNTFWGLFLITGLASL
FCCLVYWTRMIIRHRRVFRERKLDGSERQMSRLEASKSFMKSLVTFIEEEETPTKRIRSSQRRKKSKEWLSSPEQIS
PSSSMPNDSKRVSRSPERSNDGSVSKKLEIQSTMVVSENGTMPPEHNESPATDATRDIREEQDSPNYHQTVSQPT

>PpGLR2
MCKPWLGLLAMQLVALCSCLDIVGGADAADAVFIGIGGLLAFNSTIGRAAKPALELAVKDVNDAKIFEKSQLVLHLG
NTNCSAFQGAAAAMNLLKQEVVAIVGPQTSVVSHFVSHMGTATQVPLVSFSATDPSLSEDQYPYFVRMTHSDNVQMA
AIAGIIQYYGWREVTALYTDDDFGNNGIDALGDALKAIGSSIVFKAGLDPKITSDGIGRVLTKLSQMESRVLVVHME
PNIGKELFVMAQWLQMMTQGYVWIVTEAMTSIMDYLDKDSDFRQALQGVVGTRSYIPSSPQLQDYKDRWLEYHSKDR

Downloaded from www.annualreviews.org. Guest (guest) IP: 129.2.89.114 On: Mon, 07 Oct 2024 14:58:42

SLGPAQMNNVYAWYAYDAVWMIAHAIKNFMQKGGATTFVQPPVYPVDAGGQSELADLKVFQDGRLFMNTILEYQQVS
GITGPLHVDERGDLIGSSFEIVNMGDNGLRMVGFWSNSTGCLAFAPDRTQIQTVIWPGGVTEVPRGWVVPKNGRPLL
IGVPNKIGYKEFVSSAVDSANRTSFHGFCIDVFQQALAYLPYSISYSFMKYGNGSSTPSYDALVNKVVEKDFDAVVG
DVTITTKRSTTVDFTQPYTTSGLAVVVPIRQGEGNHAWAFMRPFTPLMWVTTGTFFFFTGLVLWFLEHKKNRDFRGR
PKKQIVTTLWFIFSTLFFSQRERVNSTLGRAVLIIWLFVVLIIISSYTASLTSLLTVQQLLPTIQGISSLLTSNVPI
GYQTGSFVRDYLLQLNVAEERLVPLDTLAAYSAALTKGPNRGGVGAIVDELPYVQLFLSSECAFTIAGQEFTKSGWG
FVYIYTYRLDLHAFPKGSQLAIDFSTAILKLAENGELQRIHDLWLVSESCTKRNLAHDSTELGLNTFWGLFLITGCA
SVFCCLVYWTRMIIRHRKAIRERGARDGQVKMSRLQASKSFLKSLLTFIEEEEVSTTGRRSMRRKKTREWDESSPNQ
GSSSSIAPTLSSQRISKALESITAYATEEFCTPQLSEPMHGLDHGGGSTFTLLTSTTFKKSGLHGGY

>AtGLR1.1
MEILFSISILALLFSGVVAAPSDDDVFEEVRVGLVVDLSSIQGKILETSFNLALSDFYGINNGYRTRVSVLVRDSQG
DPIIALAAATDLLKNAKAEAIVGAQSLQEAKLLATISEKAKVPVISTFLPNTLSLKKYDNFIQWTHDTTSEAKGITS
LIQDFSCKSVVVIYEDADDWSESLQILVENFQDKGIYIARSASFAVSSSGENHMMNQLRKLKVSRASVFVVHMSEIL
VSRLFQCVEKLGLMEEAFAWILTARTMNYLEHFAITRSMQGVIGFKSYIPVSEEVKNFTSRLRKRMGDDTETEHSSV
IIGLRAHDIACILANAVEKFSVSGKVEASSNVSADLLDTIRHSRFKGLSGDIQISDNKFISETFEIVNIGREKQRRI
GLWSGGSFSQRRQIVWPGRSRKIPRHRVLAEKGEKKVLRVLVTAGNKVPHLVSVRPDPETGVNTVSGFCVEVFKTCI
APFNYELEFIPYRGNNDNLAYLLSTQRDKYDAAVGDITITSNRSLYVDFTLPYTDIGIGILTVKKKSQGMWTFFDPF
EKSLWLASGAFFVLTGIVVWLVERSVNPEFQGSWGQQLSMMLWFGFSTIVFAHREKLQKMSSRFLVIVWVFVVLILT
SSYSANLTSTKTISRMQLNHQMVFGGSTTSMTAKLGSINAVEAYAQLLRDGTLNHVINEIPYLSILIGNYPNDFVMT
DRVTNTNGFGFMFQKGSDLVPKVSREIAKLRSLGMLKDMEKKWFQKLDSLNVHSNTEEVASTNDDDEASKRFTFREL
RGLFIIAGAAHVLVLALHLFHTRQEVSRLCTKLQSF

>AtGLR1.2
MVRICIQTPILLSFLLVLLFFISNCFASSQNNDDDKRIRVRVGLVLDLGSVEGKIVRSSVSMALSDFYDNHNDYKTR
LSLLVRDSHGEPLLALDSVVDLLQTEGVQAIIGGNSLLEAKLLAELGEKARVPVISLNSPMSLSLSKYTHLIQATHN
SASEVKGITAFLHGFDWNSVALVLEDHDDWRESMHFMVDHFHENNVHVQSKVAFSVTSSEDSLMDRLRELKDLGTTV
FVVHLSEVIATRLFPCAEKLGMMGEGFAWILTSRSMSSFHDQFIDDLTKEAMEGVVGFKSYIPMSKELHNFTLRWRK
TLPVEEVTGSEITRLSISGVWAHDVAWSLASAAEVTRMPTVTSTLLEAIKESRFKGLSGNFQLDDMKLLSDKFEIVN
MIGSGERRVGFWNSNGSFSNRRQLSSTHDNLETIIWPGGSAQSPKGRSLRESGRKKLRVLVTSSNRFPRLMKVETDP
ITHEITIVEGFCIEVFQASIAPFNYEVEYIRWLNGTNYTKLAYALHSQKDKYDAAVGDITITSDRSMYVDFTLPYTE
MGLGIVAAKERSMWVFFQPLTPNLWITSAAFFVLTGIIVWLIERAENKEFQGSWPQQIGVVIWFGFSTLVYAHREKL
QHNLSRFVVTVWVFAVLILVTSYTATLTSMMTVQQIRFNANEDYVGHLSGSLIANAALTNSSLRAMRLLGLNTSEDY
AQALMNKSVSYIVSELPYLKILLGENPGHFLMVKTQSTTNGFGFMFQKGSELAPNVSREIAKLRTSERLNEMERRWF
DKQLPYTTDDTSNPITLYRFRGLFMITGVSFAFALAVLLILWLRERWEILVNSVNIYFSQRLRHFRILFTRTIHPSP
LGLDNPIGENAVQMAQRNRR

>AtGLR1.3
MERFCIQTQTLLSFLLVLLLFISRSFASTKNDDVDGGKRVQIRVGLVLDLGSLKGKIVKNSVSMALSYFYAIHNDYK
TRVSVSLRNSHGEPLLALASAVDLLKTEGVEAIIGGNSLLEAKLLGELGEKARVPMISLDSPFSLSLSKYTHLIQAT
HDSTSEAKGITSFINVFDWNSVALVYEDHDDWRESMQLLVEHFHENGVRVQSKVGFTVSSSEDFVMGRLQQLKDLGT
TVFVVHLSEVIATHLFPCARRLGLMGDGFVWILTAKTMNSFHENIDDFTKQAMEGVVGFKSYIPMSIELQNFTLRWR
KSLPVEEAELTRLSISGIWAHDIAFALARAAEVIRMPNVTSTLLEEITKTRFNGLSGDFQLNDKKLLSNKFEIINMI
GSSERRVGFLNSNGSFSNRRHLSSTHNKLETIIWPGGSAQSPKGTSLIDSDRKKLRVLVTSSNRFPRLMKVETDPVT
NELIVEGFCIEVFRASISPFNYEVEYIPWLNGSNYDNLAYALHSQKDKYDAAVGDITITSNRSTYVDFTLPFTEMGL
GIVAVKERSMWVFFQPLTPDLWITSAFFFVLTGVIVWLIERAENKEFQGSWPQQIGVVLWFGFSTLVYAHREKLKHN
LSRFVVTVWVFAVLILTASYTATLTSMMTVQQIRFNSNEDYVGHLSGSLIANVALTSSSLRAMRSLGLNSAADYAQA
LLNKTVSFVVDELPYLKVVLGENPTHFFMVKTQSTTNGFGFMFQKGFELVPNVSREISKLRTSEKLNEMEKRWFDNQ
LPYTTDDTSNPITLYRFRGLFIIIGVSFAFALAVLVILCLRDKWEILVDNLDLSQRLRHFRIHFVRSIHTSPLDDPI
GETAVQMAQQNRQ

>AtGLR1.4
MENCMIRNTGYFLTIFFLAFISFAVTCSGTNQKDNVDRLPVVYEDVRIGLVVDMGSMEGKLVTTSISMALSDFYHVN
NGYRTRVSVLSRDSHGDPLQALAAAMDLLQTEQVEALVGGQSLLEAKNLAELGEKTKVPVISSFQVPSSLSLAKYNY
FIQATHDTSSEAKGIAALFSNFDWRTAVLIYEDDDDWRESIQPLVGHFQQNAIHIEYKAEFSVSSNEECIMKQLRKF

Downloaded from www.annualreviews.org. Guest (guest) IP: 129.2.89.114 On: Mon, 07 Oct 2024 14:58:42

KASGIRIFVAHISERIANRLFPCARRLGMMEEGYAWILTARSMNNFQDTNYLAKEEMEGVIGFKSYIPLTEELHNFT
LRWKRSLRLEEVVTRMSVCSIWAHDIAWSLARAAEVAKLPGLSVYDLLEAIPESAKHKGLSGDIKFIDKKFISDKFE
IVNMIGRGERSVGLWNSGSFISNRRRRLSSTKALETIIWPGGSTRIPKIRSLKEKRHGKKKKLRVLVPAGNITPQIL
EVKTDFKTGVTAATGYCIDVFETSILPFNYEVEYIPWPGAINYKNYNDLVYTLYSQKDKYDAAVGDITITDNRSLYV
DFTLPFTDMGLAVVTAKDKSMWIIFKPLTLSLWLTIASFFILTGAIVWLIERHDNADFQGSCFQQIGTLLCFGFSTL
VFAHRERLQHNMSRFVVIVWIFAVLILTSNYTATLTSVMTVQQIRGLKSNENIGFFSASIAANVVNDNPTFQGPRYK
GLKTADDFTNALRNGTISFIVDEVPYVKLFVAKHPSEFVIVETESVTNGFGFAFQKGSPLVQKVSREIEKLRRTEKL
KAIENWWFQRQTTSATSEDTFHPLTVYTFRGLFMITGVSFAFALIVYLIPWNREQRQVVLKHFHRYVSHRFAREIRP
SPTTPNRQNENSVI

>AtGLR2.1
MKRENNLVLSLLFFVIVFLMQVGEAQNRITNVNVGIVNDIGTAYSNMTLLCINMSLSDFYSSHPETQTRLVTTVVDS
KNDVVTAAAAALDLITNKEVKAILGPWTSMQAQFMIEMGQKSQVPIVTYSATSPSLASIRSQYFFRATYDDSSQVHA
IKEIIKLFGWREVAPVYVDDTFGEGIMPRLTDVLQEINVRIPYRTVISPNATDDEISVELLRMMTLPTRVFVVHLVE
LLASRFFAKATEIGLMKQGYVWILTNTITDVLSIMNETEIETMQGVLGVKTYVPRSKELENFRSRWTKRFPISDLNV
YGLWAYDATTALALAIEEAGTSNLTFVKMDAKRNVSELQGLGVSQYGPKLLQTLSRVRFQGLAGDFQFINGELQPSV
FEIVNVNGQGGRTIGFWMKEYGLFKNVDQKPASKTTFSSWQDRLRPIIWPGDTTSVPKGWEIPTNGKRLQIGVPVNN
TFQQFVKATRDPITNSTIFSGFSIDYFEAVIQAIPYDISYDFIPFQDGGYDALVYQVYLGKYDAVVADTTISSNRSM
YVDFSLPYTPSGVGLVVPVKDSVRRSSTIFLMPLTLALWLISLLSFFIIGLVVWVLEHRVNPDFDGPGQYQLSTIFW
FSFSIMVFAPRERVLSFWARVVVIIWYFLVLVLTQSYTASLASLLTTQHLHPTVTNINSLLAKGESVGYQSSFILGR
LRDSGFSEASLVSYGSPEHCDALLSKGQAEGGVSAVLMEVPYVRIFLGQYCNKYKMVQTPFKVDGLGFVFPIGSPLV
ADISRAILKVEESNKANQLENAWFKPIDESCPDPLTNPDPNPSVSFRQLGFDSFWVLFLVAAIVCTMALLKFVYQFL
KENPNQRNLRVLWEKFNEPDQKSYIKDVTKCQCSSGQGMPKNGQEGANAVNNGN

>AtGLR2.2
MKNSKLFFRFLFLFFFFCLESSRGQDNGKTQVNIGVVSDVGTSYPDVAMLCINMSLADFYSSRPQFQTRLVVNVGDS
KNDVVGAATAAIDLIKNKQVKAILGPWTSMQAHFLIEIGQKSRVPVVSYSATSPSLTSLRSPYFFRATYEDSSQVHA
IKAIIKLFGWREVVPVYIDNTFGEGIMPRLTDSLQDINVRIPYRSVIPLNATDQDISVELLKMMNMPTRVFIVHMSS
SLASTVFIKAKELGLMKPGYVWILTNGVMDGLRSINETGIEAMEGVLGIKTYIPKSKDLETFRSRWKRRFPQMELNV
YGLWAYDATTALAMAIEDAGINNMTFSNVDTGKNVSELDGLGLSQFGPKLLQTVSTVQFKGLAGDFHFVSGQLQPSV
FEIVNMIGTGERSIGFWTEGNGLVKKLDQEPRSIGTLSTWPDHLKHIIWPGEAVSVPKGWEIPTNGKKLRIGVPKRI
GFTDLVKVTRDPITNSTVVKGFCIDFFEAVIQAMPYDVSYEFFPFEKPNGEPAGNHNDLVHQVYLGQFDAVVGDTTI
LANRSSFVDFTLPFMKSGVGLIVPLKDEVKRDKFSFLKPLSIELWLTTLVFFFLVGISVWTLEHRVNSDFRGPANYQ
ASTIFWFAFSTMVFAPRERVLSFGARSLVVTWYFVLLVLTQSYTASLASLLTSQQLNPTITSMSSLLHRGETVGYQR
TSFILGKLNETGFPQSSLVPFDTAEECDELLKKGPKNGGVAAAFLGTPYVRLFLGQYCNTYKMVEEPFNVDGFGFVF
PIGSPLVADVSRAILKVAESPKAVELEHAWFKKKEQSCPDPVTNPDSNPTVTAIQLGVGSFWFLFLVVFV
VCVLALGKFTFCFLWKTKGKDLWKEFLKRDTDSYINDIEKCLCSQEMPENSNKATNQTNYGMELRVRNIVQVNQTDP
DCL

>AtGLR2.3
MRTEKLFFCILLVFFFCLEFNRGQNNGKTLVDVGVVTDVDTSHSKVVMLCINMSISDFYSSNPQFETRLVVNVGDSK
SDVVGAAIAALDLIKNKQVKAILGPWTSMQAHFLIEIGQKSRVPIVSYSATSPILTSLRSPYFLRATYEDSFQVQPI
KAIIKLFGWREVVPVYIDNTFGEGIMPRLTDALQDINVRIPYRSVIAINATDHEISVELLKMMNMPTRVFLVHMYYD
LASRFFIKAKELGLMEPGYVWILTNGVIDDLSLINETAVEAMEGVLGIKTYIPKSPDLEKFRSRWRSLFPRVELSVY
GLWAYDATTALAVAIEEAGTNNMTFSKVVDTGRNVSELEALGLSQFGPKLLQTLLTVQFRGLAGEFRFFRGQLQPSV
FEIVNIINTGEKSIGFWKEGNGLVKKLDQQASSISALSTWKDHLKHIVWPGEADSVPKGWQIPTKGKKLRIGVPKRT
GYTDLVKVTRDPITNSTVVTGFCIDFFEAVIRELPYDVSYEFIPFEKPDGKTAGNYNDLVYQVYLGRYDAVVGDTTI
LVNRSSYVDFTFPFIKSGVGLIVEMTDPVKRDYILFMKPLSWKLWLTSFISFFLVGCTVWVLEYKRNPDFSGPPRFQ
ASTICWFAFSTMVFAPRERVFSFWARALVIAWYFLVLVLTQSYTASLASLLTSQKLNPTITSMSSLLEKGETVGYQR
TSFILGKLKERGFPQSSLVPFDTAEECDELLSKGPKKGGVSGAFLEIPYLRLFLGQFCNTYKMVEEPFNVDGFGFVF
PIGSPLVADVSRAILKVAESPKAMELERAWFKKKEQSCPDPITNPDPNPSFTSRQLDIDSFLFLFVGVLLVCVMALG
NFTYCFLAKDQVSYLDKVEMSPCSSSQQMPVKRKTQLNMSQVHDQDSL

>AtGLR2.4
MSSKYFQDVYIPRYLTKTKNKRKMKRHLNDVVLVFLVFIFGVKLGKGQNTTIQVINVGVVTDVGTTASNLSLLAINM
SLSDFYSSRPESRTRLLLNFADSRDDVVGAAAAALDLIKNKEVKAILGPRTTMQASFVIEVGQKSQVPIISFSATSP

Downloaded from www.annualreviews.org. Guest (guest) IP: 129.2.89.114 On: Mon, 07 Oct 2024 14:58:42

FLDSGRSPYFFRSTYDDSSQVQAISEIIKVFGWREVVPVYENNAFGEGIMPGLTDALQAINIRIPYRTVISPNATDD
EISVDLLKLMTKPTRVFVVHMNRFLASRVFSKARETGLMKQGYAWILTNGVIDHLVLMNGTDIEAMQGVIGIRTHFP
ISEELQTFRSRLAKAFPVSELNIYGLRAYDATTALAMAVEEAGTTNLTFSKMDGRNISDLEALSVSEYGPKLIRSLS
QIQFKGLSGDYHFVDGQLHASVFEIVNVIDGGGILVGFWTQDKGLVKDLSPSSGTTRTFSSWKNHLNPILWPGITLT
VPKGWEIPTNGKELQIGVPVGTFPQFVKVTTDPLTHETIVTGFCIDFFEAVIQAMPYDVSHRFIPFGDDDGKTNDTT
ILANRSSYVDFTLPYTTSGVGMVVPLKDNVARSSLIFFKPLTPGLWGMTLGSFFVVGFVVWILEHRVNSEFTGPPQY
QISTMFWFAFSIMVFAPRERVMSFTARVVVITCLSSLLTTQQLNPTETSIKNVLAKGGPVAYQRDSFVLGKLRESGF
PESRLVPFTSPEKCEELLNKGPSKGGVSAAFMEVPYVRVFLGQYCKKYKMVEVPFDVDGFGFVFPIGSPLVADVSRA
ILKVAESNKATQLETAWFKNIDKTCPDPMNNPDPNPTVSFRKLSLDSFLLLFVAAATVCTLALLKFVICFLIQNRII
LNDEFYRGKRMKEMWLKFMESDGESYISRVRSTCPQVLIQPREEDIDPING

>AtGLR2.5
MASRQGLSSTSETPNKLLLVLPLQKREVVAIIGPGTSMQAPFLINLGNQSKVPIISFSATSPLLDSLRSPYFIRATH
DDSSQVQAISAIIESFRWREVVPIYVDNEFGEGILPNLVDAFQEINVRIRYRSAISLHYSDDQIKKELYKLMTMPTR
VFIVHMLPDLGSRLFSIAKEIDMLSKGYVWIVTNGIADLMSIMGESSLVNMHGVLGVKTYFAKSKELLHLEARWQKR
FGGEELNNFACWAYDAATALAMSVEEIRHVNMSFNTTKEDTSRDDIGTDLDELGVALSGPKLLDALSTVSFKGVAGR
FQLKNGKLEATTFKIINIEESGERTVGFWKSKVGLVKSLRVDKVSHSSRRLRPIIWPGDTIFVPKGWEFPTNAKKLR
IAVPKKDGFNNFVEVTKDENTNVPTVTGFCIDVFNTVMSQMPYAVSYEYIPFDTPDGKPRGSYDEMVYNVFLGEFDG
AVGDTTILANRSHYVDFALPYSETGIVFLVPVKDGKEKGEWVFLKPLTKELWLVTAASFLYIGIMVWIFEYQADEEF
REQMIIDKISSVFYFSFSTLFFAHRRPSESFFTRVLVVVWCFVLLILTQSYTATLTSMLTVQELRPTVRHMDDLRKS
GVNIGYQTGSFTFERLKQMRFDESRLKTYNSPEEMRELFLHKSSNGGIDAAFDEVAYIKLFMAKYCSEYSIIEPTFK
ADGFGFAFPLGSPLVSDISRQILNITEGDAMKAIENKWFLGEKHCLDSTTSDSPIQLDHHSFEALFLIVFVVSVILL
LLMLASRGYQERQHNASPNLPNDQANAAQEEVNEEGNVGDHIVEVDTALAKVSIVKPKL

>AtGLR2.6
MSLFNHLLSRALPLWLLFFINFLVLLGKSQQEVLQVQVGIVLDTNATLAALSLRAINMSLSEFYNTHNGFKTRIVLN
IRDSKRTVVGAAASALYLIKKREVVAIIGPGNSMQAPFLINLGNQSQVPIISFSASSPVLDSLRSPYFIRATHDDSS
QVHAISAIIESFRWREVVPIYADNEFGEGILPYLVDAFQEINVRIRYRSAISVHSTDDLVKKELYKLMTMPTRVFIV
HMLPDLGSRLFSIAKEIGMMTKGYVWIVTNGIADQMSVMGESSLENMHGVLGVKTYFSRSKELMYLETRWRKRFGGE
ELNNFECWGYDTATALAMSIEEISSNVNMSFSQTKRNTSRDDTGTDLDDLSFALSGPKLLQALATVSFKGVAGRFQL
KNGKLEATTFKIVNIEESGERTVGFWKSKVGLVKSLRVNQTGIKISHSSHRLRPIIWPGDTIFVPKGWEFPTNAKKL
RIAVPKKDGFNNFVEVTKDANTNAPTITGFCIDVFDTAMRQMPYAVPYEYIPFETPDGKPRGSYDEMVYHVFLGEFD
GAVGDTTILANRSTYVDFALPYSETGIVVVVPVKDEREKGKWVFLKPLTRELWFLTAASFLYIGIMSYTATLTSMLT
VQELRPTVRHMDDLRNSGVNIGYQTGSFTFERLKQMGYKESRLKTYDTPQEMHELFLKKSSNGGIDAAFDEVAYVKL
FMAKYCSKYTIIEPTFKADGFGFAFPLGSPLVPDLSRQILNITEGETMKAIENKWLLGEKHCLDSTTSDSPIRLDHH
SFEALFTIVFVVSMLLLLAMLVCRRYRQESKSGEINANNSPTDGNMRAPPNQPTDDNMRAPTSPPIDDQVLEPPGPA
LNEADDQDQLLNDEVNVGDRNEVDIIVEVDPTLVHRRNLITSKTIPTRRAALFSRIKSA

>AtGLR2.7
MKVMNPRKTNNTFMYYFVLFVCGFVLMEGCLGQNQTTEIKVGVVLDLHTSFSKLCLTSINISLSDFYKYHSDYTTRL
AIHIRDSMEDVVQASSAALDLIKNEQVSAIIGPRTSMQAEFMIRLADKSQVPTITFSATCPLLTSINSPYFVRATLD
DSSQVKAIAAIVKSFGWRNVVAIYVDNEFGEGILPLLTDALQDVQAFVVNRCLIPQEANDDQILKELYKLMTMQTRV
FVVHMPPTLGFRFFQKAREIGMMEEGYVWLLTDGVMNLLKSNERGSSLENMQGVLGVRSHIPKSKKLKNFRLRWEKM
FPKKGNDEEMNIFALRAYDSITALAMAVEKTNIKSLRYDHPIASGNNKTNLGTLGVSRYGPSLLKALSNVRFNGLAG
EFELINGQLESSVFDVINIIGSEERIIGLWRPSNGIVNAKSKNTTSVLGERLGPVIWPGKSKDVPKGWQIPTNGKML
RVGIPVKKGFLEFVDAKIDPISNAMTPTGYCIEIFEAVLKKLPYSVIPKYIAFLSPDENYDEMVYQVYTGAYDAVVG
DVTIVANRSLYVDFTLPYTESGVSMMVPLKDNKNTWVFLRPWSLDLWVTTACFFVFIGFIVWILEHRVNTDFRGPPH
HQIGTSFWFAFSTMNFAHREKVVSNLARFVVLVWCFVVLVLIQSYTANLTSFFTVKLLQPTVTNWKDLIKFNKNIGY
QRGTFVRELLKSQGFDESQLKPFGSAVECDELFSNGTITASFDEVAYIKVILSQNSSKYTMVEPSFKTAGFGFVFPK
KSPLTDDVSRAILNVTQGEEMQHIENKWFKKPNNCPDLNTSLSSNHLSLSSFWGLFLIAGIASFLALLIFVANFLYE
HKHTLFDDSENSFRGKLKFLVRNFDEKDIKSHMFKENAVHNVSSPITQGSSSPLTDQSTPLPRSPEQYRELELRRVS
SISSGELFTTQSEQVEDEESAIIQCEGE

>AtGLR2.8
MNPKKNNNTFLSYFVCLFLLLEVGLGQNQISEIKVGVVLDLNTTFSKICLTSINLALSDFYKDHPNYRTRLALHVRD
SMKDTVQASAAALDLIQNEQVSAIIGPIDSMQAKFMIKLANKTQVPTISFSATSPLLTSIKSDYFVRGTIDDSYQVK

Downloaded from www.annualreviews.org. Guest (guest) IP: 129.2.89.114 On: Mon, 07 Oct 2024 14:58:42

AIAAIFESFGWRSVVAIYVDNELGEGIMPYLFDALQDVQVDRSVIPSEANDDQILKELYKLMTRQTRVFVVHMASRL
ASRIFEKATEIGMMEEGYVWLMTNGMTHMMRHIHHGRSLNTIDGVLGVRSHVPKSKGLEDFRLRWKRNFKKENPWLR
DDLSIFGLWAYDSTTALAMAVEKTNISSFPYNNASGSSNNMTDLGTLHVSRYGPSLLEALSEIRFNGLAGRFNLIDR
QLESPKFEIINFVGNEERIVGFWTPSNGLVNVNSNKTTSFTGERFGPLIWPGKSTIVPKGWEIPTNGKKIKVGVPVK
KGFFNFVEVITDPITNITTPKGYAIDIFEAALKKLPYSVIPQYYRFESPDDDYDDLVYKVDNGTLDAVVGDVTITAY
RSLYADFTLPYTESGVSMMVPVRDNENKNTWVFLKPWGLDLWVTTACFFVLIGFVVWLFEHRVNTDFRGPPHHQIGT
SFWFSFSTMVFAHREKVVSNLARFVVVVWCFVVLVLTQSYTANLTSFLTVQRFQPAAINVKDLIKNGDYVGYQHGAF
VKDFLIKEGFNVSKLKPFGSSEECHALLSNGSISAAFDEVAYLRAILSQYCSKYAIVEPTFKTAGFGFAFPRNSPLT
GDVSKAILNVTQGDEMQHIENKWFMKQNDCPDPKTALSSNRLSLRSFWGLFLIAGIASFLALLIFVFLFLYENRHTL
CDDSEDSIWRKLTSLFRNFDEKDIKSHTFKSSAVHHVSSPMTQYIPSPSTLQIAPRPHSPSQDRAFELRRVSFTPNE
ERLTTQTIHFEDEESDIECVVEQ

>AtGLR2.9
MKTNNTFLSYFVCGFLLMGVGLGQNQTSEIKVGVVLDLNTTFSKICLTSIKMAVSDFYADHPNYLTRLTLHVRDSME
DTVQASAAALDLIKTEQVSAIIGPINSMQADFMIKLANKTQVPTITFSATSPLLTSIKSPYFVRATIDDSSQVRAIA
SIFKFFRWRRVVAIYVDNEFGEGFMPFLFDALQDVEVKRSVIPPEAIDDEIQKELRKLMERQARVFVVHMESSLALR
VFQIARDIGMMEEGYVWLMTNGMTHMMRHINNGRSLNTIEGVLGVRSHVPKSKELGDFRLRWKRTFEKENPSMRDDL
NVFALWAYDSITALAKAVEKANTKSLWYDNGSTLSKNRTDLGNVGVSLYGPSLQKAFSEVRFNGLAGEFKLIDGQLQ
SPKFEIINFVGNEERIIGFWTPRDGLMDATSSNKKTLGPVIWPGKSKIVPKGWEIPGKKLRVGVPMKKGFFDFVKVT
INPITNKKTPTGYAIEIFEAALKELPYLVIPEYVSFESPNNYNNLVYQVYDKTWDAVVGDITITANRSLYADFTLPF
TESGVSMMVPVRDNENKDTWVFLEPWSLELWVTTGCFFVFIGFVVWLFEHRVNTDFRGPPQYQIGTSLWFSFSTMVF
AHRENVVSNLARFVVVVWCFVVLVLTQSYTASLTSFLTVQSLQPTVTNVNDLIKNRDCVGYQGGAFVKDILLGLGFH
EDQLKPFDSAKDADDLLSKGKSKGIAAAFDEVAYLKAILSQSCSKYVMVEPTFKTGGFGFAFPKNSPLTGEFSRAIL
NLTQNNVTQQIEDRWFPKKNDCPDPMTALSSNRLNLSSFLGLFLIAGTAISFSLLVFVALFLYEHRHTLGDDSEDSL
WRKLKFLFKIFDEKDMNSHTFKNSAIHNISSPMTHKTPSPSTVQITPWPQSPSQNREFELRRVSFSPSEERFTTQPI
IHHEDGESDIECRVEQ

>AtGLR3.1
MSFPTFSFHFSKVSLCFLLKQLLLYGFFFSMNWVLLSFIIVLGGGLLLSEGASSSRPPVIKVGAIFGLNTMYGETAN
IAFKAAEEDVNSDPSFLGGSKLRILMNDAKRSGFLSIMGALQFMETDVVAIIGPQTSIMAHVLSHLANELTVPMLSF
TALDPTLSPLQFPFFVQTAPSDLFLMRAIAEMITYYGWSDVVALYNDDDNSRNGVTALGDELEERRCKISYKAVLPL
DVVITSPVEIIEELIKIRGMESRVIVVNTFPNTGKMIFKEAERLGMMEKGYVWIATTWLSSVLDSNLPLDTKLVNGV
LTLRLHTPDSRKKRDFAARWKNKLSNNKTIGLNVYGLYAYDTVWIIARAVKTLLEAGGNLSFSNDAKLGSLKGEALN
LSALSRFDQGSQLLDYIVHTKMSGLTGPVQFHPDRSMLQPSYDIINLVDDRVHQIGYWSNYSGLSIVPPESFYSKPP
NRSSSNQHLNSVTWPGGTSVTPRGWIFRNNGRRLRIGVPDRASFKDFVSRVNGSSNKVQGYCIDVFEAAVKLLSYPV
PHEFIFFGDGLTNPNYNELVNKVTTGVDFDAVVGDIAIVTKRTRIVDFTQPYIESGLVVVAPVTRLNENPWAFLRPF
TLPMWAVTASFFVIVGAAIWILEHRINDEFRGPPRRQIITILWFTFSTMFFSHRETTVSTLGRMVLLIWLFVVLIIT
SSYTASLTSILTVQQLNSPIKGVDTLISSTGRIGFQVGSFAENYMTDELNIASSRLVPLASPEEYANALQNGTVAAI
VDERPYIDLFLSDYCKFAIRGQEFTRCGWGFAFPRDSPLAVDMSTAILGLSETGELQKIHDRWLSKSNCSSPHGSQS
GDSEQLNVHSFWGMFLVVGIACLVALFIHFFKIIRDFCKDTPEVVVEEAIPSPKSSRLTKLQTFLAFVDEKEEETKR
RLKRKRNNDHSMNANSIISRTASRRPI

>AtGLR3.2
MFWVLVLLSFIVLIGDGMISEGAGLRPRYVDVGAIFSLGTLQGEVTNIAMKAAEEDVNSDPSFLGGSKLRITTYDAK
RNGFLTIMGALQFMETDAVAIIGPQTSIMAHVLSHLANELSVPMLSFTALDPSLSALQFPFFVQTAPSDLFLMRAIA
EMISYYGWSEVIALYNDDDNSRNGITALGDELEGRRCKISYKAVLPLDVVITSPREIINELVKIQGMESRVIIVNTF
PKTGKKIFEEAQKLGMMEKGYVWIATTWLTSLLDSVNPLPAKTAESLRGVLTLRIHTPNSKKKKDFVARWNKLSNGT
VGLNVYGLYAYDTVWIIARAVKRLLDSRANISFSSDPKLTSMKGGGSLNLGALSIFDQGSQFLDYIVNTNMTGVTGQ
IQFLPDRSMIQPSYDIINVVDDGFRQIGYWSNHSGLSIIPPESLYKKLSNRSSSNQHLNNVTWPGGTSETPRGWVFP
NNGRRLRIGVPDRASFKEFVSRLDGSNKVQGYAIDVFEAAVKLISYPVPHEFVLFGDGLKNPNFNEFVNNVTIGVFD
AVVGDIAIVTKRTRIVDFTQPYIESGLVVVAPVTKLNDTPWAFLRPFTPPMWAVTAAFFLIVGSVIWILEHRINDEF
RGPPRKQIVTILWFSFSTMFFSHRENTVSTLGRAVLLIWLFVVLIITSSYTASLTSILTVQQLNSPIRGVDTLISSS
GRVGFQVGSYAENYMIDELNIARSRLVPLGSPKEYAAALQNGTVAAIVDERPYVDLFLSEFCGFAIRGQEFTRSGWG
FAFPRDSPLAIDMSTAILGLSETGQLQKIHDKWLSRSNCSNLNGSVSDEDSEQLKLRSFWGLFLVCGISCFIALFIY
FFKIVRDFFRHGKYDEEATVPSPESSRSKSLQTFLAYFDEKEDESKRRMKRKRNDDLSLKPSRPI

Downloaded from www.annualreviews.org. Guest (guest) IP: 129.2.89.114 On: Mon, 07 Oct 2024 14:58:42

Downloaded from www.annualreviews.org. Guest (guest) IP: 129.2.89.114 On: Mon, 07 Oct 2024 14:58:42

>AtGLR3.3
MKQLWTFFFLSFLCSGLFRRTHSEKPKVVKIGSIFSFDSVIGKVAKIAIDEAVKDVNSNPDILSGTKFSVSMQNSNC
SGFMGMVEALRFMEKDIVGIIGPQCSVVAHMISHMANELRVPLLSFAVTDPVMSPLQFPYFIRTTQSDLYQMDAIAS
IVDFYGWKEVIAVFVDDDFGRNGVAALNDKLASRRLRITYKAGLHPDTAVNKNEIMNMLIKIMLLQPRIVVIHVYSE
LGFAVFKEAKYLGMMGNGYVWIATDWLSTNLDSSSPLPAERLETIQGVLVLRPHTPDSDFKREFFKRWRKMSGASLA
LNTYGLYAYDSVMLLARGLDKFFKDGGNISFSNHSMLNTLGKSGNLNLEAMTVFDGGEALLKDILGTRMVGLTGQLQ
FTPDRSRTRPAYDIINVAGTGVRQIGYWSNHSGLSTVLPELLYTKEKPNMSTSPKLKHVIWPGETFTKPRGWVFSNN
GKELKIGVPLRVSYKEFVSQIRGTENMFKGFCIDVFTAAVNLLPYAVPVKFIPYGNGKENPSYTHMVEMITTGNFDG
VVGDVAIVTNRTKIVDFTQPYAASGLVVVAPFKKLNSGAWAFLRPFNRLMWAVTGCCFLFVGIVVWILEHRTNDEFR
GPPKRQCVTILWFSFSTMFFAHRENTVSTLGRLVLIIWLFVVLIINSSYTASLTSILTVQQLSSPIKGIESLRERDD
PIGYQVGSFAESYLRNELNISESRLVPLGTPEAYAKALKDGPSKGGVAAIVDERPYVELFLSSNCAYRIVGQEFTKS
GWGFAFPRDSPLAIDLSTAILELAENGDLQRIHDKWLMKNACTLENAELESDRLHLKSFWGLFLICGVACLLALFLY
FVQIIRQLYKKPTDDAIARDQQQNHDSSSMRSTRLQRFLSLMDEKEESKHESKKRKIDGSMNDTSGSTRSRGFDRER
SFNSVNPLD

>AtGLR3.4
MGFLVMIREVSMAKAIRVVLLCVSVLWVVPKECACRSNFSRNSSSSSSSSLRPLRQRPSSVNVGALFTYDSFIGRAA
KPAVKAAMDDVNADQSVLKGIKLNIIFQDSNCSGFIGTMGALQLMENKVVAAIGPQSSGIAHMISYVANELHVPLLS
FGATDPTLSSLQFPYFLRTTQNDYFQMHAIADFLSYSGWRQVIAIFVDDECGRNGISVLGDVLAKKRSRISYKAAIT
PGADSSSIRDLLVSVNLMESRVFVVHVNPDSGLNVFSVAKSLGMMASGYVWIATDWLPTAMDSMEHVDSDTMDLLQG
VVAFRHYTIESSVKRQFMARWKNLRPNDGFNSYAMYAYDSVWLVARALDVFFRENNNITFSNDPNLHKTNGSTIQLS
ALSVFNEGEKFMKIILGMNHTGVTGPIQFDSDRNRVNPAYEVLNLEGTAPRTVGYWSNHSGLSVVHPETLYSRPPNT
STANQRLKGIIYPGEVTKPPRGWVFPNNGKPLRIGVPNRVSYTDYVSKDKNPPGVRGYCIDVFEAAIELLPYPVPRT
YILYGDGKRNPSYDNLVNEVVADNFDVAVGDITIVTNRTRYVDFTQPFIESGLVVVAPVKEAKSSPWSFLKPFTIEM
WAVTGGFFLFVGAMVWILEHRFNQEFRGPPRRQLITIFWFSFSTMFFSHRENTVSSLGRFVLIIWLFVVLIINSSYT
ASLTSILTIRQLTSRIEGIDSLVTSNEPIGVQDGTFARNYLINELNILPSRIVPLKDEEQYLSALQRGPNAGGVAAI
VDELPYIEVLLTNSNCKFRTVGQEFTRTGWGFAFQRDSPLAVDMSTAILQLSEEGELEKIHRKWLNYKHECSMQISN
SEDSQLSLKSFWGLFLICGITCFMALTVFFWRVFWQYQRLLPESADEERAGEVSEPSRSGRGSRAPSFKELIKVVDK
REAEIKEILKQKSSKKLKSTQSAAGTSQSQHGEIT

>*At*GLR3.5
MILSLEERPNLRLWRPLKTLMLTRVSSGAPSLILSSRTLIAVDLLAPWELMENKVVAAIGPQSSGIGHII
SHVANELHVPFLSFAATDPTLSSLQYPYFLRTTQNDYFQMNAITDFVSYFRWREVVAIFVDDEYGRNGIS
VLGDALAKKRAKISYKAAFPPGADNSSISDLLASVNLMESRIFVVHVNPDSGLNIFSVAKSLGMMGSGYV
WITTDWLLTALDSMEPLDPRALDLLQGVVAFRHYTPESDNKRQFKGRWKNLRFKESLKSDDGFNSYALYA
YDSVWLVARALDVFFSQGNTVTFSNDPSLRNTNDSGIKLSKLHIFNEGERFLQVILEMNYTGLTGQIEFN
SEKNRINPAYDILNIKSTGPLRVGYWSNHTGFSVAPPETLYSKPSNTSAKDQRLNEIIWPGEVIKPPRGW
VFPENGKPLKIGVPNRVSYKNYASKDKNPLGVKGFCIDIFEAAIQLLPYPVPRTYILYGDGKKNPSYDNL
ISEVAANIFDVAVGDVTIITNRTKFVDFTQPFIESGLVVVAPVKGAKSSPWSFLKPFTIEMWAVTGALFL
FVGAVIWILEHRFNEEFRGPPRRQIITVFWFSFSTMFFSHRENTVSTLGRFVLLVWLFVVLIINSSYTAS
LTSILTVQQLTSRIEGMDTLIASNEPIGVQDGTFAWKFLVNELNIAPSRIIPLKDEEEYLSALQRGPRGG
GVAAIVDELPYIKALLSNSNCKFRTVGQEFTRTGWGFAFQRDSPLAVDMSTAILQLAEEGKLEKIRKKWL
TYDHECTMQISDTENYQISVQSFWGLFLICGVVWFIALTLFCWKVFWQYQRLRPEESDEVQARSEEAGSS
RGKSLRAVSFKDLIKVVDKREAEIKEMLKEKSSKKLKDGQSSAENSQSKDHETPQ

>AtGLR3.6
MKWFLLMLIICNAVPLQGLTKIVSARPQVVNIGSVFTFNSLIGKVIKVAMDAAVEDVNASPSILNTTTLRIIMHDTK
YNGFMSIMEPLQFMESETVAIIGPQRSTTARVVAHVATELKIPILSFSATDPTMSPLQFPFFIRTSQNDLFQMAAIA
DIVQFYGWREVVAIYGDDDYGRNGVAALGDRLSEKRCRISYKAALPPAPTRENITDLLIKVALSESRIIVVHASFIW
GLELFNVARNLGMMSTGYVWIATNWLSTIIDTDSPLPLDTINNIQGVITLRLHTPNSIMKQNFVQRWHNLTHVGLST
YALYAYDTVWLLAQAIDDFFKKGGNVSFSKNPIISELGGGNLHLDALKVFDGGKIFLESILQVDRIGLTGRMKFTSD
RNLVNPAFDVLNVIGTGYTTIGYWFNHSGLSVMPADEMENTSFSGQKLHSVVWPGHSIKIPRGWVFSNNGRHLRIGV
PNRYRFEEVVSVKSNGMITGFCVDVFIAAINLLPYAVPFELVAFGNGHDNPSNSELVRLITTGVYDAGVGDITIITE

RTKMADFTQPYVESGLVVVAPVRKLGSSAMAFLRPFTPQMWLIAAASFLIVGAVIWCLEHKHNDEFRGPPRRQVITT
FWFSFSTLFFSHRETTTSNLGRIVLIIWLFVVLIINSSYTASLTSILTVHQLSSPIKGIETLQTNHDPIGYPQGSFV
RDYLIHELNIHVSRLVPLRSPEEYDKALRDGPGKGGVAAVVDERAYIELFLSNRCEFGIVGQEFTKNGWGFAFPRNS
PLAVDVSAAILQLSENGDMQRIRDKWLLRKACSLQGAEIEVDRLELKSFWGLFVVCGVACVLALAVYTVLMIRQFGQ
QCPEEAEGSIRRRSSPSARIHSFLSFVKEKEEDAKARSSRERQLEDISANGSSRCN

>AtGLR3.7
MGLGIDPSVAITALIVVILVVPMDCQRPQLVNIGAVFAFDSVIGRAAKVALEAAVSDVNNDKSFLKETELRLLMEDS
ACNVFRGSFGAFELLEKEVVAMIGPISSSVAHTISDIAKGLHFPLVSFAATDPTLSALQFPFFLRTTPNDAHQMSAL
VDLINFYGWKEVISVYSDDELGRNGVSALDDELYKKRSRISYKVPLSVHSDEKFLTNALNKSKSIGPRVYILHFGPD
PLLRIFDIAQKLQMMTHEYVWLATDWLSVTLDSLSDKGTLKRLEGVVGLRQHIPESVKMEHFTHKLQSNRSMNAYAL
HAYDTVWMIAHGIEELLNEGINITFSYSEKLLHARGTKLHLEKIKFFNSGELLLEKLLKVNFTGIAGQVQFGSGRNV
IGCDYEIINVNKTDVHTVGFWSKNGGFSVVAPKTRHSQKKTSFVSDEKLGDITWPGGGREKPRGWVIADSADPLKIV
VPRRVSFVEFVTEEKNSSHRIQGFCIDVFIEALKFVPYSVPYIFEPFGNGHSSPNYNHLIQMVTDGVYDAAVGDIAI
VPSRSKLVDFSQPYASTGLVVVIPANDDNATWIFLRPFTSRLWCVVLVSFLVIAVVIWILEHRINEDFRGPPRRQLS
TMLLFSFSTLFKRNQEDTISNLARLVMIVWLFLLMVLTASYTANLTSILTVQQLPSAITGIDSLRASEVPIGYQAGT
FTLEYLTYSLGMARSRLVPLDSTEEYEKALKLGPTNWGGVAAIVDELPYIELFLAERTGFKIVGEPFMHRGWGFAFK
RDSPLAIDMSTAILKLSETRKLQEIRKKWLCKTNCAGKSNWNPEPNQLHLKSFKGLYLVCIAITVSAFLVFVLRMIR
QFVRYRRMERTSSMPRASWSASPTLRLRELVFDFVEFVDEKEEAIKRMFRRSDDSNNNPSHVGEVQADTEVPRN

>RsGluR
MFWVLALLSCLIVVLSSGDGIVSEGRRPHDINVGAIFSLSTLYGQVADIALKAAEDDVNSDPTFLPGSKLRILMYDA
KRNGFLSIMKALQFMETDSVAIIGPQTSIMAHVLSYLANELNVPMCSFTALDPSLSPLQFPFFVQTAPSDLFLMRAI
AEMITYYGWSDVIALYNDDDNSRNGVTSLGDELEGRRCKISYKAVLPLDVVIKTPREIVRELVKIQKMESRVIIVNT
FPKTGKMVFEEARRLGMTGRGYVWIATTWMTSLLDSADPLSLPKVAESLRGVLTLRIHTPVSRKKRDFAARWNKLSN
GSVGLNVYGLYAYDTVWIIARAVKNLLDSRANIPFSGDSKLDHLKGGSLNLGALSMFDQGQQFLDYIVKTKMSGVTG
PVQFLPDRSMVQPAYDIINVVGGGLRQIGYWSNHSGLSVIPPELLFSKPSNRSSSNQHLENVTWPGGGSVTPRGWVF
PNNGRRLRIGVPNRASFKDFVSRVNGSSSSHIDGYSINVFEAAIKLLSYPVPHEFILFGDSLKNPNYNDLVNNVTTG
VFDAVVGDIAIVTKRTRIVDFTQPYIESGLVVVAPVTKLNDTPWAFLRPFTPPMWAVTAAFFLIVGSVIWILEHRIN
DEFRGPPRRQIVTILWFSFSTMFFSHRENTVSTLGRIVLLIWLFVVLIITSSYTASLTSILTVQQLNSPIKGVDTLI
SSSGRVGFQIGSYAENYMIDELNIARSRLVPLGSPKEYATALQNGTVAAIVDERPYVDLFLSEFCGFAIRGQEFTRS
GWGFAFPRDSPLAVDMSTAILGLSETGKLQKIHDKWLSKSNCSNLNGSESDDDPEQLKLRSFWGLFLLCGVACFIAL
LFIYFKIVRDFCNHHHNKPEEEEATVPSPEVSRSKTLQTFLAYFDEKEAESSRRLKRKRSDDLSLKSS

>ZmGLR1
MRIAFLVLLVLSLFLLPNGIGKSLAARPSVVNIRSILQLNSTTGGVSDVAIRAAVEDINLDPTVLNGTTL
QVQTRDTNCNDGFLGMVQALQFMETDVIAIIRPQCSPIAHIISYVANELQVPLMSFASDATLSSIQFPFF
VRTMPSDLYQMAAVAAVIDYYQWKIVTAIYVDDDYGRNGIAALDDELTARRCKISFKIGFRSNAKKSDLL
NLLVTVSNMESRVIILHTGSEPGLKLLSLANGLKMMGNSYVWIATDWLFAYLDANSSVSAETINAMQGVL
TIRPHTPKSKVKSNLVSKWSSLSKKYYHSDLRTSAYGFYVYDSVWTVARALDAFFEDGGRISFTNDSRLR
DETRGTLHLEAMSVFDMGNKLLNKIRNVNFTGVSGQVQFNAQFELIHPAYDIISIIGNGMRIIGFWSNYT
RLLSTVLPEDLYSKPPNTSLANQQLYDVIWPGETAQRPRGWAFPSNAKELKISVPNRFSFKDFVSKDNAT
GSMKGYCIDVFTQALALLPYPVTYRFIPFGNGTENPHYSQLVQMVVDNDFDAVIGDIVITMSRTKAIDFT
QPFIESGLIQMDTPSGVKGSIPACIKFSVKVPGEIPYNGIYYDPPEEVI

>ZmGLR2
MKSMLQVWGIFCCVCSCALGQNTSARPSVVNVGALFTFHSTIGRAAKVAIAAAVNDINRDPSVLQGTKLV
VQMQDTNYSGFISIVQALQFMEKDTIAIIGPQSSVVAHVISHVANELQVPLMSFAATDPTLTPLQYPFFV
RTVHSDQFQMASVAAIVDYYGWKMVTAVYIDDDYGRNGVSSLDDELAKRRLKILYKAAIRPGARKSEMAA
VLVKAAMMESRVFVLHARDDSGIDVFSLAYNLSMTSGGYVWIATDWLTACLDSAPRLGTGLLNTMQGVLT
LRQHTENTSRKKALVSRWSEVAKEEEEEDGGSLPNTYGLYAYDTVWMLAHGLDAFFNSGGNISFSPDPRL
RAVVGGALNLDALSVFDEGTLLLERIRNVSFMGATGPVKLDSDGNLIQPAYDIVNVVGSGLRTIGYWSNY
SGLSVVSPETLYKKPFNVSANQELHAAIWPGETVTRPRGWVFPNNGNELRIGVPDRVSYRQFISVDNQTG
TVGGFCIDVFAAAINLLQYPVTYRFVPFGNGRENPSYTELIGRILTNEFDAVVGDVAIVTNRTKVVDFTQ
PYVASGLVILTAVKTQSSDAWAFLQPFTIRMWSVTGVFFLVVGAVIWLLEHRINDDFRGPPAKQVITVFW

Downloaded from www.annualreviews.org. Guest (guest) IP: 129.2.89.114 On: Mon, 07 Oct 2024 14:58:42

FSFSTLFFAHREDTRSTLGRVVVIIWLFVVLIIQSSYTASLTSILTVQQLTSPIKGIDSLIASDEPIGFQ
VGSFAESYLVHELGVSPSRLKALGTPDEYKNALELGPRKGGVVAIVDERPYVEVFLVQHDKFAIVGAEFT
KSGWGFAFPRDSPLAVDLSTAILALSENGDLQRIHDKWLSNGPSPQSTTDLEPERLRVQSFSALFLLCGA
ACLAALAIHGCILARQYSLHVASQPPDAVATGADGAIRRSSRSSIRSFLSFADRREAQCPRGSCKDPAAA
LGGSSSSGVSSFTSSNASVSR

>ZmGLR3
MAGRARHPPFRLMFYLIGLLASLIPATSRAQPPETVTVGLIIDADSPVGRIASTTIPMALDDFYAALPNS
STRVQILQHDSGGDVVAAASAALQLMTTQGARAILGPQSSVESAFVADLATRAEVPVVSFSATSPSVSHS
EARFFVRAALSDAAQAEAIAALATYFGWRRVVPIYQDDDYGAAFVPFLVDALTAVRAEVPYRCALPSGAS
RDAVAAAMYRLESEQTRAFVVHARPALAELVFAAAVEAGMMAEGYAWVITDGLTGLLGSIHPPQGVIGLA
PHVPSTARLRDVRKRWAHKFMRQHRDADLAQAEMGCYALWAYDAAWAVASAAERLVSPGDQPSLQGLVGG
RSGPTDFSGLGKSMSGAKFLAAITSTTFEGLGGRFELINGELAVPAFRIVNIMDDARERGIGFWTRKGGL
HRQLGRRGIASNSGLLPVIWPADSTVVPIGWVQPTSGRKLQVAVLGRVDPGYWPIMHLDVDPATNRTVAG
GFVIEVFEAAVRLLPYALPFEYVLVGSMRYDTLVERVGKGEFDAAVADITITANRSQHVDFTLPYMSSGI
SMVVPMRDQRSKRAAWVFLKPLRYDLWLISFAFFVFTGFVVWAIEHRSNEEFRGPPSYQIGTLLYFGFST
LVFAHRENLKSNLSRFVVVVWVFVVLILQSSYTASLTSMLTVPQLEPAIGDFASLWPGTDKVGIMNNSFM
REAMTKTGFPQYRLRPYQATQSFHEALLNGTIGAIVDETLYLRLFLNSYCDNFTQIAQSNKTGGFGFAFP
KGSPYVGDLSRAILNLTESDELSSIERKWFGDADGCAAQGSPFTSASLSFDSFWGLFLITGATSLFCCAL
HLLLFVVANRRRICAARVPWRIRLRVVLKLLDDKDLSSHTFRTIKDGGGSVAGRSAGAHDAGASPAVARI
AAGSPLSVSNHTYDMSEWSFGAQSPAPAAAGEIELAGAGEAGEVASAPPTLAAARGSSDQSGTVVHQASN

>ZmGLR4
MIAGAAAAGQLLVVAGGARSSGSAALPSRPLALLLLLLLLLLCLVGAEQAAPTPGAAGERRRVDVDVGVI
LDRTTWLGNISWACMELALQDFYADDDDAGYSTRVRLHLRDAPAGPSAVDAASAGVDLLKNVHVQAIVGP
QTSTQAKFLAELGNKASVPIISFSANSPSRSPSQTPYFIRTAWNDSCQAEAIASLVQNYNWREVIPIIED
DDSNARFIPDLVDALGHVDTRVPYRCKIHPSAGEDEIKRAISGLKENWTSVFVVRMSYQLALKFFQLAKD
EGMMGQGYVWITAYGLTDIFNVVGYPALEVMEGVVGIEPYVPETVKLNKFRRRWREKYRSENPGTSINEP
ITSGLYAYDTVWAIALAAEQAGFVNSDFVLSETNNGSTDFDKISAYKAAEKFRGAFLKVNFTGISGQFVI
QDMQFQLVSTTYKIINIAGREKRAVGVWTPGLNMSKILEKKDGISTIIWPGGSENTPRGWLLPANKELKV
GVPVKPGFGSFIRSEDGIPKGFCIDVFEEVISNLTYKVPKHYVEFGNGEGESNGTYDELVYKVYLKEFDA
VVGDITILANRSLYVDYTLPYTESGVRMLVPVRDQRQKTAWTFLEPLTADLWFGTGSFVVFTGFIVWFIE
HRTNQEFRGPPASQIGSVFYFSFSTLVFAHRERIVNNLSRIAVVVWLFVVLILQQSYTASLSSILTVEQL
QPTVTNLDEVIRRGDYVGYLNDSFMPNLLKRLKINETKMRAFSSPEEYNDALSTGKVAVIVDEIPYLKVF
LAKYCHNYTMVGPTYKFDGFGYAFPRGSPLTPEISRGILELASNGRMDELEKKLYGDTSCPDKDDSQTSS
SLTLHSFLGLFIITGASSLLALILHVIITLYDHRSNWINGSGQISWRELLAILVKIFHERDSANTPDEEV
PGMEDIDPATAESPWSMSNHVIENVDSDTDTGSTPEGEGTPGREVANQGPGPLSFAYMHSEGAME

>ZmGLR5
MGSAHFAALLLCACLFVSGTVAADQNGNLTRPAEVRIGALFTFGSVIGMAVRPAIELAVADVNADPSLLW
GTKLSVLMQDTNCSGFVGTIEALQLLAKDVVAVLGPQSSAVAHVISHAVNELHVPLISFAATDPTLSSLE
YPYFVRATQSDYYQMGAIAAIISQYEWKQVIAIYVDDDYGRGGITALGDALAKRKCKISYKAKLPPGAAK
TTIEDILMQVNDMESRVYVIHVNPDSGLNVFSAAKSLGMMSSGYVWIATDWLSAVIDSSVHGIPDVMELT
QGVLVLRQHIADSDIQHAFLSKWNNLTRNGSSYFMHAYDSVWLVAHAVERFLREGNAMSFSADPKLQAKK
GSSLQLDSLRIFNSGDKLLEKVWSANFSGVSGPVQFTLDRDLVHPAYDILNIGGTGLRTIGYWSNSSGLS
AVAPENLSSSARDSSANNVQLRGVIWPGQTSEKPRGWVFSHHGMPMRIGVPLRTSYKEFVMQDDGPDGVK
GFAVDVFKAAISLLPYPVSCKFVLFGDGLKNPSYSELVQKVSENYFDAAVGDIAIVTNRTRLVDFTQPYI
ESGLIIVAPARVIESNAWAFLKPFTFQMWCVLVVIFLFVGAVVWILEHRSNTEFRGPPSQQIMTVCWFSF
STMFFAHRENTVSALGRFVLLIWLFAVLIINSSYTANLTSLLTVQELTSGIQGLDSLISSSSAIGYQVGS
FSRNYLVDELSIAESRLVALNSPSDYARALELGSGNGGVAAIIDELPYVEIFLSKYCKFKTVGQVFTKSG
WGFAFPRDSPLAEDLSTAILALSENGKLQKMHDEWLSGTECSADNGAGPSNSLSLSSFWGLFLICGLACF
LALVIFFLRIFCQYSRYSNQVEAQFAEPRVLNRPARLTTIKSLISFVDKKEEEVKNALKKRPNGTGQQHP
STPNAEEQPTLPP

>ZmGLR6
MGRRLLRHHSPSSSSSSLRRRGGLVLAAALLAWCWCQASLPVGAAQAQAQPAPVRVGVILNLTSAVGQRR

Downloaded from www.annualreviews.org. Guest (guest) IP: 129.2.89.114 On: Mon, 07 Oct 2024 14:58:42

KVGIEMAVGDYYAARPAASRTRVVLSFRDSAGDVVGAASAAVDLIKNEQVQAIIGPQTSAEAEFVAYLGN
RTHVPVLSSSATSPVLSPSQTPFFVRTAANDSYQAAPVAAALAALGWHAAAVVYEDSPYGSGILPALAGA
LQGVGARIAGRAAVPSGADDDRVDAVLYGLKAMPTRVFVVHMSALPAARLFRRARRAGMMTEDYAWVATD
GVGGVVDALGPDDIGAMDGVVSLRPFVRVTERVSNFSARFRARLRREHPSADIYPHDPTVVMLWSYDTAW
AIAAAAGVSSPTFQTSQQSAGVTDLDRLRVSATGAALLKAVRETTFRGLAGNFTLVDGQLQPPAYEFVNI
VGKSSRAVGFWTPEAGITQTLGAEAAKKGLKKILWPGDSTSVPRGWVVSPNGRKLRVAVPVKHGFKEFVD
VAGESTTGGHANVTGYCIEVFDAVMSKMPYPVSYEYVPFPYSSSSYDSLVSLVPRQIADIVVGDVTITAS
RMGSADFSMPFTDSGWSMVVAVRTETSTSMWIFLRPLTTSLWLASLAFFCFTGFVVWAIEHRINPSSAAR
RGSSSASSSTSPSPPSSSRTVRARESSSVISTDKLKEKLESNLSRFVVIIWVFVVLILTSSYTASLTSML
TVQKLQPAVTDVRELQRTGAYIGYQEGSFIKNSLKKLGFHEAKMRSYSTAGEYADALSKGPANGGVAAVF
DEIPYLKLFLSQYCDGYTMFGPVYKTDGFGFVFPIGSPLTPDVSRVLLTLAEGEKMAQIEKKWFGEPGAC
PSQGAAALGSSNLSFRSFGGLFLITGVVSSLMLLVYLATFIYQERGEVRPEPEEEGSGSSSSVRRLRAWL
RHFDQKDLKCATFRTGDHDSVRDGGNQTQRWAESVRYGRGGNGSVQAAIEEEAVAMGMSPLRFGTSTPSE
TMNAGSSPASEFGTSFEQRMQEAPHSVSVEMPRSTPS

>ZmGLR7
MRIAFLVLLVLSLFLLPNGIGKSLAARPSVVNIGSILRLNSTTGGVSDVAIRAAVEDINSDPTVLNGTTL
HVQTRDTNCDDGFLGMVQALQFMETDVIAIIGPQCSPIAHIISYVANELQVPLMSFASDATLSSIQFPFF
VRTMPSDLYQMAAVAAVIDYYQWKIVTAIYVDDDYGRNGIAALDDELTARRCKISFKIGFRSNAKKSDLL
NLLVTVSNMESRVIILHTGSEPGLKLLSLANGLNMMGNGYVWIATDWLSAYLDANSSVSAETINGMQGVL
TVRPHTPKSKVKSNLVSKWSSLSKKYNHSDLRTSAYGFYVYDSVWTVARALDAFFDDGGRISFTNDSRLR
DETGGTLHLEAMSVFDMGNKLLNKIRNVNFTGVSGQVQFNAQFELIHPAYDIISIIGNGMRTIGFWSNYT
RLLSTVLPEDLYSKPPNTSLANQQLYDVIWPGETAQRPRGWAFPSNAKELKIGVPNRFSFKDFVSKDNAT
GSMKGYCIDVFTQALALLPYPVTYRFIPFGNGTENPHYSQLVQMVADNDFDAAIGDIVITMSRTKAVDFT
QPFIESGLVILSPIKKHITNSWAFLQPFTLGMWCVTGLSFLVVGVVIWILEHRINNDFRGSPRQQIITIV
WFSFSTLFFAHRENTMSTLGRGVLLIWLFVVLIIQSSYTASLTSILTVQQLDTSIRGLDDLKNSDYPIGF
QVGSFVEEYMIKELNISQSRLKALGSPEEYAENLKLGPKKGGVMAIVDERPYVELFLSTYCKIAVAGSDF
TSGGWGFAFPRDSPLQIDLSTAILTLSENGELQRIHDKWLKTGDCSTDNAEFVDSNQLRLESFMGLFLIC
GAACVLALLIYLGITIRQYLRHEQPGPAISVDAGSSTSKRSLRKFISFADDKQPPPKKKRAMSLSRSSMP
TMPMSNRPGADIDVES

>ZmGLR8
MPMGALQVMLLRLVLLAASAATTAVTVTAAPPPSEVAVGALFTYDSTIGRAARLAIELAVDDVNADRTVL
AGTRLNLLAQDTNCSGFLGAVEALQLMERNVVAVIGPQSSGIGHVISHVVNELHVPLLSFAATDPTLSAS
EYPYFIRTTISDYFQMNAVASIVDYYQWKEVTAIFVDDDYGRGGVSALGDALAAKRARISYKAAIPPNSN
TDVINDVLFKANMMESRVMVVHVNPDTGTRIFSVANKLQMMATGYVWIVTDWLAAVLDSSTSRDRKEMSH
IQGLIVLRQHTPESVAKNKFISKWNNAARNRSITSGLNSYGFYAYDSVWAIARGVDQFLNNGQQINFSTD
PVLHDTNGSSLHLSTLKIFDGGEQMLQQLLLTNFTGLTGRVQFNSDRNLVHPAYDILNIGGSGSRLIGYW
SNYSGLSVAAPEILYQKPPDTSSIAQRLHNVVWPGDSTTTPKGWVFPNNGQPLRVGVPIKPSFKELVAAG
KGPDNVTGYCIDIFNAAIRLLPYPVPCQFIAIGDGRKNPNYDDIISMVAANSLDAAVGDFAIVRNRTKIA
EFTQPYIESGLVIVAPVKQATSSAWAFLKPFTLEMWCVTGALFIFVGIVVWILEHRSNEEFRGSPRRQLI
TIFCVLSLCEGKHLDGKLTAGQNTVSALGRFVLIIWLFVVLIINSSYTASLTSILTVQQLATGITGIDSL
ISSGLPIGYQAGKFTRNYLIEELNIPESRLVPLNTIQEYADALKRGPKDGGVVAIVDEMPYVEIFLSYHC
NFRIVGQEFTKEGWGFAFKRDFPLAADLSTAILQLSESGQLQRIHDEWFTRPSCSSDDSEVGATRLGLGS
FWGLFLVCALICLLALLVFFIRICWQYNKYSNSEAAGEPSAAAAAAAAVDAAAAAADAVERQRRPSRLGS
FKDIIQFVDKKEEEIKKTMKRRVSEKDNNQAAGSSEAHSVASA

>ZmGLR9
MMGGVAQLVVVAALLATAAAAPTAAAAGALPSEVAVGALFTYDSTIGRAAQLAIELAVDDVNADDKVLAW
TKLNLVSMDTNCSGFLGTIKALELMEKNVVAVIGPQSSGIGHAISQVVNELHVPLLSFAATDPTLSASEY
PYFLRTTTSDYFQMNAVASIVDYYQWKRVTAVYIDDEYGRGGVSALGDALALKRAQVSYKATIPPNSNTD
VIRDVLFKANMMESRVMVVHVNPDTGLRVFSAAKKLQMMASGYVWIVTDWLAAVLDSSASRNPKYMSNIQ
GVIVLRQHTPDSDAKNKFISRWNNVARNRSMTPGLNSYGFYAYDSVWAVARSVDQFLNAGNQINFSTDPR
LHDPNGTTLRLSTLKIFDGGDQMLQQLLLTNFTGLTGAVKFDSGGNLLHPAYDILNVGRSGTHLIGYWSN
YSGLSVAAPEILYQMSPNASTSTHQLNSVVWPGDSTDIPRGWVFPNDGQPLRVGVPVKPSFKALVSGSTP
DSVRGYCIDVFKSAIKLLPYPVPYQFIPIGDGTKNPSYVSIVGMVASNTLDAAVGDFAIVRNGTRLAEYT
QPYIDSGLVIVAPVKHITSSAWAFLKPFTWEMWFITGALFILVGIVVWLLEHRSNPEFRGPPCNQVITIF

Downloaded from www.annualreviews.org. Guest (guest) IP: 129.2.89.114 On: Mon, 07 Oct 2024 14:58:42

WFSFSTMFFSHQENTRTALGRFVLIIWMFVVLIITSSYTASLTSILTVQQLATGITGLDSLISSSLPIGY
QTGKFTKKYLMLNLNVPESRLVQLNTIEEYADALNRGPKNGGVAAIIDEKPYIDIFLSHYCNFKIVGQQF
TREGWGFAFQKDSPLAADMSTAILQLSESGQLQSIHDEWFTQPSCATNDESNVGATRLGLGSFWGLFLIC
ALICLFAVVVFFIRVCWQYKQYSNSEDADESNEAGADGAGKRQRKLSRLGSFQEILKFFDMKEEEVMKSS
MKRRPGEKDNHAAGF

>ZmGLR10
MGAAQLVILFLALATAVGAPPSEVAVGALFTYDSTIGRAAKLAIELAVDDVNSDGKVLPRTQLNLVPQDT
NCSGFLGTIEALQLMEKNVVALIGPQSSGIGHVISHVVNELHVPLLSFAATDPTLSASEYPYFIRTTISD
YFQMNAIASIVDYYQWKRVTAIFVDDDYGRGGVEALGDALALKRAKISYKAAIPPNSNTDVINDVLFKAN
MMESRVMIVHVNPDTGMRIFSVAKNLQMMASGYVWIVTDWLAAVLDSSANRGLKDMSHIQGLIVLRQHIP
ESEAKDKFISKWNNVAHNRNITSGLNSYGFYAYDSVWAVARAVDKFLSNGQQINFSTDPRLEDSNGSTLH
LSSLKIFDGGEQMLHQLLLTNFTGVTGPVQFDSEHNLVRPAYDILNVVGSGSRLIGYWSNYSGLSVAAPE
TLYQMPRNTSTSAQQLHDVVWPGDSTTKPKGWVFPNTGLPLRVGVPIKASFKELVSGRGDNMSGYCIEIF
NAAIKLLPYPVPCQFITIGDGTKNPSYIDIIRMVAANSLDAAVGDFAIVRNGTQLAEYTQPYIESGLVIV
APVKHVTSSAWAFLEPFTLEMWCVTVALFILVGIVVWLLEHRTNEEFRGSPRRQVITMLWFSFSTMFFSH
RENTVSTLGRFVLIIWLFVVLIITSSYTASLTSILTVQQLSTGITGIDSLVSSSLPIGYQAGKFTKRYLT
ENFNVPLSRLVPLNSIQEYADALNRGPKNGGVAAIVDEKPYIDIFLSNYCSFRIVGEEFTKEGWGFAFQR
DSPLAADLSTAILQLSESGQLQRIHDEWFSRSSCSSDDSEVGATRLGLGSFWGLFLVCALICLFALLVFF
IRVCRQYNQYSSSEAAGEPSTAAADPVLRQRRPSRLGSFKDLIQFVDKKEEEIKKTMKRRSGEKDSQAAG
FSYAQSVASA

>ZmGLR11
MKIAFLMSLVLSLFLLPNGICKSLVARPSVVNIGSILRLNSTIGGVSDVAIRAAVEDINSDPTVLNGTTL
HVETRDTNCNDGFLGMVQALQFMETDVIAIIGPQCSAIAHIISYVANELQVPLMSFASDATLSSIQFPFF
VRTMPSDLYQMAAVAAVVDYYQWKIVTAIYVDDDYGRNGIAALDDELTARRCKISYKTGFRSNAKKSELL
SLLVTVSNMESRVIILHTGSEPGLKLLSLANGLNMMGNGYVWIATDWLSAYLDANSSVSAETVNGMQGVL
TVRPHTPKSNMKRNLVSKWSSLSKKYNHSDLRTSAYGFYVYDSVWTVARALDAFFDDGGRISFTNDSRLR
DVTGGTLHLEAMSVFDMGNKLLDKIRNVNFAGVSGQVQFNAQFELIHPAYDIISIIGNGMRTIGFWSNYT
RLLSTTPPEDLYSKPPNTSLANQQLYDVIWPGETAQKPRGWAFPYNAKELKIGVPNRFSFKEFVSKDNGT
GSMKGYCIDVFTQALTLLPYPVTYRFIPFGNGTENPHFDQLAQMVADNDFDAAIGDIEITMSRTKIVDFT
QPFIESGLVILAPIKKHITNSWAFLQPFTLGMWCVTGLSFLVVGVVIWILEHRINDDFRGSPWQQLITIV
WFSFSTLFFAHREKTMSTLGRGVLIIWLFVVLIIQSSYTASLTSILTVQQLDTSIRGLDDLKHSDYPIGF
QVGSFVKEYMIKELNISQSRLKALGSPEEYAENLKLGPKKGGVMAIVDERPYVELFLSTYCKIAVAGQDF
TSGGWGFAFPRDSPLQVDLSTAILTLSEDGELQRIHDKWLKTGDCSSDNTEFVDSNQLRLESFMGLFLIC
GAACVLALLIYFGITLRQYLRHEQPGSAISADSGSSTSKRSLRKFISFVDERQRXTKEKRTMSLSRSSMP
TTPMSNRPGTGIDIES

>ZmGLR12
MHCHGTRNLAADSPSIRRNPVFQSQGHKQHQPIVQEMQMRFAATSASFVLLCLLAALLGHWPAAAAETVN
VGVILDLASAPGRRWRTSISMAVEDYYATHTNSTTRVDLHFRDSSGDAVAAASAAVDLIRNAKAQAIIDG
SRTAAAAEFVARIGDRAHVPVLAFSAAPATWTARFSVATAPGDSSQAAPIAGVLENFHWRSAVLLHEDSR
SGAGIVPALSDALRGAGATVAHRAAVPADASDDRLDAVLYRASAMTARVFVVHMPFPLALRLFHRAKGAG
MMSDGYVWIATSAVGDTGDGDGDALGSDDADAMQGVVSVRQYAPPTSEVSDFARRFKARFQLENNGSQDT
TEPTTSTLQAYDTAFAAAAAVEAAGISGSAFEPPTGGGTELDQLGVSATGEKLLKAVLDTTFEGLAGKFR
LLDGQPQTPAYEIVNFAADGLTTVGFWTTKSGVSQEFDAGSGEGLKKVSFPGAGESDTRIPDGWAFSPVE
RSLVIAVPVKHGFQQFVQVYNDTTSDRTMVSGYCIDVFEAAIKALPYPVYYQYAPYYGIGNASSSSYDQM
VELIPEEKADAVVGDVSITVVRMGDADFTMPYTESGWSMVVAVQAQTATGMFFFLKPLTPALWLVSLAAF
IFTGFVIWVIEHRINPEFRGTPLQQFGIIFHYAFSTLVFAHRENVVSNLSKFLMVIWVFAVLILTSSYTA
SLTSMLTVQKLRPAVTDVNDLIDNGDYVGYQEGSFVHGELLQMKFDPSRLRSYSTPAEYADALSKGSAGG
GVAAVFDEVPYLKVFLSQYCDGYTMSGPVYKGTGLGFAFARGSPMATEVSRAIVGLTEGDDMDLIERKWF
GVPGSCVDGVDADNASLTLWNFSGLFLITAVAATLVLLAYLVTFIYRERHEVRAAAEPGSGSVSLKRFRA
WLQHYDRKDMSAPHFRQQQGWSDSPSTNGGSSHGRKREGAEQEDATATRDFGGPRASPLSDHSRMDSVSR
SPLERKGSNEFRTPFEQRMGEAAAASGERRSSTPERKQSLKFAQDTEERKKLPLSP

>ZmGLR13

Downloaded from www.annualreviews.org. Guest (guest) IP: 129.2.89.114 On: Mon, 07 Oct 2024 14:58:42

MQQQAETMGYCSSYTSSSRPVVPSSSLRRLGLVLAAALLVWCQRVATAQQPVPVPVRVGVILNLTPSSPV
GQRRKLGIEMAVEDYYAARPGSRTRVALRFRDSAGDVVAATSAAVDLIKNEQVQAIIGPQTSAEADFVAY
LCNRTRVPMLSSSATSPALSPAQTPFFVRTAPNDSFQAAPVAAALATFGWRAAVVVYEDSPYGSGILPAL
AGALQGVGVRIMDRAAVPGDGRIDALLYRFKAMPTRVFVVHMNARLAARFFRRARLAGMMTEDYAWVATD
GVGGVVDALSPDDISAMEGVLSLRPFVQMTDRVGNFSARFRERLRREYPSADVYYPHDPTVVMLWTYDTA
WAIAAAAEAAGVSSPAFQTRQQSTAATDLDRLGVSATGATLLKAVRETTFRGLAGNFTLLDGQLQPPAYE
FVNVVGKSSRAVGFWTPDDGITQTLGADGAKGMRRTIFWPGDSTSAPRGWVVSPNGHKLRVAVPVKNGFK
EFVDVGGESATAEHPNITGYCIEVFDAVMSKMPYPVSYEYEPFPDSSESYENIVSLVPEQSADIVVGDVT
ITASRMSKVDFSMPFTDSGWSMVVAVRTETSTSMWIFLRPLTTSLWLASFAFFCFTGFVVWAIEHRINPE
FRGTRWQQFGLIFYFAFSTLVFSHKEKLESNLSRFVVIIWVFVVLILTSSYTASLTSMLTVQKLQPAATD
VTELQRTGAYIGYQEGSFIKQRLQKQGFDETKMRSYSTAEEYADALSSGRVAAVFDEIPYLKLFLSQYCD
GYTMYGPVYKADGFGFVFPTGSPLTPDVSRAVLTLAEGEEMAQIEKKWFGEPGACPRQSGGGAAAALGAS
NLSFRSFGGLFLITGVVSSLMLLVYLATFIYRERGEVRPEPEEGGLGSSSVRRLLAWMRHFDQRDLKCPT
FKTGNDDCVREGNHRRWVESVRNGRGGNGSVQAAIEEGAIAVGMTPLRFSTSTASETINADSSPASELGT
SFGQGILEAPHSVSVEMPGSTAS

>ZmGLR14
MASPLSSSSSSTSTSTSTSTLLRLICLCTALPVALQAAAARPPNVTVGALFTFDSVIGRSARTAIQLAVDDV
NRDPAVLRDTNLSVIFQDTKCSGFVGTIQALELMERHVVAVVGPQSSGIAHVVSHVANQLRVPLLSFAAT
DPALASSQYPYFVRATHDDRFQMAAVADVVAHHGWREVTAVYVDNDYGRGGVVALGDALEALRARVSYRA
AFPPGADRAALADLLVRANMMESRVFVVHASPDSGLDVFAAARSLDMMATGYVWIATDWLAAAIDAAGAG
AAAAAGNIQGVLMLRQYTPDSDAKASLVSRFAAKQYINAYGLFAYDSVWMAARAIDQFLDDNASGGNVSF
SADRNIRDANGSALGLSALRVFDQGEQLLRKVMLANFTGVTGSVRFQLDADGSGGATLINPAYEILNVGG
TGVRRVAYWSNYTRLSVEAPRLLADGGPPPNSNNTTTQQQQMYSVIWPGDTTAKPRGWVFPNNGKPLRI
GVPYRTTYKQFVSKDRSSPDGVSGYCVDVFNAAVALLPYPVPASFVLFGDGVKNPSYNDLVQRVADGFFD
AAVGDISIVTNRTRVVDFTQPYVESGLVIVSTVKAKNSNEWAFLKPFTPGMWAIIGAFFLFVGAVVWILE
HRFNPEFRGSPRRQMVTIFWFSFSTMFFAHRENTVSTLGRFVLIIWLFVVLIINSSYTASLTSILTVQQL
STGIQGLDSLLSSNDPIGYQVGSFARSYMMDELGVPASRLRELAIDGYAGSLQRGPSNGGVAAIVDELPY
VELFLSTNCQFRTVGQEFTKSGWGFAFQRDSPLAVDLSTAILTLSENGDLQRIHDKWLSPGTCASQSTDG
VGADRLNLGSFWGLFLICGVACFVALLIYFARILCQFCKYHGHGTTDGAGPFPVPERSLRRPARLTSIRD
LMSFVDMKEAEVKRAIRSRSDRRLDGSMGGRSYTSEGPSLSRPSSMSPV

>ZmGLR15
MASSAATTTTTLLRLLCLCTVLLVAMQAAAARPPNVTIGSLFAFDSVIGRSARSAIQLAVDDVNRDPTVL
NGTTLTVVFQDTKCSGFAGTIQAGLELMEKEVVAVVGPQSSVIAHVVSHVANQLRVPLLSFAATDPALAS
TQYPYFVRTVHDDRFQMAAIADVVSHFGWREVTAVYVDDDYGRGGVIALADALEATRARVSYRAAFPLGA
DRATLADILQRANFMESRVFIVHASPDSGLNVFAAARGLGMMVSGYTWIATDWLATAAIDAAGAASNSSN
IQGVLTLRQYTPDSDAKASLLSRLAAADPTSNNATASVNTYGLFAYDSVWMAAYAIDQFLGDAGGGNVSF
SADPTIRDANGSALGLSALRVFDQGEQLLGKVMLSNFTGVTGHVEFQFDAGVNSSGTLVNPAYEILNVGG
TGVRRVAYWSNYTRLSVDAPKQLGDGVPPPNSTSTTAQQQMSNVIWPGGTTATPRGWVFADNGKPLTIGV
PYRTSYKEFVSKDETSPDGVSGYCVDVFKAAVALLPYPVPVSFVLFGDGVENPSYNELVQKVADGYFDAA
VGDISIVTNRTRVVDFTQPYIDSGLMIVSTVKSSSSDEWAFLKPFTPELWATVVAFCIFVGAVVWILEHR
HNDEFRGPLKKQMVTIFWFSFSSMFFTQREDTVSVPGRFVVIMWLFVVLIITQSYTASLTSILTVQQLST
GIQGLSDLLASNDPIGYQVGSFAGSYMNKELGVAATRLRELDPDDYADSLQRGPRGGGVAAIVDELPYME
LFLSSNCQFQTVGQEFTKSGWGFAFPLDSPLAVDLSTAILTLSENGDLQRIHDNWLNTGTCDSQNNGVGG
AERLSLRNFGGLFLICGVACVIALLIHFVRILFQFCQYRRHGAADGAQEEDENDGDDDRDGDSDKEKSQR
RPARQTSIRDLMSFVDMEEAEVKRAIRSRSGKSMGRSRSDTSDAPSSSPV

>ZmGLR16
MELAARTTSFVVFFILSFAQRAPTSEAATLNVGVILNLQSLVGKMARTSILMAMEDFYAVHRNYTTKLVL
HIRDSSADSVQAASEAVDLLKNYNVRAIIGPQKSSEATFVANLGNKSQVPVISFTATSPTLTSGSMPYFL
RATPSDTAQVNCIAALIKGYGWREVVPIYEDTDYGRGIIPYLVDSLQEFGASVPYRSVISVSASSDQVEQ
ELYKLMTMQTRVYVVHMLSSIASTLFMKANELGMMSEMYAWVLTDGIANIIDSLNPSILDSMNGALGVKF
YVPKSKELDDFTPRWTKRFKQDYPNDPSAQLGIFGLWGYDTIWALAQAAEKVNMVDDMFQKQQDKKPSTC
FGTLGISTVGPKLIDAILHNTFRGLSGDFDLKKRQLQPSTFQIINIVGRSSQQIGFWTAKHGIIRTLDQN
GSKTTNANSMPELNPVIWPGKVYVVPKGWQLPTNGNKLRVGVTSSGYPEFMKAERDPITNATIATGYAID

Downloaded from www.annualreviews.org. Guest (guest) IP: 129.2.89.114 On: Mon, 07 Oct 2024 14:58:42

VFEEVLKGLPYAIPYEYVAFDFEGASYNDFVYQVHLGVYDVAIGDITIRYNRTSYVDFTLPYTESGVAMI
VQVKDDTNKNTWVFLKPLTTDLWLGSIAFFIYTGIVIWLLERRINNAELTGSFFRQLGIAIYFSFFADRE
RIDSILSRLVVIVWVFVLLVITSSYTANLSSILTVQQLQPTVTDVHELIREGEYVGYHNGSYVGNLLEVL
GFDRTKIRAYKTSEDFAMHSLKGAKMVVLLLSYMKFPTSSYFLQSIAKVTQWLDQFTNPKALALTSIKNK
SDPNRDEKELEIILQNINSQAFPKRSPMINDFSRRILSITEGDVIIQIENKWIGDQHVCQTDGAIASPSS
LNFRSFSGLFLVTGVASTSALFIALMMFLYKNKHKIRDSISRVQTRGGYGSAHANRQNQEREVDSNQAQS
MHVTVPNDLEDDTCQQEIEVSIEITSPVLDSHRGQALHSVAQ

>ZmGLR17
MKAGAAAGQLAAGARSSGSAALSFLPLAVLLLCLVGAEQAPALAAGAGDRRVDVGVILDRTTWLGNISWA
CMELALHDFYADASHANYSTRVKLHLRDTPAGPSAVDAASAGVDLLKNVHVQAIVGPQTSTQAKFLTELG
NKTSVPIISFSANSPSRSSSQTPYFIRTAWNDSCQAEAIASLVQKYNWREVIPIIEDDDSNTRFIPDLVD
ALGHVDTRVPYRCKIHPSAGEDEIKHAISSLKEYWTSVFVVRMSYQLALKFFQLAKDEGMMGQGFVWITA
YGLTDIFNVVGYPALDVMEGVVGIEPYVQETAKLNKFRQRWCEKYRSENPGTSINESITSGLYAYDTVWA
IALAAEKAGYVNSDFVLSETNNGSTDFNKISTSRAAKKFHAAFLEVNFTGITGQFVIQDMQFQLVSTTYK
IINVVGHERRAVGFWTPGLNISRILEKKDDISTIVWPGGSEKTPRGWLLPVNKVLKIGVPGKSGFSSFIR
SEDGIPKGFCIDIFEEVISKLPYKVPKHYVEFGNGKGESNGTYDELVYKVYLKVSPKSNDQFYVNCWQEF
DAVVGDITILANRSLYVDYTLPYTESGVRMLVPVWDRRQKTAWTFLEPLTADLWLRTGAFIVFTGFVVWF
IEHRTNQEFRGPPATQIGSLFYFSFSTFVFAHRERIVNNLSRITVVVWLFVVLILQQSYTASLSSILTVE
QLQPTVTNLDEVIRRGDYVGYLSDSFMPELLKRLKINETKMIAFSSPEEYNDALSTRKVAVIVDEIPYLK
VFLSKYCHNYTMVGPTYKFDGFGYAFPRGSPLTSEISRGILELASNGRMDELEKQLYGDTSCPDKDDSQT
SSSLTSRSFLGLFIITGTSSLLALISHVIATLYDHRSHWINGGSQISWRELLAVLFKIFHERDSSNTPDK
EEPGMEGIYPTAAETPCSMSTHVIKNADSGTDMVSTPDDQKKKLHAIESGS

>SlGLR1.1
MRSATTNTIFSLLVYFLLIHHATFSLAYNKNDVNDSIYDDCNINMIKWRIGAIINPTTRVGKEQKIAMEM
AVDDFNAQNSKCSQLGFNFAYYSHGPAASLATYLAKKKQVHAILGPLTHQEAALFSNFDDEAYKDIPIIS
LTPAATYSTILLTEPISLIHMSNDVKFQMQCFAALIGHFKWRKVIALYEISNSFSNLDFGLITHLSDSLK
LVDSSIEYHLAFPPLFSVSNSKSFIQEELEKLRIKNVKVFVVAQCSLHFGLVLFEVATEMGMMGKDYVWI
VSDNMASLLDSVEPSVLLNMQGVIGFKANVNVKTESFREFNVKFRRKYRLEYPEEEEGYPSPSAYALKAY
DATWATAKAMEKLSRSDSSELVKSILLSDFEGLSGKISFKNGMLYQKPTYRIINVIGKSYREVSFWSPEF
GFSEDLVEYNGMTLKIGNGLEGDLGSILWPGGKQTVPKGWTIGGLEKPLRIGVPARGAFNQFVKVKFNQE
RNETLIDGFSVHVFEAAVRKLPYYLPYVLVPFYGNYDEMVEGVSNKSLDAAVGDTEILPDRYELAEFSQP
YIDSGLVMVVTERPRPEKTNFIVIKAFKLKLWILLAMMSMSTGVVIWLNEYVNDNLDFSGSFPQLIGSML
WFSVTVLSFSQREVIRSNLSRLVLTTWLCVVVVVTACFTALLSSIMTVPRLEPSVVNVDYLLRTNAAVGC
NNKSFIIKYLVNLQFKPENIKEISSINDYPNAFEKGEISAAFFVVPHAKVFLAKFCKGYTKSGPVYKLGG
FGFVFPKGSPLAVDISEAVLKVSQSGEIRQLEEQMLISSNCSSSSAVEHDPGLGPELFSGPLLISGAICG
IVFLISIVRLVRKHWLYLSSIIANSANVVLRCASLVLTQCYTRIVGSRSVKDSNNVIEQRPNNQQNIEMT
EVF

>SlGLR1.2
MLIFILKAFLFMSLVTVKATANETIKIGAIIDLNSRIGKEQKTGINIAVENYNHDRRNNKQLITVHFRNT
SKDTIQDFFTAEELVERNHVKMIVIGMQTWEETALIADIGKRHQVPIISFVTASYTPELVQLRWPFLVQM
TTSSLDQINCTASVVSSYQWRKVIVIYEDDMYSDSSMLAVLTETLKGHGVEVEHQLILPQFSSLSDPREV
VRREVVKLLQKQSRVFIVLRSSVSTANHLFKEAKEIGLMGRDSAWILADSLADLLDSVDKAFISSIQGAL
GIKNHYAEATKSFRHFKGQFQKIFRSEYPTEDHSEPGIHALKAYDSITAFANAVNNLGAKSSNDSVLMKN
RILSSNFTGLTGNISFVNGVLSHPPTFRIVNIDGNRYNGLGFWSSMFGFSKVLEAENGELIGVNGSRVMK
FSMVKWPGELKRVPKGWAMPTDAKPLIIGVPGRTSFEKFVKVETVAETNEMKYTGFCIDLFKEVLKILEK
NYTLPYDFEAYDGSYPDLVQQVINGRYDAIVGDITILAERTKYIEFTQPFAESGLTMVVPVKFDKSKKAW
MFLKPFTGNMWVATGSVLVYTMLVVWFMEHQSNPEFRGRWKDQLGTAMWFTFSSLFFAHRENIKSNYTKT
VVVVWLFLVFVLTSSYTASLTSMLTVPRLEPSVKDIGWIKRTNATVGCDGDSFVKDYLRQVLELQNIKNI
SNQDDYPKELENGNIKAAFLEIPYQKIFLREHCNQYVVAGPNYRFGGLAFAFQKGSPLARDVSEAILTLT
QDGTLNRLEEHWFALSKNCDNVDPTGETESLTLGSFWGLYLVSGATSTLCLLFYVYHLFRKSRQLTGAFR
DNILHPSTDQSLWTKTAGIIRYNKNDKPIVTLRRVTSARAAGLGVDERADSRKWHLVSPSDAAQIYDGSS
QHPQLAVELGNSRSN

>SlGLR2.1

Downloaded from www.annualreviews.org. Guest (guest) IP: 129.2.89.114 On: Mon, 07 Oct 2024 14:58:42

Downloaded from www.annualreviews.org. Guest (guest) IP: 129.2.89.114 On: Mon, 07 Oct 2024 14:58:42

MQNPRCSFLILFIQLVSIISFCSYARQIRGEDNKTSAIEVDVGIILDLETNVGKVMNISILLALADYHAN
ASRGAIKIVPHFRDSKRNDVEAASSAINLLKDVQVQAIFGPQMSTQTDFVIDIGNRTKVPIISPATSPSL
SVKENPFFIRGALPSSSQTKAIAAIVRNYDWRQVVIIYEDSSYGTGIVPHLTDALLEINTLVSYRSVLSP
SANDDEILKELYNLNTKQTRVFIVHLQPYLASRLFLKAKEAGMMNSGYAWIITDVLTSLLDSVDNSVIES
SMQGVLGIKPYIPRSNELNNYTRRWRKRFRQEYPDMDPVQLNVYGLWAYDSITALTKAIAKVGTTIIPKF
KKADTRENLTDLDALGTSEFGSLLLDSMQNTTLETGLSGEFRIFDGELQLYTYEIVNIIGKGERSVGFWT
EKDGILHKLKINSKTAKSMNEQLAAIIWPGESTIVPRGWEIPTSGEKLKVGVPVKGGLEQFIKVEINAKT
QAVTVTGFIPDVFKEVIEHLPYAIPYEFIPFPIDSPTSQDYDNLVYKISSKEYDAVVGDVTILASRAKYV
DFTLPFSESGISAVVSVGNDDRKNAWIFLKPLKSELWITTGGFFIFIGFVVWVLEHRVNKEFRGPKHKQV
GMIFWFSFSTLVFAHRERVTSNFTRFVLIVWVFVVLVLTSSYTANLTSMLTVQQLQPSITDLNDLIKNGE
YVGYQEGSFVKDILKHMKFDSSKFRSYSTLEEYSDALSRGSKNGGVGAIVDELPYLRLFLNKYCRKYIMV
GPTYKAAGFGFAFPKGSPLVPDVSRAVLLVMEGEFMNNIIQKWFGNETECPKQDGMVIASSLTLDSFKGL
FLIAGVSAGSALLLFFLIFLYQNREILATDDSVWQKLCAIANAFDKEKDNPNSMSQKPSEGNEIQTATLF
AESEASTEILPNLSLQSPEIKISDGLGASPPPEGFSTTEPGTPVHENITRITEEI

>SlGLR2.2
MVVILHKHNCLLPEFFQMHNPRCHFLILFIQLISIISFCHYVRGGDNNTSAVKVDVGIILDLERDVGKVM
HISILLALEDYHANTSRGDIRIVAHIKDSKKNDVEATSAAIYLLKDVQVQAIFGPIMSTQTNFVIDLGNR
AKVPIMSPATNPLLTVKENPFFIRGALPSSSQTKAIAAIVKKFDWKEVVVIYEDSLFGTGIVPHLTDALL
EIGTSVSYRSVISPSANDDRILSELYKLQTMQTRVFIVHLRPKLAKRLFLKANKAGMMSSGYAWIITDVL
TSLLDSVDTSVIESSMQGVLGVKPYIPRSDQRNSYTRRWRKRFRQEYPDMDQIELNIFGLWAYDSITSLA
EAVEKLGTTAIPKSKKPDTRENLTDLDALGTSAVGSLLIDSMRNTELKQGLSGDFRIIDGELQPVPYQIV
NIIGKGEKNIGLWTKRDGISCELKMNGKTAAKCNNTQLGAIFWPGETTIVPKGWEMPTSGKKLRVGVPLK
GGLEQLIKVDRDPQTQAVTATGFCADVFKEVILSLPYALPYEFIPFPIQDPLTLPDYDDLVHKITSQEYD
AVVGDVTILASRSEYVDFTLPFIGSGISVVVPVRDDDRKNAWIFLKPLKSELWITTGSFFVFIGFVVWVL
EHRVNKEFRGPKRKQVGMIFWFSFSTLVFAHREKVTSNLTRFVLIVWVFVVLVLTSSYTASLTSMLTLQQ
LQPTITDLNDLIKNGEYVGYQEGSFVKDAFIKHMKFDSSKFRSYNKLEDFDDALSKGSKNGGVGAIVDEL
PYLRLFLNKYCRKYIMVGQTYRAAGFGFAFPKGSPLVPDVSRAVLKVMEGEFMNSVIQKWFGNETDCTQN
DETDITSDSLTLDSFKGLFLIAGVSAGSALLLFFLNFVYQNREILATDDSICKKLTAIAKVFDQEKDDSN
STSEEPSESNAPKLLAASEASPEILPDLPSQSPEIRISDELGASPDAEGFFTTETWNSSSETITGTIEER

>SlGLR2.3
MHNPRCKFLILFVQLVSIISFCDYVIRIRGEDSKHSAVKVDVGIILDLETEVGKVMHISILLALADYHSR
GAIRIVPHIRDSKKDDVEAASAAIYLLKDVQVQAIFGPQMSTQTDFVIDLGERVRVPIISPATSPSLSVK
ENHYFIRGALPSSSQTKAIAAIVKNYHWREVVVIYEESPYGTGILPYLTDALLEINAFVSYRSGISPSAN
DDQILRELYKLKTMQTRVFIVHTQENLASRLFLKAKEAGMMNSGYAWIITDVLTSLLDLVDTSVIESSMQ
GVLGIKSYVPRSNERDMFTKRWRKRFRQEYPDMDQVELNIFGLWAYDSITILAEALEKVGTTSIQKLRKP
DTRENITDLDALGTSEVGSLLIHSLTNTELKPGLSGDFHIVSRELQPSPYQIVNIIGKGEKIVGFWTEKD
GISHKLKTNGKTAITNNKQLGVIIWPGESTDVPRGWEIPTSGKKLRIGVPDKGGLEQFIKVVRDPKTQAV
SATGFGPDVFKEVILSLPYAVPYDFVPFPIAHSPTSQNYDDLVNKITSKEYDAVVGDVTILASRSEHVDF
TLPFSESSISAVVPVRNDDRKNAWIFLKPLKAELWIATGAFFVFIGFVVWVLEHRVNKEFRGPKRKQVGM
IFWFSFSTLVFAHKEKITSNLSRFVLIVWVFVVLVLTSSYTASLTSMLTVQQLQPTVTDLNDLIKNGEYV
GYQKGSFVKDVLTRMKFDSSKLRSYRTLEEYDDALSRGSKNGGVGAIVDELPYLRLFLNKNCRKYIMVGP
TYKAAGFGFAFPKGSPLVPDVSRAVLKVIEGDAMNEIIQKWFGNETECPKQDGMAIASSLTLDSFKGLFL
IAGVSAGSALLLFFLIFLYQNREILATDDSIRKKLCSIAEVFDSERDNSNSQSTKPSEGNESLTAVFAES
EASTEISPNLPLQIPEIGISHGLGESPATEGFSTTEPGTPVHETMSGKIEER

>SlGLR2.4
MRSSKCHFLIVLIQFVSIISFYHYMMPIKGEDNKTSVAKVDVGIILDLETDMGKVMHISILLALDDYHAT
ASGSAIRIVPHLRDSKKDDVEAASAAIYLLKDVQIQAIFGPQMSTQTDFVIDLGNRVKVPIISPATNPLL
TVKENPFFIRGALSSSSQTKAIAAIVKNFDWKEVVVIYEDSPFGTGIVPHLTDALLEISTSVSYRSVISP
SANDDQILSELYKLKTMQTRVFIVHLRPKLAKRLFLKANKAGMMSDGYAWIITDVLTSLLDSVDTSVIES
SMQGVLGVKPYVPRTNELINYTKRWRRRFRQEYPDMDIVGLNVFGLWAYDGITTLAKAVEKVGGSAIPKF
KKADNREYLTDLDALGTSELGSLLLNSMQNTALKTGLSGDFRIVDGELQPSPYEIVNIIGKAERNTGFWT
EKDGISCKLKTNGKTAAKCNNKELGNIFWPGESTIAPKGWEIPTSGKKLRVGVPDKEGLEQFLKVEIDSK
TQEVTVTGFCADVFKEVIESLPYALPYEFIPFQILDSPTSPDFDVLAYKLFSEKFDAMIGDITISANRSK
YVDFTLPFTESGFSAVVPVKDDDRKNAWIFVKPLKSELWVTTGAFFVFIGFVVWVLEHRVNKEFRGPKRH

QVGMIFWFSFSTLVFAHSKPLTQKERVTSNFTRFVLIVWVFVVLVLTSSYTASLTSMLTAQKIQPTITDL
NDLIKRGEYVGYQKGSFVRGVLKSMKFDSTKFRSYSTLEEYNDALSKGSKNGGVGAIVDELPYLRLFLNK
YCRKYIMVGPTYKTAGFGFAFPKGSPLVPDVSRAVLKVMEGEFMNNIIQKWFGNETDCPRIDGMSITSDS
LTLDSFKGLFVTAGVSAGSALLLFFLNFLYQNREILATDDSVWKKLSAIAKAFDEEKDNSNSMSENPSEG
NGSQTTTLLAESEASAEVPDLPLQSIDIRISDRLGASSPIAEGFSTTEHGTPVHEIVTATIEERLHRVIQ
EMS

>SlGLR2.5
MKNPRRHFLILFIQLVSIISFCHYVMPIRGENNNTSAVKVDLGIILDMETDVGKVMHTCILLAIEDYHAA
ASHTATRIVPHLRDSEKDDVEEASAAIYLLKDVQVQAIFGPQTDFVIDLGNRAKVPIISPATNPLLSVKE
NPFFIRGALPSSSQTKAIAAIVKNYGWRQVVIIHEDSSYGTGIVPHLTDALLENNTLVSYRSVISPSAND
DQILKELYNLNTKQTRVFIVHLQPYLASRLFLKAKEAGMMSTGYAWIITDVLTSLLDSVDPSVIESSMQG
VLGIKPYVPSTTELKNFTKRWRKRFRQVYPDIDGVELNVFGLWAYDSITSLAEAVAKVGITAIPKFKRED
TRKNLTDIDALGTSELGSLLIHSMQNITLKAGLSGDFRIADGELQPSPYQIVNIIGTGQRSVGFWTEKDS
ISYKLKMNGKIAKTDNKQLGPIIWPGESTIVPKGWDMSTSGKRLRVGVSVNGKLDEFIKVERDSKTQAIV
ATGLCLDFFKEIIESLPYAVSYVFIPFTMPDSRTSPDYDHLDNMEYDVVVGDVTILAGQSKYVNFALPFP
ESGISTVVPVKDDERKNIWIFLKPLKSELWITTGAFFVFIGFVVWVLEHRVNKEFRGPKHKQVGMMFWFS
FSTLVFAHRERVTSNLTRFVLIVWVFVVLVLTSSYTASLSSMLTVQQLQPANDLINNGGYVGYLKGSFVE
YFLMRMQFDRSKLRSYSTLEEYNDALLRGSKNGGVSAIIDELPNLTLFLNKYCRKYIMVGQTYKTDGFGL
AFSEASPLVPDVSRAVLKMKEGGQFAKRGIQKYSSNETDCSQSNGTSDSLTLDSFRGLFLIAGVSAGTAL
LIFFLIFLYQNREILATDDSIRKKISAIAKVFDEEKDISNSKSGKPGCNEESQTATVLLAASETSPEILP
NLPSQSLEIRISDGLGESPAHEGFSATEPATLVHETITETF

>SlGLR2.6
MVIFTHAALDLLKNVEVEAIIGPFSSMQADFIINLGQKSQVPIISFSATSPSISSARNQYFVRTTHNDSS
QVKPISSIIQSFGWRQIVPIYIENQFGEGIISFLADALEEINTRIPYRSVISEFATSDQIRSELLKLMNM
QTRVFIVHMPISLGSKLFATAKEIGMMSEGFVWIVTDAMANQLNSMNVSVIESMEGVIGVKPYAPKSKKV
EDFTQRWKMKFRKENPTMVDVELDIYGLWAYDSATALAMAVEKSRINGAFFRKPNVSGTSDKLTDWVKFE
RSKGNATDLEAFGVSRDGPKLLKAILNTTFKGLSGDFQLVDGQLQSPPYQIINLIGNGVKEIGFWTREHG
IVRKLNSRRGYSVSKDNFRSIIWPGDTTSVPKGWVIPTNGKKLKIGVPVKDGFTEFVKVTRDVTTNTTIV
TGYCIDVFDAVMEALPYYVPYEYVPFAAPNGKSAGDYNELVYQVFLGYDSYMLQRLTDEQNFDVVVGDTT
IVANRSQFVDFTLPYTESGVTMMVPIKDDNRDNTWVFLKPLTWELWLTSFCSFVFIGFVIWLLEHRVNED
FRGPFWHQVGMIFWFSFSTMVFAQKERIVSNLARFVLIIWFLVLLILTSSYTASLTSMLTVEKLQPTVKD
VKELLNSKDYVGYQPGSFVVGLLRKMNFDEDRLKAYNTPEECVELLAKGSSNGGIAAVFDEIPYVKLFLA
NYCLKFTTIGPTYKTDGFGFAFPIGSPLVPDVSRAVLNVTEGEKMVQIERAWFGESTCSDLSSSLSSNSL
GLDSFWGLFVVAVVAAVLALVIFLTKFIHEHWHIIGRSDLSLRERSRILARKFDTKDYSCHTFKKSELRD
VLAHSTHDLDCSRSPQGNLSLLPSPRTTGPPSPSNSSHTEQMIHFPGEERASPSRGENEAVNGQVEMV

>SlGLR3.1
MGRAVKKAMELAVSDINGDPSILNGTSLNLIMEDSECSVFKGSIGGRVTEKQVVAIIGPQSSAIAHMISF
ISNGLHVPLISYAATDPTLSSLQFPFFLRTTQSDQSQMEAVADIVYFYEWKEVIAIFLDDDYGRNGIAAL
NDALTNKMLKISYKLPLPINYDITDIMYVLNQSKSLGPRVFVVHINPDSQLRFFNAVHKLKMNGSNYVWL
MTDWFSTTLDSFSPKNRSLLSTLEGVVSLRPYIPQSAQKRAFLSRWRKLLQNELVHSGLTAYGLYAYDTV
WVVARSIDNLLQQGGNISFSLSNMLNGTTSDKLQLGKLKEFDGGGLLMNILSLTNFTGLTGKIHFSQDRN
LIGSGYEVINIVKQEIHIVGYWSNFSGLSVLPPKPLQNKETAVTNLNQNLKSVSWPGGKSETPRGWVIAN
DERPLRIGFPRRASFTEFVTLNASHNVQGYCIDLFYEARKLVPYDIPFTFVPFGSGLANPDYNAFVNMVA
TDVFDAAIGDIAIVTNRTRMVDFTQPYVSTGLVIVAPIDTSESSAWVFLKPFTLEMWGVTALSFLIIAVV
IWILEHRVNEDFRGPPKRQITTMFLFSFSTLFKTNQENTVSTLGRMVMVVWLFLLLVITSSYTASLTSIL
TVQQLSSPITGIDSLIASNSLIGYQVGSFAYSYLKDILNIAPSRLKSLRSPEEFEAALRQGLGNGGVMAI
VDELPYMELFLQNRTDFGIIGRPFTKSGWGFAFKKDSPLANDMSTAILKLAESGKLQEIHEKWFCQLGCP
TDRRKDSVPDQLHLSSFWALYLLSGAVTVLALLIFLLKSIRQYIRYKRNHTDLSSPSNTRCSHVIYSFFD
FIDEKEEAIKRIFAQQDNAQPQTNGS

>SlGLR3.2
MEAYLRKRVFLLLVSWIWVPLAVLGGTGNNTTNATAPLSSFSRPKVVNVGALFTANSVIGRSAEPALVAA
INDVNSDYSILRGTKLNLIFQDTNCSGFVGTVDALQLMEKEVIAAIGPQSSGIAHVISHVMNELQVPLLS
FATDPTLSSLQYSYFLRTVPNDHFQMHAIADVVDYFGWKEVIAIFVDDDNGRNGISVLGDALAKKRAKLT

Downloaded from www.annualreviews.org. Guest (guest) IP: 129.2.89.114 On: Mon, 07 Oct 2024 14:58:42

YKAAFSPEANSSEIDDLLVSVNLMEARVFVVHVNPDTGLSIFSKAKNLGMMVGGYVWITTDWLPSFLDSS
DSVNPETMDLIQGVVALRHHTADSDQKKKFASRWKNFKNVETSSFNSYALYAYDTIWLLARALDLYFKNG
GKITFSDDPRLRDTNGSALHLSSMQVFDQGQKLFQTLIGMNFTGLSGQIQFDSEKNLGRPAYDVLNIGGT
GSRTVGYWSNYSSLSVVPPEILYSKPPNTSTSTQHLYNVIWPGEMVTQPRGWVFPHNGKPLRIVVPYRVT
FKEFVHKDKGPSGVKGYCIDVFEAAIDLLPYAVPHVYILYGDGQRNPSFKNLVNDVVANKYDAAVGDVTI
TTNRTRIVDFTQPYMESGLVVVAPIKELKSSAWAFLQPFTLQMWCVTGVFFLFVGTVVWILEHRHNPEFR
GSPRQQLVTVFWLVIGENTMSTLGRLVLIFWLFVVLIINSSYTASLTSILTVRQLSSGIQGIDSLIASSD
PIGVQDGSFAYSYLIEELGVLESRLRILKTEDEYTSALEKGPQGGGVAGIVDELPYVELFLSNSNCVFRT
VGQEFTKGGWGFAFQRDSPLAVDLSTAILQLSENGELQRIHDKWLSKKVCSSQSNQADDSQLSLKSFWGL
FLICAVACFLALVAFFYRVYCQFRRYDPEPEDQEISEPESVRPSRRTLRSVSFRDLMTFVDRRESEIKDI
LKRKSIDSKKHQGQSSDAQPSSPV

>SlGLR3.3
MAKRMMNVVWIIVSCIVCFGVCSDGLSRNGTSRPAVVSVGAIFTFDSTIGRAAKIAIQEAVKDVNSNSSI
LQGTKLVVQLQNSNCSGFLGMVGALKFMETDVVAVIGPQSSVVAHTISHVANELQVPFLSFAATDPTLSS
LQFPYFLRTTQSDLYQMTAIAEIIEFYAWKEVIAIFIDDDYGRNGVSALDDALATRRCRISYKVGISPGA
TVTRGDVMDVMVKVALMESRVIVLHAYRKLGLMVLSVAHYLGMMGDGYVWISTDWLTTVLDSSPPLPQDT
MDTMQGVLVLRQHTPESKNKRAFSSRWNKLTGGLLGLNSYALHAYDTVWLVAHAIDSFFNQGGTISFSND
TKLQTVEGSNLHLEAMSIFDGGPLLLKNLLESDFVGLTGPFKFSPDKSLIRPAYDIINVIGTGFRRVGYW
SNYSGLSILPPETYYSRPPNRSSTNQKLYSVVWPGNNVQKPRGWVFPNNGKQLKIGVPIRVSYREFVSQI
PGTNNFKGFCIDVFTAAVNLLPYAVPHKFVPYGNGHENPSYTDMVRLITIGKFDGVVGDIAIVTNRTRVV
DFTQPYAASGLVVVAPFEKLNSGGWAFLRPFSAQMWGVITIFFLFVGMVVWILEHRINDEFRGPPKQQLI
TILWFSLSTLFFAHRENTVSTLGRMVLIIWLFVVLIINSSYTASLTSILTVQQLYSPIKGIESLKETDEP
IGYQVGSFAERYLEEIGIPKSRLVPLGSPEEYATALQRGPANGGVAAVVDERPYVELFLSNQCKFRIVGQ
EFTKSGWGFVSFSIPTVFFFFPHSVCPPAFSISLILIL

>SlGLR3.4
MEKEVVVAVGPQSSGIAHVISHVVNELRVPLLSFATDPTLSSLQYQYFLRTVTSDYFQMYAIADLVDYYG
WKEVIAIFVDDDNGRNGISVLGDALAKKRAKISYKAAFSPGATMSDIDDLLVSVNLMEARAKKLGMMSSG
YVWIATDWLPSVLDSSDFNKDTMDVLQGVVALRHHTPDSDKKKTFTFRWKNLKSIKTSRFNSYALYAYDT
VWLVARALDLFFKNGGNVTFSNDPSLRDTNGSSLKLSSLRVFDQGQKLLQILVGMNFTGLTGQIQFDPQK
NLIHPAYDVLNVVGTGLRTIGYWSNYSGLSVTTPEVLYTKPANTSTSNQKLYNAIWPGETIRRPRGWVFP
NNGKPLRIAVPFRVTFEEFVKKDKGPAGVKGYCIDVFEAAIDLLAYPVPHVYILYGDGKRNPSFNSIVND
VAQNKYDAAVGDIAITTNRTRIVDFTQPYMESGLVVVAPVKETKSSPWAFLKPFTIQMWGVTGVFFLFVG
CVVWILEHRMNPEFRGPPRKQLITVFWLVLLTATTSWEARIRENTLSTLGRCVLIFWLFVVLIINSSYTA
SLTSILTVQKLSSGVAGIDSLISSPDPIGVQDGSFAYNYLIDELHVPKSRLRIIKSEAEYVSALQNGPKG
GGVAAIVDELPYVELFLSNSKCIFRTVGQEFTKSGWGFAIWSTKRLILTVFGQAFQRDSPLAIDLSTAIL
QLSENGELQRIHDKWLSNNECSSQNNQVDDTRLSLSSFWGLYVICGGACAVALVVFICRVYCQFLRYDPE
TEEPEISEPESARSSRRSLRSRSFKDLMGFVDKREAEIKEMLKRKNSDNKKQISHISDVQQNSPV

>SlGLR3.5
MKYGSFQTLVSRLHIFVPKFTMRLFWTIILVVLYNGCSSEGVNSTLSARPKVVNIGCMVSFNTLVGKVTK
VAAEAAVEDINSNPDVLGGTKLNMITLDSNASGFLGIVEAIRFMETDTMAIVGPQSSVIAHVVSNIANEL
QVPLLSFAATDPSLSSLQYPFFVRTSPSDKYQMEAIAEMVEYYEWREVIAIYIDDDFGRNGIAALADQLA
KRRCSISYKAAMRPGATLDDARDALVQVALRESRIMVVHTYPTKGLEIFSMARYLGMIDKGYVWIATNWL
STILDAGSPLPSDEKENLEGAITLRIHTPGSELKQKFVSRWSNLTRKAGLAGSSRMSTYALYAYDTVWLL
ARAINEFFNQGGKVSFSKDPRLTELNSGSMNLDSMSIFNGGKLLRDNIFKVNMTGVTGPFSFTSEKELFR
PTFEVINVVGTGFRKVGYWSEYSGLSIVPPETLYSKPPNRSSSNQQLQSIIWPGQITEKPRGWVFPNNGR
QLKIGVPNRASFREFVGKVPGVDSFRGYCIEVFTTAIDLLPYALPYKLVAFGDGHNNPDDTELIRLITAG
VYDAAIGDIAITTNRTKMVDFTQPYIESGLVVVAPVKEQNSNAWAFLSPFTPKMWCVTGVFFLIVGTVIW
ILEHRLNDEFRGPPSKQIVTVLWFSFSTLFTAQRENTVSTFGRIVLLIWLFVVLIINSSYTASLTSILTV
QKLSSPITGIESLVNTKEPIGYQWGSFARNYLIQELRIDESRLVPLNLPEDYAKALKDGPSRGGVAAVVD
ERAYMELFLSSRCQFSILGQEFTKNGWGFAFPRDSPLAVDMSTAILKLSENGELQRIHDKWLSGIACTSQ
STKLEVDRLQLKSFSGLFFLCGLACFLALLIYFVMLACQYCQYYPNSEVASESSRSGRLQTFLSFADEKE
ESVRSRSKRRQLEVTSVRSIDQDASVNGSRTDRSEIYSNRVVSFGESV

Downloaded from www.annualreviews.org. Guest (guest) IP: 129.2.89.114 On: Mon, 07 Oct 2024 14:58:42

>OsGLR3.5
MAGYVTIILLLLLPAAVSGAASGGGGCIGAMVDDTSRAGKEEKLAMEMAMEDFTVSGVDV
GSPAVAVVLCTMASNGDPVRAASAALSLINERGARALVGLHSWQEAAFVAEIGRQAMVPV
LSFAAAAAPSTSRRWPFLVRVARGQHAQMRAVAAVVGSWQWRRVAVLYEDADYGGGAGVF
PHLADALRAVGSEVDRRVPVPASPSGDALRRSLGDLMGGQCRVFVVHTSAKVAVALFAEA
SRMGMMATGYVWIVTDAIAGAIDSLDAAAVSTMQGVIGVRNHISMDTNSKNTRDRLIARL
RKRFRSQYPGDDDDGGGGGGGDNDKTRGPHYPALLAYDTIVAVASAMRKTNATAPTRATD
PNPSSPDSGETIKIAVSSNGTELLREVKSVRFRGVSGEFGFVDGEFSPPVRFQLINVAAP
RYHELGFWSPEHGFSKSAGGCSHRGGDGGGECEPSMRFLGPVIWPGKPWDVPRGWAPPAN
GSPFTVAVPEKAAFPDFVKVTRHHGRGGDDDDEPSFEGFSIDVFKAAVEHLPYNFHYKFV
SFNGTYDSLMQHDYMKSYDILVGDTSISSGRYKFVEFSQPYTESGLVMVVPFSADTWDRS
WIFLRPFSPAMWLLIAAVRLYNGVAIWLMERRHNGDYRGGVWKQVTIVLWLSLAALLSPG
EKERRLRSSLSKASMAVWLLVAVVLATNYTASLSSLMTAQRLGREAAVTAESLRSAAGAV
VGCTEGSVVGRYLEEVLMFPGHRVRRLAGDEEHRRALVSGEVKAAFLRVSHAKLLLAKYC
NELMTTGPVYHVAGLGFVFPKGSPLLADISQAILEVFENGTIQRLETAMLSAYNCTAAAA
AAAMDGGAGDLYRLGPENYWGLFLMTLFASTASLAAYGVFFHHDTSCGSGGGGGGAVITG
GHRKQGDGRKDSATVDPGGSSHGDEAPSSASVTAGHGGKDTEMVVISMA

>XP_015643318.1
MERWPLPVAASAAAVARLSLLVVLCGAISRAAAAAPVRVGVVLDLASGGEGRRSLACISMALDDYYGAND
YSTAAAARARVELRVRDSRGDVLAAAHAVEELMNKNAQVEAIIGPQTSAEVELFAGIAIRNHIPILSFSP
TTSPALSSPPTRFFVRTAASIASQAAPIAAILDVFSWRAAVLLHEDSLYGIGILPALVHAFQVQGQLLAG
SYGARGVVDSVSVPADATDGRLDAALRAVKIMPWRVYVVHMLPALVARLFRRASVAGMMSEGYAWIATAG
VGAAADGLSPDDIEHMQGVVSLRPYVQPTGQVRSFTRRLKARFRRDNPGIDDEDDDDDVAHTSASLLWLY
DTAWAAAAAAADRCLHQSSNAREEHNTTTFLDALLATTFQGLAGRFRLVDGERQVSAYEVVNIIGSGARTV
GFWTPELGVSQDMARRRPKSGSNEELKQILWPGETAAVPIGWSESANGRPLRVAVPVKVGFNQFVAIRRQ
QNQTSAGGAMITGFCIDVFQAVMAKLAYPVAYQYVPVTDNMLSYDKMVNLVHEKKADVVVADMTITAERM
KLVSFTMPFTDSGVSMVVAEKEKANNMWIFLRPLTPGLWITSMAFFFFTGFVVWAIEHRINPRFHGTPCN
QFGVVFYFAFSTLVFSHKEKLESNLSKLVVIVWVFTVLIITTSYTANLTSMLTVGQLQPTINELKKGDYV
GYQQGSFVQNILKDMGFNEDRLRAYATIDQYAEALNMGSDNGGVSAIIDEVPYLKLFVSQYCQGYAIVGP
TYKSGGFGFVFPVGSPLVPDVSRAIVQLAEENRLARIENKWFGEPGSCARKSNSTGDDKLRLKPRSFGGL
FLINAAVSSAALLAHLVAVSSLPTELRRRFAVVVAFGVSGRRGGGAPVAMAGGGAEERISEPHQLVGEPP
NQNHSAAGTSSTVVVQ

>XP_015641432.1
MGCAAKYLTKNDMAFVISREPQRLNKEVNFAAFLDNPNIDDVHNQSILLWSYNVIPATATSTEICRLFSS
VVQTIERELGQGGVLVARRTHADSADTISFYQLAEKLKLSSYEIVTITGEGARGVCSRTSYSGLPQNRST
SNYSFDISSNGNGAVTEDSQSASVGHNRVGLAVTHGTKTPLNPKTQRRNAIESKDKCSKSSCGSGSEKSN
ETLRIAVTRKYGFQNFLNITDLPNGKINATGFSIEVFENAMKKLDHPPCYMFCLFEGSYDDLVGSVSSGK
FNATVGDVSITAERERHVDFTMPYTQSGLSILVLAEKYSKPRIQWIFIKPLTWQLWLAAVSSFLYIAFVV
WMIERPRNQEYQGSSSRQISTSLYFAFSTMTFSHGQIIRSPMSKIVVVIWCFAVVILVQSYTASLSSMLT
TSRLRPSVVDLDQLRHNNDYVGYQNKSFVYSLLNQTFKEDRLKPYANGKEYAEALRRGKVSAIVDEIPYI
RSFMSDQNNSNEFWVFPQTYNILGFAFGFPIGSPLVHNLSVAILDMTRITNKTDSQLTDDHGSHSTPLTL
ENFSGLFVIVGSVSTLMLLISIVRLVVSRCSETANTNAPSIDDDNGDEESNPQQNDTEEPLLEARDNDSR
SADQNGSFAADQEPSQMQSGTSNGHVPAQAQHIQIEMSPA

>XP_015642226.1
MGIRQRRRLLAVAAAFLSLLALRNAAAAASPAPVRVGVVVDMTSGEGRRSLAGISMAVEDFHRRRHRPGS
AAVVELRVRDSRGDDGAAAARAAEDLIKNAQVQAIIVTTEADTAVVARLRRHHRVPILTFPISGGAPPPS
SHHPPHHATATAPPFGADHTSARAALTGILTAIFSSARRAAGSPPHGRRYNAGAPTGRRLDRRRLAGRRS
SSGEVLRIAVPRKTGFQAFVDVRIDPDTKRQNITGYCIDVFNAAMARVRPRRKYEFHVFDGSYDDLVRNV
SSGKFSAAVGDVTITADRENLVEFTMPYTSSGVSLLVPEENDSKPIQWIFVKPLTRDLWLATIGFFFYTG
FVVWMIEQPRNPEYQGSSVRQLSTASYFAFSTLTFSHGQIIKSPLSKIVVVIWCFVVLILVQSYTASLSS
MLTAKRLRPSVKSLDQLLLTGDYVGYQNGSFVGSLLKKRGFMPSRLRSYGTQKEYAEALRKGSMNGGVSA
IVDEIPYLTSFLSNPQYQKEFQMVNRFYKTPGFGFVFPLGSPLVHDLSTAILNLTGETEGSKIEEKWFGS
SEQSTGGDANPSSSSSSDSNPLTLQSFSGLFIISGCISALMLLISVANRVICAKCAKEARVHDVEHGGST

Downloaded from www.annualreviews.org. Guest (guest) IP: 129.2.89.114 On: Mon, 07 Oct 2024 14:58:42

SSSATEQSRPLQIVIDSNPEPDQAVQEDGNDGFQGAQPMQGSVGDERPNPVQNCRHNGTVPEHDAQMEMN
TG

>XP_015642228.1
MEEHRRLAMAAAFVILLTVWSSPAMAMAAAAATEVHSLGLNCNETKGVFCSSNGSKCPSTLSPKFLIPWS
TLISAFSAARLASCKRAFGGGGAQRAHGYGLGRPSRRDDGGWRAAEGGTTTRHTCLPLPPPSQTKRINAG
VRRNLGCLPEVYHKKLKIAVPLKHGFRAFVNVTDQGVTGYCIDLFEAAVNKLPYRLIYEFVVFDRSYDEL
VQSVSSGINDAAVGDITIIADRASHVEFTMPYTESGVSMLVLAKNESESKIEWVFLKPLTKELWFATVIF
FLFTALVIWIIEHPRNMEYQGSNTRQLSTALYFAFSTLTFSHGQIIKSPLSKIVVVIWCFVVLVLVQSYT
ASFSSILTVKRFKPSVTYLDQLLNNGDYVGYQEGSFVNSFLTRRGFSERRLRSYTKKQEYAEALRKGSKN
GGVSAIVDEIPYLTAIVSDPHYQKEFQMLKRIYKTPGFGFVFPPGFPLVHNLSTAMLDVTSGDEGSRMET
KWFGAEAVSPSNAIPNTDSAPLTLRSFSGLFIITGCISTLMLMIRFSMSILANYTQIRDSDVQSPDVGGR
NDAHEESNQAQNSMGCIVVDIHLHEVRIGSSQDIHGSVERAS

>XP_015642913.1
MEARRLAMAAAAAVVPVLLVVWSSAVAAAAAATTGGDVSVALEAYRLDPNGSGSRTGAITAAHGDETKRT
NAGVRRDLGALPRGYGKELKIAVPWKPGFKAFLNVTDRSVGGYCIDVFEAAVKKLPHHLSYKFVVFNGSY
DELVQRVSSGNYDAAVGDVTITAERTIHADFTMPYTESGVSMLVLMENDSKSTIEWVFLKPLTRELWVAT
VIFFLFTGIVIWMIERPRNLEYQGSSSRQFSTALYFSFSTLTFSHGHIIKSPLSKIVVVIWCFVVLVLVQ
SYTASLSSILTAKKLRPSETDLEQILFDGDYVGYQRGSFVESFLIKQGFSKRRLRPYTKKQEYAEALRKG
SMNGGVSAIVDEIPYLTSFLSDRRYEKEFQMLSRIYKTPGFGFAFPPGFPLVHNLSTAILDVTGGDEGSR
IEAKWFGTTAAPPSYAIPNTDSTPLTLQSFSGLFIITGCISALMLMISISKSVLANYTRIRDSEVRSPDA
DGGNGGREERNSAQNVMGDGYVDDRPHHEIRIDSSQDIHGSVERADGEEPGPIQNGSVPANSSQTG

>OsGLR3.1
MKFIFYLFSIFCCLCSCAQSQNISGRPDAVRIGAQFARNSTIGRVAAVAVLAAVNDINNDSNILPGTKLDLHMH
DSSCNRFLGIVQALQFMEKDTVAIIGPLSSTTAHVLSHLANELHVPLMSFSATDPTLSSLEYPFFVRTTVSDQF
QMTAVADLVEYYGWKQVTTIFVDNDYGRNAISSLGDELSKRRSKILYKAPFRPGASNNEIADVLIKVAMMESRV
IILHANPDSGLVVFQQALKLGMVSNGYAWIATDWLTSYLDPSVHLDIGLLSTMQGVLTLRHHTENTRRKSMLSS
KWSELLKEDSGHSRFLLSTYGLYAYDTVWMLAHALDAFFNSGGNISFSPDPKLNEISGRGLNLEALSVFDGGQL
LLEKIHQVDFLGATGPVKFDSGGNLIQPAYDIVSIIGSGLRTVGYWSNYSGLSVISPETLYKKPANRTRETQKL
HDVIWPGETINKPRGWVFPNNGNEIKIGVPDRVSYRQFVSVDSETGMVRGLCIDVFVAAINLLAYPVPYRFVPF
GNNRENPSYSELINKIITDDFDAVVGDVTIITNRTKVVDFTQPYVSSGLVVLTSVKRQNSGGWAFLQPFTIKMW
TVTGLFFLIIGTVVWMLEHRINDEFRGPPAKQLITVFWFSFSTLFFAHREDTRSTLGRFVIIIWLFVVLIIQSS
YTASLTSILTVQQLTSPITGIDSLITSDVPIGFQVGSFAENYLAQELGVAHSRLKALGSPEEYKKALDLGPSKG
GVAAIVDERPYIELFLYQNPKFAVVGSEFTKSGWGFAFPRDSPLSVDLSTAILELSENGDLQRIHDKWLASDMS
SMSQASELDQDPDRLDVYSFSALFLICGLACIFALAIHACNLFYQYSRHAAEEDPAALQPSASDGSRSLSRRSK
LQSFLSFADRREADIRRAAKEKASGLGGSGGSMSGVSFTSSGSGSTTASC

>OsGLR3.2
MKIGLLMLLVLFLVMSPDGIRRSLAARPSIVNIGAILRFNSTIGGVSMIAIQAALEDINSDSTILNGTTLKVDM
RDTNCDDGFLGMVEALQFMETDVIAIIGPQCSTIAHIVSYVANELRVPLMSFASDATLSSIQFPFFVRTAPSDL
YQMDAVAAIVDYYRWKIVTAIYIDDDYGRNGIATLDDALTQRRCKISYKIAFPANARKSDLINLLVSVSYMESR
VIILHTGAGPGLKIFSLANQLSMMGNGYVWIATDWLSAYLDANSSVPDETMYGMQGVLTLRPHIPESKMKSNLI
SKWSRLSKKYSYSYLRTSSYAFYVYDSVWAVARALDAFFDDGGKISFSNDSRLRDETGGTLHLEAMSIFDMGNN
LLEKIRKANFTGVSGQVQFDATGDLIHPAYDVINIIGNGMRTVGYWSNYSSLLSTVLPEVLYSEPPNNSLANQH
LYDVIWPGQTAQTPRGWVFPSNAKELKIGVPNRFSFREFVTKDNVTGSMKGYCIDVFTQALALLPYPVTYKFIP
FGGGNENPHYDKLVQMVEDNEFDAAIGDIAITMSRTVTTDFTQPFIESGLVILAPVKKHIVNSWAFLQPFTLQM
WCVTGLFFLVVGAVVWVLEHRINDEFRGSPREQIITIFWFSFSTLFFAHRENTMSTLGRGVLIIWLFVVLIIQS
SYTASLTSILTVQQLDTSIRGIDDLKNSDGPIGFQVGSFAEEYMVRELNISRSRLRALGSPEEYAEALKHGPKR
GGVMAIVDERPYVELFLSTYCKIAVAGSDFTSRGWGFAFPRDSPLQIDLSTAILSLSENGELQRIHDKWLKTSE
CSADNTEFVDSDQLRLESFWGLFLICGIACVIALLIYFFTTVRKFLRHEPPEDPTPRPGGSTTLPDERTPPKNG
QEKCNCRNFISFLDHKEPPKKKRSLSLTPTTPLSNFTALEIEGPVRTVRNGSVVDI

Downloaded from www.annualreviews.org. Guest (guest) IP: 129.2.89.114 On: Mon, 07 Oct 2024 14:58:42

>OsGLR3.3
MEKNVVAVIGPQSSGIGHVISHVVNELHVPLLSFAATDPTLSASEYPYFLRSTMSDYFQM
HAVASIVDYYQWKEVTAIFVDDDYGRGAVAALSDALALSRARISYKAAVPPNSNAATIND
VLFRANMMESRVFVVHVNPDAGMRIFSIANKLRMMDSGYVWIVTDWLAAVMDSSMSGDLK
TMSYMQGLIVLRQHFPDSETKREFISKWNNVARNRSIASGLNSYGFYAYDSVWIVARAID
QLLDNGEEINFSADPRLHDSMNSTLRLSALKLFDSGEQLLQQLLLTNFTGLTGQLQFDSD
RNLVRPAYDILNIGGSVPHLIGYWSNYSGLSVAAPEILYEKQPNTSTSAQRLKNVVWPGH
SASKPKGWVFPNNGQPLRVGVPNKPSFKELMSRDTGPDNVTGYCIEIFNAAIKLLPYPVP
CQFIVIGDGLKNPNYDDIINMVAANSLDAAVGDFAIVRNRTKIAEFSQPYIESGLVIVVP
VKEASSSAWAFLKPFTLEMWCVTGVLFIFVGIVVWILEHRTNEEFRGSPRRQMITIFWFS
FSTMFFAHRQNTVSALGRFVLIIWLFVVLIINSSYTASLTSILTVQQLATGITGLDSLLS
SALPIGYQAGKFTRNYLIEELNVPESHLVPLNTIDEYADALNRGPKDGGVAAIVDEMPYI
EIFLSYHCNFRIVGQEFTKEGWGFAFQRDSPLAADMSTAILQLSESGQLQRIHDEWFSRS
SCSSDDSEMGATRLGLRSFWGLFLMCALICVFALVMFFARVCWQYSKYSGSEEPDEPKDD
SAGTAEIAAEAVAEMQRRRPKRLGSFKELMQFVDKKEEEVRKSMKRRPSEKDNQGVGSSD
AQSVA

>OsGLR3.4
MAGDQLLRRLLFLLWVVALAVPGLAARPANVSIGALFTFDSVIGRAAKVAIELAVADVNR
DDGVLNGTYLSVVEQDTKCSGFIGIIQGLQVMEKKVVAVVGPQSSGIGHVVSHVADELRI
PLVSFAATDPTLGSSQYPYFLRATHSDFFQMAAVADIISHYAWREATLIYVDNDYGRAAL
DALGDHLQSMRSKVSYRAPLPPAADRAAITDLLLRVSMMESRVIVVHANPDSGLDIFAAA
QSLGMMSSGYVWIATEWLAALLDSDSSPPRKTTALALLQGVVTLRQYTPDSDAKRSLMSR
FAARLQAHNTTGGINAYVLFAYDAVWMAARAVDQLLVDGSNVSFSDDARLRAENETGSAL
RLGALKVFDQGEQLLSKMKTLNFTGVTGQVRFGDDRNLADPAYEVLNVGGTGVRRVGYWS
NRTRLSVTAPEQEQNGKKKKQQGEELYSVIWPGETASTPRGWVFPNNGKALRIGVPYRTT
YKQFVSKDAGGPDGASGYCIDVFKAAVALLAYPVPVSYVVVGDGVKNPSYGELVQRVAEG
ELDAAVGDISIVTNRTRVVDFTQPYVESGLVIVTAVRERASSAWAFLKPFTREMWAVTGG
FFLFVGAVVWVLEHRSNTDFRGSPRKQLVTVFWFSFSTMFFAHRENTVSTLGRLVLIIWL
FVVLIINSSYTASLTSILTVQQLSTGIQGLDGLIASSDPIGFQVGSFAKSYLMQELGVPE
SRLRELAITDYASSLQTGVVAAIVDELPYVELFLSTNCQFRTVGQEFTKSGWGFAFQRDS
PLAVDLSTAILTLSENGDLQRIHDKWLSPGQCASQGTDVGADRLNLSSFWGLFLICGVAC
FIALLIFFFRTLRQYFRYHGHADIDDDDDSENKATPFPVDGGERMSSRRPARLASIRDLM
TFVDMKEAEVKRRKKMMNEDSSSCGRRLDMDSHSHRSMPTSANANAAPPSSSFSSV

>XP_015612655.1
MAACARTAPLLLLLLAFSFAFAGSVTERKAEEFHVGVVLDLGTTVAKVARTSMSLAVEDFNAVHPSYTTRL
VLHVRDSMGDDVQAASAVLDLLENHKVQTIVGPQKSSQATFVSALGNKCQIPIISFTATSPTLSSRTLPY
FVRATLNDSAQVNSIVSMIKAYGWREVVPIYVDNDYGRGIIPSLVDALQQIDVHVPYQSEIDQSSTSEEI
TQELYKLMTMQTRVYVVHMSPSLGSVLFTKAKEIGMMSEGTVWIITDGLTNLIDSLNPSVVEAMNGALGV
KVYVPISTELDSFTKRWYMRSRIDHPNDPTMKLNIFGLWAYDSIWAIAQAAEMSKVRKAMFQRPSSEKNL
TNLETLQTSINGPALRKAMLQNKFRGLSGYFDLSDGQLQVSTFRIINVAGKGYREIGFWTARNGISKALE
QKRSHPTYESTKPDLNIVIWPGEVTELPRGWELAVRGKKLQVGVVKGHYPEYIDADEDPITGVTTARGLA
IDVFEEAVKRLPYALAYEYKLFNITGIASSSYDEFVYQVYLKKYDIAVGDIAIRYNRSLYVDFTLPYTES
GVAMVVPVRESINKNAWIFLKPLTPGMWFGTIMVVIYTGVVIWLLELLGNNKNVDATISRQSMTSIYFSM
FEDKEKVKRLISRIVLVVWLFFILVLKSSYTASLTSMLTVQQLQPTAHDVHELLKNGEYIGCGSGSFVMG
LLEELGFPRSMIKPYHNPEDIHNALSRGSKNGGIAALVGEIPYIKLFLAKNCKRYTMIGPIYKTAGFGYA
FPKGSPLVGDISQAILNITGGDTIIQIEQKWVRDKNSCQNEGSIIGSGSLTFASFEGPIILTGVVSTSSL
LVALIMYFYRNKKIKPHHSDSEQISSHGENER

>XP_015612148.1

Downloaded from www.annualreviews.org. Guest (guest) IP: 129.2.89.114 On: Mon, 07 Oct 2024 14:58:42

MEKAPPGAILLLMLFAHSCAVALNATNDPGADEFHVGVILDLGSLVGKEARTSISMAVEDFYASHKNYRT
RLVLHVRDSRGNNFQAASAALDLLNNYNVKAIIGPQKSSEAFFMTDIANISEVPVISFTTTSPSLTSDNN
PYFLRATINDSTQVNSIASLIKYYGWREVVPIYIDTDYGRSIIPDLLEALQGNDARVPYQSIIPQSATSE
QITQELYKLMTMQTRVFIVHMTSPMASVLFTKAKEVGMMDKGYVWIVTFGVASLIGSLNPSVLEAMNGAL
GVGVYVPKSTELDNFTVRWNTRFRMDNPNDPLLKLSIFGLWGYDTIWAVAQAVEKAKSTKDTVQIQHMTN
SMTSLKVPKETENGLKFLNAILQYKFRGLSGYFDLSGRQLQPSTFQIINIVGKGWRDVGFWTAQDGFSQR
LTRPRSNGTYLSTKPDLNPVIWPGESTNIPRGWEIPTSGKKLQVGVCTSDGYPEYIYAEKDPLIVGMTKA
SGLAIEVFEETVKRLPYALPYEYVFYNTTENISSSYDDFVYQVYLKKYDIAVADITITYKRSSYADFSLP
YTESGVAMVVPVRKRINTTTWIFLKPLTFGMWSASIILFIYTGVVVWLLEFLGNNKAVRGPIPKQMVMIY
FSLFVKKEMVERLLSRIVLIIWLFFLLVLTSSYTASLTSMLTVQQLQPTVTDVHELLKNGEYVGYQGGSY
VKDLLDELGFDKSKIRQYDSTDGFRDALSRGSSNGGISAVVDEIPYIKLFLAKHCEGYTMVGPIYKTAGF
GFAFQKESPLRGDISKAILNITGGDTIIQIENKWIGDQNKCRNVGPVTISGSLTFESFKGLFILTGIAST
SSLLIALVIYFYKNKQVQSGIGDAEQDFPQEFKADTIEEEKEQEETGAKGKQNMNLQNSTVKRSASIVIH
RGERATGARVVPISGSARF

>OsGLR2.4
MERAPERAAIFFLLLSLTVAQYTTESGTVHRSFKTKLVLHIRDSNGDDIQAASEAIDLLENYNVKAIVGPQKSS
EATFVSDLGNKSQVPVISFTATNPTLSSIDVPYFLRGTLSDVAQVNTIAALIKAYGWREVVPIYEDTDYGRGII
PYLADALQEFGAYMPYRSAISKSANTDQVEQELYKLMTMQTRIYVVHMSVNIASILFTKAKELGMMSKGYAWIL
TDGISNIVNSLSPSILEEINGAIGVRFYLPASKELHDFTARWNKRFKQDYPNDPPSQLSIFGLWGYDTTWALAQ
AAEKVNMADAIFQKQKDTKNTTSLGTLGISTIGPKLLDSILHSKFRGLSGEFDLRNRQREFSTFQIINVVGSRS
KEIGFWTAKQGIFRQLNENKTTNINFVPDLDPVMWPGEVYTVPKGWQIPTNGKKLRVGVRTSGYPELMKVEKNP
VTNEVTASGYAIDVFEEVLRRLPYAIPYEYVAFDNGQGVNSGSYNDFVYQVHLGVYDTAIGDITIRYNRTSYVD
FTLPYTESGVAMIVPVKDDRDKNTWVFLKPLTTDLWFGSIAFFIYTAIVIWLLERRINNAELTGSFFRQLGIAI
YFSFFADRERVDSILSRLVVIVWVFVLLVITSSYTANLSSMLTVQQLQPTVTDVHELLKNGEYVGYHNGSYVGD
LLKGLGFDRTKIRAYDNSDDFADALTKGSQNGGIAAVVHEVPYIKIFLAKHCKGYTMVGPIYKSEGFGFAFPKR
SPLVYDFSREILSILEGDSIIHIEKKWIGDQHACQNDGTVIGSSSLNFNSFSGLFLVTGVASTSALLIALLMFL
YKNKHRIRNSISRDQTRSRYGPEHINEQNEERVIDSSQVQNLQLTVPDDSEYTCQQEEEISIELSPASGFQPSP
DFSSQEHRAVASSTIVANSSSQVPRI

>OsGLR2.3
MGRAVGRAAFLFLFLSLTVAQNITKNGAGTLDVGVILHLKSLVGKIARTSVLMAVEDFYSVHRNFKTKLVLHIR
DSNGDDVQAASEAIDLLENYNVRAIVGPQKSSEVTFVSNLGNKSQVPVISFTATNPALSSINVPYFLRGTLSDV
AQVNTIAALIKAYDWREVVPIYEDTDYGRGIIPYLADALQEFGAFMPYRSAISESATTDQLERELYKLMTMQTR
VYVVHMSLNIASILFAKAKDLGMMSEDYAWILTDGISNIVNSLNTSILEKMNGAIGVRFYVPASKELDDFTTRW
NKRFKEDNPNDPPSQLSTFGLWGYDTIWALAQAAEKVRMADAIFRKQKDGKNSTSLGTLGISTIGPELLDSILH
SKFQGLSGEFDLGNRQLEFSTFQIINVVGGRSKEIGFWITKHGIFRQINENISKTTNVNSMPGLNRVMWPGEVY
TVPKGWQIPTNGKKLRVGVRTSGYPEFMKVERNTATNEITASGYAIDVFEEALKRLPYAIPYEYVAFDDGQGVN
SGSYNDFVYQVHLGVYDAAIGDITIRYNRTSYVDFTLPYTESGVAMIVPVKDDRDKNTWVFLKPLTTGLWFGSI
AFFIYTAVVIWLLERRINNAELTGSFFRQLGIAIYFSFFADRERVDSILSRLVVIVWVFVLLVITSSYTANLSS
MLTVQQLQPTVTDIHELLKSGEYVGYRNGSYLSD
LLEGLGFDRTKMRAYENPDEFADALAKGSQNGGIAAVVHEVPYIKIFLAKHCKGYTMVGPIYKSEGFGFPPIVS
NIHCWIMLYAAFPKRSPLVYDFSRAILNITEGDSIIHIEKKWIEDQHACQNDGTMIGSSSLNFNSFSGLFLVTG
VASTSALLIALMMTLYKNKHRIRDSIRRGQTQKEYERETINEQNQERTIDSNQVQNLQLTVPDDSNEYTCQQEG
EISIEISPASGIQTSQDIASHRTSRNG

>OsGLR2.2
MGMERAAGRAAIFFLFLSLTVAQNITGSGEDTLNVGVILHLKSLVGKMARTSILMAVEDF
YKAHRNFKTKLVLHIRDSNGDDIQAASEAIDLLENYNVRAIVGPQKSSEATFVSDLGNKS
QVPVISFTATNPTLSSINVPYFLRGTLSDVAQVNTLAALAKAYGWREVVPIYEDTDYGRG
IIPYLADALQEFGASMPYRSAISESANTDQIERELYKLMTMQTRVYVVHMSTNIGSILFK
KAKDLGMMSEDYAWILTDGISNIANSLSPSILEEMSGAIGVRFYVPASKELDDFTTRWNK
RFKEDNPNDPPSQLSIFGLWGYDTIWALAQAAEKVRMADAIFQKQKDTKNTTCLGTLRIS
TIGPKLLDSILLSKFRGLSGEFDLRNRQLELSTFQIINVVGSQLKEIGFWTAKHGIFRQL

Downloaded from www.annualreviews.org. Guest (guest) IP: 129.2.89.114 On: Mon, 07 Oct 2024 14:58:42

NKNKSKTTNMNSMPDLNPVVWPGEVYTVPKGWQIPTNGKKLRIGVRTNAYPEFMKVESNP
VTNEITASGYAIDVFEEVLKRLPYAIPYEYVSFDNGQGINSGSYNDFVYQVYLGVYDAAI
GDITIRYNRTSYVDFTLPYTESGVAMIVPVRDDRNKNTWVFLKPLTTDLWFGSIAFFVYT
AIVIWLLERRSNNAELTGSFLRQLGIAIYFSFFADRERVDSILSRLVVIVWVFVLLVITS
SYTANLSSMLTVQQLQPTVTDVHELLKNGEYVGYPNGSYVADLLRGLGFDRTKLRAYNDL
DGFADALAKGSQNGGISAVIDEVPYIKIFLAKHCKGYTMIGPIYKSEGFGFAFPKRSPLV
YDFSRAILSITEGDSIINIEKKWIGDQHACQNDGTIISSSSLNFNSFSGLFLVTGVASTS
ALLIALVMFLYKNKHRIRNSIRRDQTQKGYEAERINEQNQEMTIHSNQVHNLQLTVPDDS
DEYSCQQDGEISIEQSPASEIQTSPYFASHAQQ

>OsGLR2.1
MERAPQTILFLLLLVHFTVAQNANKTGVVDGFPVGVILDLQTMVGKIARTSILMALDDFY
AAHTNYSTKIVLHIRDSGSNNVQAASAALDLLENHNVQIIIGPQTSSQASFVSDLGNRSQ
VPVISFTATSPSLYSASLPYFVRATLNDSAQVQSIACLIKTYGWREVVPIYEDTDYGRGI
IPYLVDALQDIDARVPYRSVIPLSVTSEEISQELYKLMTMQTRVFIVHMSSTLAASLFTK
AKEVGMMSKGFVWIMTDGITNIVDSMSTSVVEAMNGALGIQFYVNNSELDSFTIGWNRRF
QIDNPNDPPLKLSIFGLWGYDTIWAVAQAVENVGVNNRTSIQKPSVARNSTSLENMETSV
YGPELLKVILRNKFRGKSGYFDLSNRQLQVSTFRIINVFGKGWKDIGFWNEGNGILRQLN
LGKSTTKYADSVLDLNPVIWPGKSTEIPKGWEIPASGKKLQVGVHKSAYKEYMTNQRDPI
TGATKASGFSIDIFEEAVKRLPFALPYEYVAFDTSRDTSTGSYDDFVHQVYLKKYDVAIG
DITIRHSRMAYVDFTVPYTESGVAMIVPSKGTVDKTWIFLQPLSRDLWVATISMFFYTGC
VVWIELNVVKLTGWKGKMNYMQVGVRLETSFANQLKENERVERILSRIVLIVWVFFFLIL
SSGYTANLATMLTVQQLKPTINSIDELRKSGENIGYHDGSFVKNLLEDLNFNTSKIKAYD
TPDDFYNALSKGSNNGGIAAFVHEVPYIKLFLAKHCKEYTMVGPFYKTAGFGYAFPKGSP
LLGDISKAILSITEGDIIMQLENKWIGYQNDCKSVDSAVGTVSDPDKLNVDSFKGLFILT
GVASTSSLLIAVMIYYYEKKKSMTSMQPDQNGEGLEENHKPQEVNEGDREEENNQPGAST
GQSGQQQQQTGAREMSNINLQTSSVRRNSSIFIWHERNLGARVAPISSSSHF

>OsGLR1.2
MEAGARLAFVMPLVVVLLLMIFSLGVRGVDVVVDGGGGGAAAARRRRVEVGVILDRRTWL
GNISWACMELAVEDFYADEERASYTTALRLHLRDTRLDAVDAASAGVDLLKNVHVQAIVG
PQTSAQAKFLAELGEKSSVPVVSFSANSPCRTASQTPYFIRTAWNDSSQAEAIASLVQRF
NWRDVIPVIEDDDSNTRFIPDLVDALRNAEIRVTHRCKIHPSAGADDIKKVVLSLKEKWT
SVFVVRMSYQLALSFFKHAKDEGMMGQGFVWIAAYGLTDIFDVVGSPAFDVMQGVIGMKP
YVNDTKQLQNFRQRWRKMYKSENPGTTLSEPTLSGLYAYDTVWALALAAEKAGYVNSDFL
LSEKNNGSTDFDRINTSNAAKKLQSTLLNIDFQGMSGKFQFQDMHLLSMTYEIINIVGEE
QRVVGFWTPEFNISRGLNTKADVNEIIWPGGETTVPRGWLFPMNKTLKIGVPAKPGFSGF
IKKEKYNFTGLCIEVFEEVLNGLPYKIPHDYVEFGNGKGESNGTYDELIYKVYQNDFDAA
VGDITILANRSLYVDFTLPYTESGVRMLVPVQDQRQKTAWTFLQPLTADLWLGTAAFFVL
TGFVVWFIEHRTNEDFRGPPVNQIGSVFYFAFSTLVFAHRQKIVNNLSRVLLVIWLFVVL
ILQRSYTASLSSILTVEQLQPTVTNLDEVIRKGANVGYLNDSFMPELLKRLKIDESKLIA
LDSPDEYNEALSTGRVAVVVDEIPYLKVFLSKYCHNYTMVGPTYKFDGFGFAFPLGSPLT
AEISRGILNFTSSNRMAQLERELYNNRTCPDKDDSQTSSSLTLRSFLGLFIITGASSLLA
LFLHVVITLYNHRHDLSSASSSQSSWCGWFAILLKIFHEGDRPNAPQLDEPAVSNANTTA
DTPWSTPDHHIIENVDSGSDVESVREEDREDFVQGPDPPSFAYMHSERGQ

>OsGLR1.1
MAGHTPNPLFLLLFLGYLLFAAAQPPLTVTVGLIIDGGSPVGKIANTTIPMALDDFYAAFPRSPARVRLLHRDS
RGDVVAAASAALELMEGRGVRAILGPQSSVESAFVADLATRAEVPVVSFSATSPSVSPGGGRFFARAALSDAAQ
AGAIAALARLFGWRRVVPVYQDDDYGAAFVPFLVDALTAEGSEVPYRCALPAGADADAVAAAMYRMESLQTRAF

Downloaded from www.annualreviews.org. Guest (guest) IP: 129.2.89.114 On: Mon, 07 Oct 2024 14:58:42

VLHARPDLAGRVLAAAEAAGMMGEGFAWVITDGLTGLLGSINAPQGVIGLAPYVPTTPRLRDVRRRWVRRFMAE
HPAADAEHAEMGSYAVWAYDAAWAVASAAEHLTAGDLSPPQGGLVGGKGGPTDFAGLGKSRSGKKFLEAITSTT
FDGLGGRFQLVDGELAVHAFRVLNIMDRGKERSIGFWTKDGGLTRHLGVGGGGGGELAPVIWPGESTVVPRGWV
VPTSARRLRVAVPGSVNPGYRAIVHLDVDAATNRTTAGGFVVEVFEAAVRLLPYALPVEYVKAESMPYDKLVQM
VADGAFDAAVADMTITAARSSYVDFTLPFMASGIAMVAPLRDVGRGGERTWVFLKPLRYDLWLASAAFLLLTGF
AVWFVEHRGNAEFRGPPWHQLGTLLYFGFSTLVFAHREDLRSNLARLAAVVWFFVVLILQSSYTASLTSMLTVP
RLEPSIAGYAALWRGAERVGIMNNSFMRGAMTRSGFPPARLVPYGAAQSFHEALLNGTIGAVVDETPYLRIFLK
SYCDRFAMAGGGGGGQPNKTGGFGFAFPKGSPYVADLSRAILALTESEEMNLIERKWFGESDGCAAAQAAGGPF
TSDSLSFGSFWGLFLITGATSLLCCAVHLATFVASNRGAIRDIVATSTHPFRRLAELYDGRDLSAHTFKAKDGG
AAASPPVVHDAAGSPISLHMGAWSPQPSSTMAGGEIEPAGGEANEEEATTTTAARDPDGAGENGRGQ

>OsGLR1.3
MTMQTRVFIVHMLPARASRLFARAKALGMMTKGYVWIVTDSIGIVLDVLPQHSIESMEGIVGFRPYIAESTRIT
DFSSRFTTLFRTKYHPNTDIRMAKPTIFQLWAYDVAWAVATATEKVHRTRSLNPTFHPPGNIGKNLVDDLPALP
AGPELLNSILQGEFDGLAGQFRLIDRHLQVPTYEIVNVIGEKTRVIGFYSPDSGLTMSVNSRIIHGDAKFSTSS
SDLENIVWPGDSTTVPKGWDFPVNAKILQIGVPVRRDFKTFVNVETNPNTNRSTVSGYSIDMFEAAVKKLPYAL
RYEYIPYDCAVSYDLLVSQVFYKKFDAAVGDVTIIANRTRYVDFTMPYTESGVSMLVLSKSDDEPTTWIFLQPL
AKDLWIATMIFIFFTGLVVWVIERPINRDFQGSKWKQCITAFYFAFSTLTFSHGQKIQSIQSKIVVVIWCLVLM
ILVQSYTASLSSMLTAERLQPSVTDLKQLLANGDSVGHQSGSFVQSILKKLKFDDHKIKVYSTQEEYAKALRMG
SKHGGVSAIFDEIPYLNSFCSKYGREFQMVGPIDRTSGFGFVLPKGSPLVPDLSEAILSLTEEPERLKIEKTWF
MDSSLDYYGSHSKGSSRISFQSFQGLFIIVGCLLGAVLLINFSKFLYDKCKEMRGFGSDRVHSGERVVCYGEAQ
PQPPQIVMVDRRSCAC

> OsGLR1.4
MARRSCFFFLLPLLVAALAGSPVVTAQRNALPAAAAAAASVRVGVILNLTSAVGVRRRVG
IQMAVEDYYAANPGSATRVELHFRDSAGDVLPAASAAVDLIKNVQVQAMIGPPSSAATEF
VAHIGSHSRVPVLSYSATSPSLSPAQTPFFVRAAVNDSFQAAPVAAVLDAFRWRAAAVVY
EDSPYGSGILPALADALQGAGAKIMDRTAVPVDATDDRLDALLYRLRAMPTRVFVVHMLH
NVAGRLFRRAKMLGMMSDGYIWVATDGVATFMDRFSPEEVDAMQGVVSLRPYVQETDAVK
NFSARFKARLRRDHPTVDDVREPTVLRFWAYDTAWAIAAAAESAGVAGPAFQTPQTSAPL
TDLDRLGVSATGTALLNAVLSTTFDGLAGKFRLVDGQLQPPAYEVVNIIGKGARTVGFWT
PEFGITQDLNAGSAKTLRQILWPGEPRDTPRGWTVSPSGLPLRVSVPTKRGFTQFVDVGN
VTATGRRNITGYCIDVFDEVMKIMPYPVSYVYDPYPDSPESYEKLVDQVSSQKADAVVGD
VTITASRMEEVDFTMPFTESGWSMVVAVQKETSTSMWIFLQPLTTSLWLASLAFFCFTGF
VVWVIEHRINEEFRGTPWQQFGLIFYFSFSTLVFSHKEKLESNLSRFVVIIWVFVVLILT
SSYTASLTSMLTVQKLQPTVTDVRELLRRGDYIGFQEGTFIVPVLEKMGFEGRMRSYSTV
DQYADALSKGSANGGVAAIFDEIPYLKLFLSQYCNGYTMVGPIYKTDGFGFVFPRGSPMV
ADVSRAILTLAEGEKMAQIEKKWFGEPGACQSQGSAVGSSNLSFRSFGGLFLITGVVTSA
MLLIYLAVFFYRERDELRAAEAAAAASGSGSGSGSRSLRRLRAWARHYDQKDLKSPTFKR
RWSDESVRNGSEYAASRTPRWGDESPCNVAGAADADAGRIPEEVVGGMSPFSISTSSEER
NGAVSPAAAEFDNSSDRAAVVAGTSQPR

>*Mp*GLR
MQLWQLLVVVCAWWWWSVLIFTTWTGGQVQGANTTLRIGALFAYNTSIGNEARTAITLAV
QDVNANDTILKDIKLEIRTSDSNCTSFQGAAAAVDMLKTNVVAIVGPQTSEVAQFTADLA
SATQVPLVSFSATDPSLTEGSNHYFVRTVHNDAVQMAAIAAVIELYGWREVTAISTNDYV
GVNSIDALSDSLQLVGATLGFKTLISPDVEKKDMIPVMTELSLLESRVFVVHVQPRIARM
FFSTARSMAMMTTGCVWILSEATTGLLDDLPSTDSIWASLQGVVGVRAYVPSTPELDSFH
ARFKQYMQSSATSSLLSNRAVMNLYGLYAYDAVWMVAHAIENFVNHGGNFSFIPRPPIAD
DAGGTTELASLKILEDGDAFMEQLKSTQFEGLSGPIGLNKFGDLEHTDYEIVNIFGTAQR
VVGYWNNETGFSQNPFVFNSSAPPSPSRPPSSRAAEPPPPKLQDVIWPGGTAQVPKGWVV
PKNGKPLLIGVPYKVGYREFVDINPNRTMFHGFCIEVFQAALTFLPYSVSYKFEVYGNGT
NTPNYDDLVEEIVNKRYDAVVGDVTITTKRSKTVDFTQPYSASGLVVVVPTKSGGSNHAW

Downloaded from www.annualreviews.org. Guest (guest) IP: 129.2.89.114 On: Mon, 07 Oct 2024 14:58:42

AFMRPFTPLMWCTTGLFFLFTGLALWILEHKKNRDFRGRPKKQLVTTLWFIFSTLFFSQR
EKIKSTLGRAVLIIWLFVVLIVTSSYTASLTSILTVQQLFPTIQGIAGLVTSNVAIGYQA
GSFVAEYLQQLNVPKERLVPLGTMSEYADALQKGLVGAIVDELPYVQVFLSSECAFMIAG
QEFTKSGWGFAFPKGSQLAVDMSTAILALAENGDLQKIHDYWLNAHDCTSPGLVVDPNDN
ELGLNTFWGLFLITGTASIFCVVLYYSRLIWQHHKTYRDEEFSESETLSSRASRGRSFLR
SLVSFIEEAEVAATVARADASANNSQRKHRDRRRSRSSFGDERVGSGRSDIPDHHEHPDH
ASDRHNHVMDRNSVI

>SmGLR1
MRWEMWRCWRHFSPCEEIFRAMGAVVFRLALLLVVVLGSLSDLAEWPASVKIGTLLALNSTAGHTGMVAI
QMAVEDINIRNSSFLNGTKLEIITANSNCSAFQGAASAMRLFEQNVVAIAGPQASVVAHFVAHMAAATQV
PLVSASATDPTLSEYQFPFFLRLARSDRMQMEAVAGIISVYGWREVVAIYSDDDYGTNGIDTLGDALVGF
GASIVFKAALDPAIDRTGISKILAGVAQMGTRIFVVHLQPAMGLTLFSEAYLLRMLDKGYVWIATEAIIS
TLDTIYLDSNYVQATQGVIGTRSYVPKSPQLEAFATRWKKIAEEEGSGLIYSQYNAYDLYAYDSIWMIAY
AVRKFLLNRGNFSFVSPTGFQANSGGGSDLAKLKILLEGQALLEDFLETSFEGVSGLVQLDKRGDPSDSA
FQIVNMVGKGLRTVGYWTNATGCSTVEPGTNGSIKSDEQKLEDVIWPGGAVRVPRGWMVPKNGRPLVIGV
PNKQGYKEFVDTALGPDNATVFHGFCIDVFQAALSYLPYTVPYSFQLYGNGTSTPSYDELVQKVVNKEYD
AVVGDITITTKRAKIVDFTQPYTTSGLVVVVPLKKGATNHAWAFMRPFTPAMWFTTGAFFLFTGVVMWLL
EHKKNRDFRGRPKKQVVTTLWFSFSTLFFAQSKSVFVLYISCRILKQQACIFVGEDVKSTLGRAVLIIWL
FVVLIINSSYTASLTSILTVQQLMPTIQNIAGLVASNVPIGYQAGSFVEEYLLQLNVPRDRLVPLDSLSA
YTAALQKGPKSGGVGAIVDELPYVQLFLSSECDFTIAGQQFTKSGWGFAFQKGSQLAIDMSTAILTLAEN
GELQRIHDTWLNGYDCGSQKVQIDSNELGLGTFWGLFLITGTASIICLFVYYTKMLLRYRRILKAQKEEC
SSPDNSIQDNSRRSSSFLRSFVTYVEESEVPKKHRNSSLKKKEGGGTGSSRREEDRSPDNNGGCSSSEAT

>SmGLR2
MAWKLFVFFLLFHVFSVFVVVLAASPPENVTIGALLALRTRIGRAARVAIQLAVKEINEDQTLLNGTRLL
VQISDDNCNAVQGAAAAVELMQRNRVVAIAGPQTSEVAHFVAHMGTVTKIPIVSFSATDPTLSESQYPFF
IRNTHSDRIQMEAIADFVKLFEWKEVVALYSDDNFGTNGIMELHDELSKVGATIPFRAAVSRSMNKDDIG
EILAKFGDAGGRIFVVHTDASVGRAVLTEAYDLRMLTTGFVWIVTETLSSVLDGVYSDDEFVAAAQGIVG
TRSFIPGSPQLERFKSSWRSFNVNRTRGGYRSSNVNLYGLYAYDTIWMIAYAIDGFLAANGSFEYEAMKC
PPGGERRLDLARLSVAKFGARVLREIVKTKFSGISGKVELSAGGELKGSDLEVVNMYGRGLRTVGYWNKG
TGFSVDAPSEDRPQMESVSRLQKKLHHIVWPGDNLHVPRGLMIPKTGRELVIGVPLKQGYKEFVDLTIDV
SNVSTFHGFCIDVFKAALSSLPYTVTYSFVGFGDGNSTPSYDELVEKVANKKFDAAVGDITITRKRAKLV
DFTQPYTISGLVLVVPVTETHAHQAWAFLQPFSNSMWYTTAAFFFFTGTVVWILERDKNRDFGGRPRKQV
VTTFWFIFSTLFFSQRERINSILGRIVVIIWLFVVLILISSYTASLTSILTVRRLRPTIQGLSRLVGSDV
RIGYQEGSFVKDYLLQLNVESDRLVPLKSIATYSTALSSNEVGAVVDELPYVQLLLSSDCRFAISGEEEF
SKSGWGFAFPKGSALAADVSTAVLTLAETGELQRIHETWLHTTRCSGKVVEVKFDKLDLRAFSGLFGFFA
VVVVVATLSHALRSYCQNYKTALLAAAPFQWPSHFSRGSRFVRSFRSYVWQTGKNVSVA

>Ghir_D03G002390.1_HAU-AD1_v1.0 ID=Ghir_D03G002390.1_HAU-AD1_v1.0|Name=Ghir_D03G002390.1_HAU-AD1_v1.0|organism=Gossypium hirsutum|type=polypeptide|length=641bp
MSAKIFAFVLLLILSSKCSGIRDGDDQKDTEDDSCLMSCNTASDAHHIQL
KFNHSPGNVTPIDLYDDYQNYVAIKNLTSHRLPILCTVTSYETAFSAEIN
AFTRRAKANVLCALPAIDKATMGNESSTSYMSRHMYEIAREIASLIGDYL
WLKPGTTVYEESDRISDLDIVTFNLNSRNPTPNTGGIIQMAATLSFSTSK
PVHYYTEISIAVPVRSIPMQFLNISQDEKNHNEAQITGFWTDLFKEAVAV
MPINTTYKLVPFYGSDDQLFKALVRRTFDAAIGLTVMTRKGSELLEFSYP
YFEVGPMLVMKEKPEPNQVFSFMMPFTNEMWCTLAAMTMFNAFVIWLVES
RTGHESVGAIFWFPLATLFYGGHRESPRSNLTYFVLAPWLVLILVVSSTY
TQSFTSMITSSDTESSSCLDIEDLKKTNAIVGCDMEDSIMLQHLVEYIGF
QRKNIKHIAQSSIDDYAKALSTGKIKAAFFWAPYSGLFLAKYCKGFRSWG
PNHNLRGSSVIFPRDSPFAPYMSEAMVRLCGSGKFKRMKDDLQSFPECSS
STIDVTMKRGIGPGPFSGLFILSGTASAVAILITVIRPMRRRWERLVQGM
LMGRGLWVWLTTLFSRDQRGNQLQVQLARISFTSQTQLTSS

Downloaded from www.annualreviews.org. Guest (guest) IP: 129.2.89.114 On: Mon, 07 Oct 2024 14:58:42

SlGLR1.1	AB623193
SlGLR1.2	AB623194
SlGLR2.1	AB623195
SlGLR2.2	AB623196
SlGLR2.3	AB623197
SlGLR2.4	AB623198
SlGLR2.5	AB623199
SlGLR2.6	AB623200
SlGLR3.1	AB623201
SlGLR3.2	AB623202
SlGLR3.3	AB623203
SlGLR3.4	AB623204
SlGLR3.5	AB623205

ZmGLR1 XP_020399730.1
ZmGLR2 XP_020403253.1
ZmGLR3 XP_008670207.1
ZmGLR4 XP_008670208.1
ZmGLR5 XP_008670454.1
ZmGLR6 AQK55950.1
ZmGLR7 NP_001336839.1
ZmGLR8 XP_008644922.1
ZmGLR9 XP_008644925.1
ZmGLR10 XP_020393924.1
ZmGLR11 XP_008645171.4
ZmGLR12 XP_008646377.1
ZmGLR13 XP_008646378.1
ZmGLR14 XP_008651910.1
ZmGLR15 NP_001344128.1
ZmGLR16 ONM55135.1
ZmGLR17 ONM55137.1

SmGLR1 XP_024535362.1
SmGLR2 XP_002970053.1

OsGLR1.1	LOC_Os09g26144
OsGLR1.2	LOC_Os09g26160
OsGLR1.3	LOC_Os06g09130
OsGLR1.4	LOC_Os02g54640
OsGLR2.1	LOC_Os09g25980
OsGLR2.2	LOC_Os09g25960
OsGLR2.3	LOC_Os09g25990
OsGLR2.4	LOC_Os09g26000
OsGLR3.1	LOC_Os04g49570
OsGLR3.2	LOC_Os02g02540
OsGLR3.3	LOC_Os06g46670
OsGLR3.4	LOC_Os07g01310
OsGLR4	LOC_Os06g06130

MpGLR OAE25535

RsGluR AY328911

Downloaded from www.annualreviews.org. Guest (guest) IP: 129.2.89.114 On: Mon, 07 Oct 2024 14:58:42
